# Photo-activated digital resistive switching in a cryogenic photomemory

**DOI:** 10.1038/s41467-026-76121-2

**Published:** 2026-07-28

**Authors:** Jiarui Wang, Yu-Ting Huang, Yuhao Li, Meiyu Wang, Shengsheng Lin, Yuanhao Wei, Jingjing Chang, Yun Li, Fengqiu Wang, Songlin Li, Xian-Bin Li, Xinran Wang, Yi Shi, Zaiyao Fei

**Affiliations:** 1 National Laboratory of Solid-State Microstructures, School of Electronic Science and Engineering and Collaborative Innovation Center of Advanced Microstructures, Nanjing University, Nanjing, Jiangsu China; 2 State Key Laboratory of Integrated Optoelectronics, College of Electronic Science and Engineering, College of Integrated Circuits, Jilin University, Changchun, China; 3 National Key Laboratory of Spintronics, Nanjing University, Suzhou, Jiangsu China; 4 Center for Shared Scientific Research Facilities, Nanjing University, Nanjing, Jiangsu China; 5 Advanced Interdisciplinary Research Center for Flexible Electronics, Academy of Advanced Interdisciplinary Research, Xidian University, Xi’an, Shanxi China; 6 State Key Laboratory of Wide-Bandgap Semiconductor Devices and Integrated Technology, School of Microelectronics, Xidian University, Xi’an, Shanxi China; 7 School of Integrated Circuits, Nanjing University, Suzhou, Jiangsu China; 8 Suzhou Laboratory, Suzhou, Jiangsu China; 9 Interdisciplinary Research Center for Future Intelligent Chips (Chip-X), Nanjing University, Suzhou, Jiangsu China

**Keywords:** Electronic devices, Electronic properties and materials, Optoelectronic devices and components

## Abstract

Resistive switching is the basis of many emerging memory technologies. However, its operation at cryogenic temperatures remains scarce, and it generally lacks compatibility with optical control and in-situ electrical programmability. Here, we report the discovery of a photo-activated digital resistive switching effect in a cryogenic photomemory based on α-In_2_Se_3_. After an optical pulse, the device can be repeatably switched from a high- to a low-resistance state digitally, which persists long after the photoactivation. The switching threshold voltage can be continuously tuned over a wide range by the reset voltage pulse, additional optical pulses, and electrostatic gating, providing multimodal control over the memory logic. The device also exhibits a markedly enhanced photoresponse and resettable persistent photoconductivity, allowing the emulation of synaptic behaviors at cryogenic temperatures. Our work reveals a defect-mediated mechanism for embedding programmable digital logic into a nonvolatile photomemory, establishing a versatile platform for adaptive optoelectronics at cryogenic temperatures.

## Abstract (web summary)

While resistive switching serves as the promising foundation for next-generation memory technologies, its operation at cryogenic temperatures remains largely unexplored. Wang et al. report a photo-activated digital resistive switching effect at 2 K in lateral α-In₂Se₃ devices. Moreover, these devices emulate synaptic plasticity under such conditions.

## Introduction

The development of non-volatile memory technologies capable of operating at cryogenic temperatures is critical for advancing both classical cryogenic computing and quantum-classical hybrid systems^1,2^. While phase-change, ferroelectric and magnetic memories can retain states at cryogenic temperatures, they are generally not sensitive to optical inputs and offer limited in-situ tunability of their switching logic. Defect-mediated resistive switching materials are inherently sensitive to both electric fields and optical excitations, offering a potential route to overcome these limitations. In such systems, optical excitations can charge deep-level defects, leading to persistent changes in defect charge states and electrical conductivity when the illumination is terminated, known as persistent photoconductivity (PPC)^3–6^. These photo-activated defects may further migrate and reorganize under electric fields, giving rise to phenomena such as defect-mediated conduction paths^7^ and optically assisted field-driven transitions^8^. However, exploiting this dual responsivity to create a deterministic, digital memory at cryogenic temperatures, where defect dynamics are normally frozen, has not been achieved.

Layered α-In_2_Se_3_ provides a compelling material platform for realizing such coupled optoelectronic control, combining a visible-range bandgap, electrically switchable ferroelectric polarization^9–15^, and a high density of intrinsic defects that host both shallow and deep defect levels^16,17^. At room temperature, the interplay among these features has been exploited in photodetection^18–22^, ferroelectric modulation^17^, and resistive switching^23^. Yet its behavior under cryogenic conditions remains largely unexplored^24,25^.

In this study, we demonstrate that α-In_2_Se_3_ thin-film lateral devices exhibit an unexpected cooperative effect between light and bias at cryogenic temperatures, manifesting as a photo-activated, long-lived digital resistive switching behavior that is absent in the dark. Through temperature-dependent transport measurements and wavelength-selective excitation, we show that this behavior arises from light-activated and electrical field-driven migration of charged selenium vacancies near the contacts. We also show that the switching threshold voltage can be controlled by the reset voltage pulse, additional optical pulses and an electrostatic gate. This mechanism not only explains the observed digital switching, but also unifies the significantly enhanced photoresponse and electrically resettable PPC in the same frame.

## Results

### Temperature-dependent electrical measurements

**Fig. 1:**
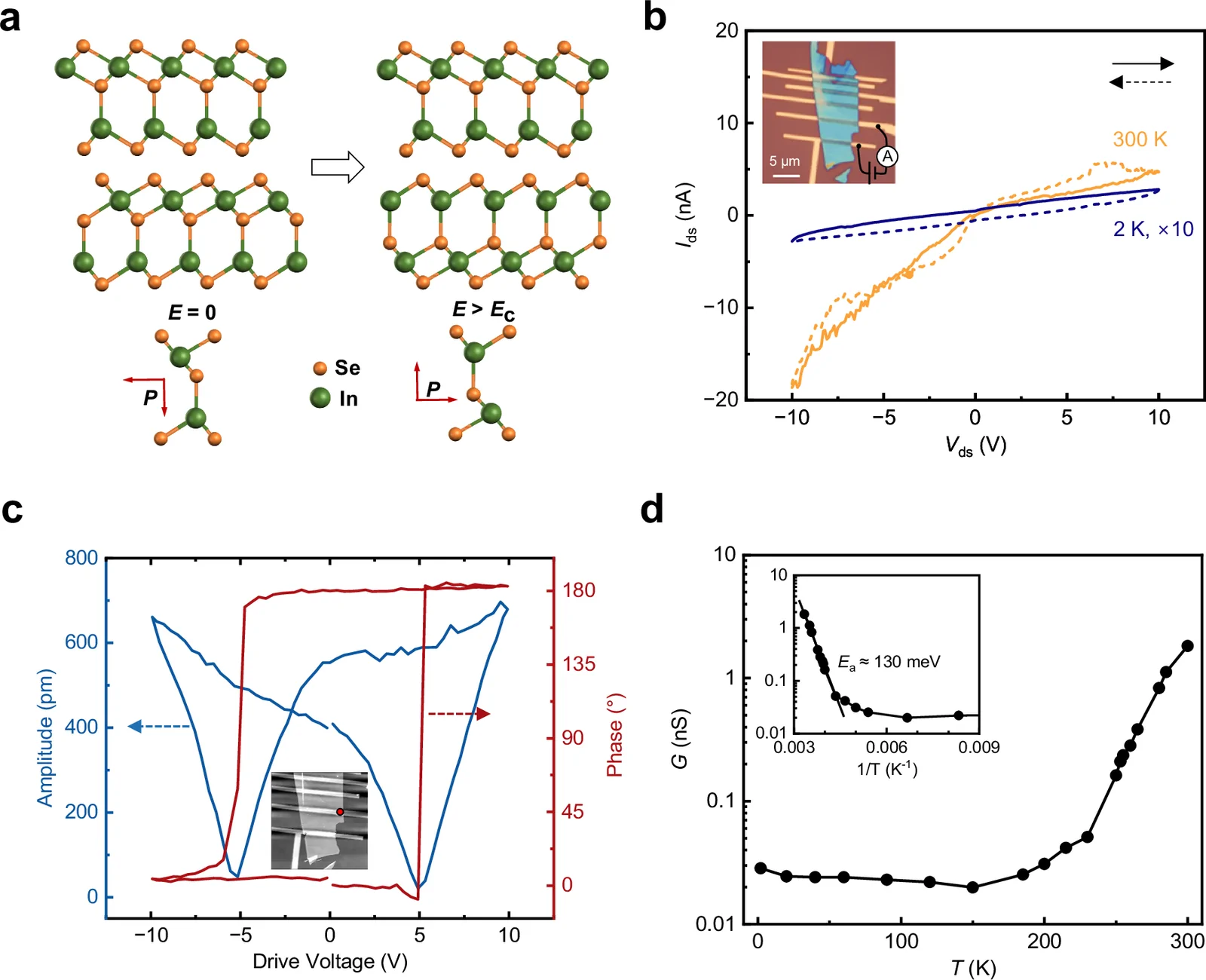
Ferroelectricity and electrical measurements of α-In_2_Se_3_ in the dark. **a** Schematic illustration of the atomic structure and polarization orientations of 2H-stacked α-In_2_Se_3_ before and after ferroelectric switching. **b** *I-V* characteristics of a two-terminal α-In_2_Se_3_ device at 300 K (purple) and 2 K (blue), respectively. The solid lines correspond to sweeps from −10 V to 10 V, while the dashed lines correspond to sweeps from 10 V to −10 V. The inset shows the optical image of device D1 and the measurement scheme. Scale bar: 5 μm. **c** Off-field out-of-plane piezoelectric response of α-In_2_Se_3_, where the phase switches 180° at ±5 V. Inset: topography of device D1, with single-point PFM performed at the location indicated by the red marker. **d** Linear conductance as a function of temperature. The inset shows the activation plot, where an activation energy of 130 meV is extrapolated.

We first look at the electrical transport of planar α-In_2_Se_3_ devices in the dark. These measurements allow us to distinguish conventional ferroelectric/charge trapping hysteresis from the photo-induced phenomena described later. Raman and photoluminescence measurements (Supplementary Fig. 1) confirm the crystal is in the α-phase with 2H stacking order^26–28^. In Fig. 1a, we illustrate the atomic lattices and electrical polarizations of the bottom layer before and after the application of a large in-plane electric field. The optical image of a typical device, device D1, is shown in the inset of Fig. 1b. It is made of a thin α-In_2_Se_3_ flake (32 nm thick) transferred onto prepatterned Au electrodes (see Methods for details of device fabrication). Figure 1c shows piezoresponse force microscopy (PFM) measurements of the flake. The off-field phase switches 180° at ±5 V, consistent with the out-of-plane polarization switching. In the following part of the paper, we focus on a particular pair of contacts with a channel length of ~5.5 μm, unless otherwise specified. The transfer curves of devices D1 and D2 in Supplementary Fig. 2 suggest that the α-In_2_Se_3_ crystal is initially n-doped.

The current-voltage (*I-V*) hysteresis at zero gate voltage is shown in Fig. 1b. The device exhibits high room-temperature resistance and pronounced nonlinearity, indicative of Schottky-like contacts at the electrode interface. Within a small bias window, upon increasing *V*_ds_ (sweep rate ~0.1 V s^‒1^ unless otherwise specified), the device transitions from a high-resistance state (HRS) to a low-resistance state (LRS). The LRS  is retained when the sweep direction reverses, even at small negative *V*_ds_, forming an eightwise hysteresis loop. Interestingly, when the bias range increases to ±10 V, a crossing point emerges near −5 V. Evolution of the crossing point as a function of the sweep range is provided in Supplementary Fig. 3. Such a crossing has been observed in another In_2_Se_3_ polymorph before and was attributed to the competition between ferroelectric switching and defect migration^29^.

Figure 1d shows the linear conductance as a function of temperature. As the device is cooled from room temperature, the conductance drops quickly, exhibiting a semiconducting behavior. From the activation plot shown in the inset of Fig. 1d, we extrapolate an activation energy of *E*_a_ = 130 meV. Below ~150 K, the conductance stops decreasing and even increases slightly at lower temperatures, which might be the residual conductance arising from the percolation transport between indium interstitials. The *I-V* sweep (Fig. 1b) also suggests the transport is dominated by charge trapping and detrapping into defect states at *T* = 2 K, as commonly observed in dielectrics. The detailed evolution of *I-V* hysteresis as a function of temperature is provided in Supplementary Fig. 4. It is worth noting that no abrupt transitions are observed at either room temperature or the base temperature, up to ±20 V (Supplementary Fig. 5).

### Cryogenic photoactivated digital resistive switching

**Fig. 2:**
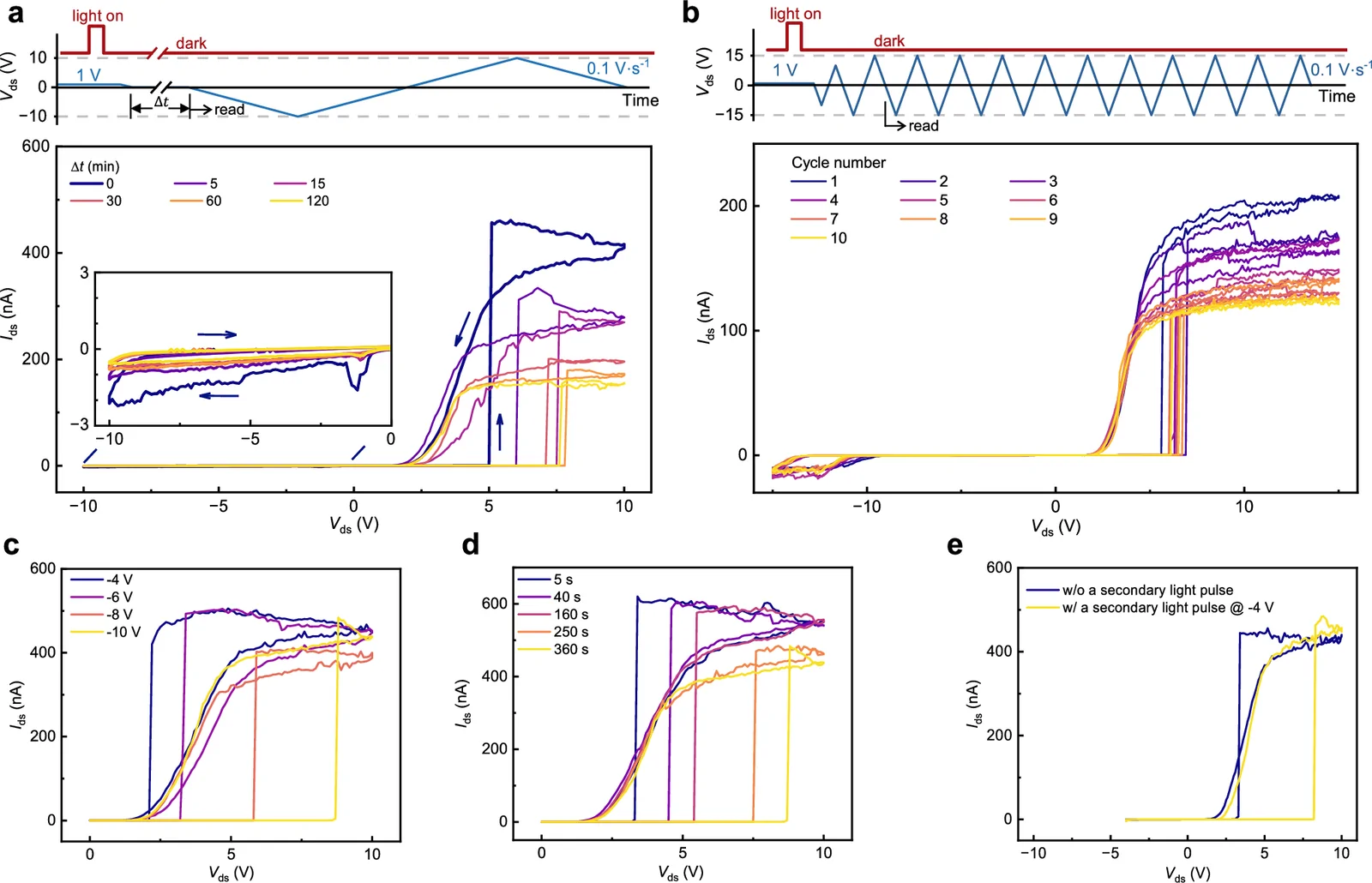
Photo-activated enduring digital resistive switching at *T* = 2 K. **a** *I-V* sweeps performed with varying delay times (Δ*t*) following the pulsed light illumination (0.14 mW cm^‒2^, 5 s). **b** Successive *I-V* sweeps following a single illuminating event. The top panels in (**a**, **b**) illustrate the timing of the light pulse and subsequent voltage sweeps. **c** *I-V* sweeps (0−10 V) performed after applying varying reset voltage pulses (−4, −6, −8, and −10 V) for 50 s. **d** *I-V* sweeps (0−10 V) performed after applying reset voltage pulses (−10 V) for varying amounts of time (5, 40, 160, 250, and 360 s). **e** Comparison of *I-V* sweeps (−4−10 V) with and without a secondary light pulse at −4 V (0.14 mW cm^‒2^, 5 s). For the sweep without a secondary light pulse, we waited for 5 s at −4 V.

We next examine the electrical response following optical activation. Figure 2a shows the *I-V* curve (highlighted in blue) of device D1 at 2 K after illuminating it for 5 s under *V*_ds_ = 1 V (white light LED, 0.14 mW cm^‒2^, unless otherwise specified; see Supplementary Fig. 6 for measurements with different incident powers and initial bias voltages, respectively). The top inset of Fig. 2a illustrates the timing of the pulsed light and subsequent voltage sweeps. After illumination, we record the current while sweeping the bias in the following order: 0, −10 V, 10 V, 0. The entire sweep takes about 6 minutes, and the obtained *I-V* curve is distinct from that in the dark (Fig. 1b). The most striking feature is the abrupt increase of current by more than three orders of magnitudes at around 5 V in the forward sweep (the slope is larger than 3 dec mV^‒1^, see Supplementary Fig. 7 for additional measurements), corresponding to the set transition from an HRS to an LRS. A negative differential resistance is observed after the sudden switching, which is commonly observed in memristors^30^. When a negative bias voltage is applied, the device is reset to the HRS, as shown in the enlarged plot in the bottom left inset of Fig. 2a. Restricting the bias to positive values after the set event retains the LRS (Supplementary Fig. 8a). Supplementary Fig. 8b presents repeated *I-V* sweeps, each preceded by an identical pulsed light stimulus. The threshold voltage of the set process can be fitted into a Gaussian distribution centered around 5 V with a standard deviation of ~1.2 V. This cryogenic photo-activated digital resistive switching behavior is reproducibly observed in a subset of α-In_2_Se_3_ devices that exhibit high and strongly nonlinear resistances at room temperature (see Supplementary Fig. 9 for data of devices D3–D5).

To illustrate the persistent effect of pulsed light on the resistive switching behavior, we perform similar measurements with varying delay times after the illumination. The corresponding *I-V* curves are shown in Fig. 2a. As the delay time increases from 5 to 120 min, the LRS current decreases while the threshold voltage increases. Nevertheless, the digital resistive switching behavior persists, indicating that the pulsed light effect can persist for hours (the maximum delay tested was 12 h; see Supplementary Fig. 10). The persistence of the photo-activated behavior is further demonstrated through successive *I-V* sweeps following a single illumination event, with stable switching features maintained for over 10 cycles (Fig. 2b). Note that the voltage sweep range differs from that in Fig. 2a.

To assess the potential of this photo-activated switching for non-volatile memory applications, we evaluate the key memory metrics including retention, endurance and switching speed. Supplementary Fig. 11 presents retention and endurance tests of the device measured at *V*_ds_ = 4 V. After 10,000 s and 100 cycles, the current ratio between LRS and HRS remains high at 10^3^. The switching speed was characterized on a similar device (device D6) by varying the set pulse duration at different amplitudes (see Supplementary Fig. 12). With a pulse amplitude of 18 V, the device switches from HRS to LRS within 500 ns. For larger pulse amplitudes, the switching timescale is expected to be even shorter. Such sub-microsecond switching speeds are among the fastest reported for memristors operating at cryogenic temperatures (Supplementary Table 1).

The threshold voltage for the set process (*V*_set_) can be non-volatilely tuned by controlling the reset process. For simplicity, instead of sweeping the bias to different negative values, we apply reset voltage pulses with different amplitudes and durations. As the amplitude of the reset voltage pulse increases from −4 V to −10 V while fixing the pulse duration to 50 s, *V*_set_ increases from 2.1 V to 8.7 V (Fig. 2c), much larger than the standard deviation in Supplementary Fig. 8b. In Fig. 2d, when we fix the reset pulse amplitude to −10 V and vary the pulse duration from 5 s to 360 s, *V*_set_ increases from 3.3 V to 8.7 V. These experiments suggest that one could delay or suppress the set process by applying a reset voltage with either larger amplitude or longer duration. More interestingly, we find that the set process can also be suppressed by applying additional light pulses at negative bias voltages. Figure 2e compares *I-V* curves with and without a secondary light pulse at -4 V (lasts for 5 s, we also waited for 5 s at −4 V for the sweep without a secondary light pulse), *V*_set_ increases from 3.3 V to 8.1 V.

### Dependence on gate voltage and temperature

**Fig. 3:**
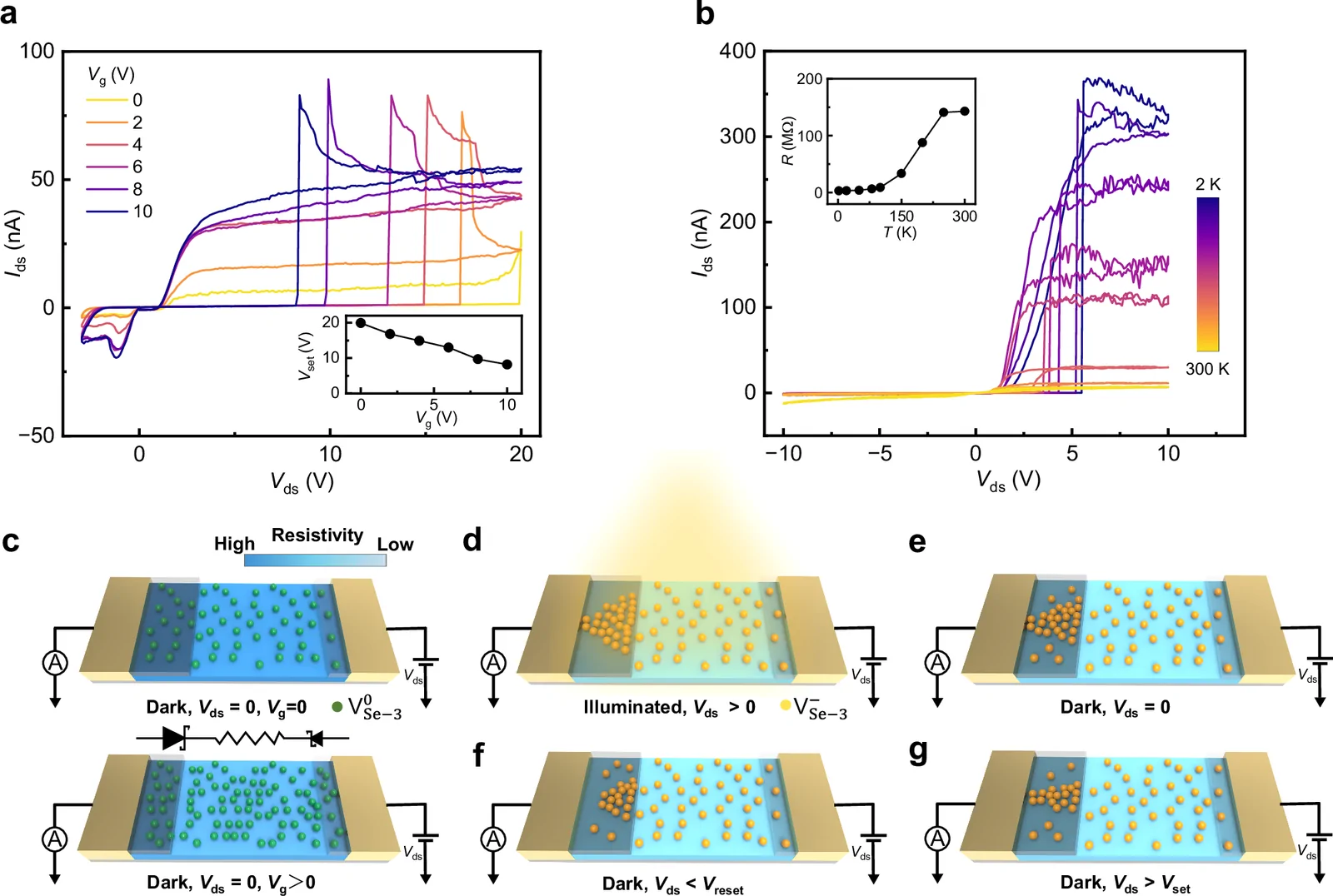
Gate tunability and temperature dependence of the photo-activated resistive switching effect. **a** Gate-voltage dependence of the photo-activated resistive switching in a back-gated device (device D2). The inset shows the *V*_g_ dependence of *V*_set_. **b** Photo-activated resistive switching measured at selected temperatures (2, 20, 50, 100, 150, 200, 250 and 300 K). The inset shows the temperature-dependent resistance of the LRS. **c** Schematic illustration of the lateral device under zero (upper inset) and positive (lower inset) *V*_g_, where the Schottky-like contacts region decreases for *V*_g_ > 0. **d**–**g** Schematics of the microscopic processes for the photo-activated digital resistive switching in α-In_2_Se_3_ lateral devices. The light green and yellow spheres represent neutral and negatively charged selenium vacancies, respectively. The shaded region near the left Au electrode indicates the Schottky-like barrier.

The digital switching process can also be continuously tuned by an electrostatic gate. Figure 3a shows the *I-V* characteristics of a back-gated α-In_2_Se_3_ device (device D2), with a few-layer graphene serving as the gate, isolated from the channel by a thin hBN dielectric. At *V*_g_ = 0, *V*_set_ is measured to be 19.9 V. Under electron doping (*V*_g_ > 0), *V*_set_ decreases rapidly, reaching 8.2 V for *V*_g_ = 10 V. Under hole doping (*V*_g_ < 0), *V*_set_ increases beyond the safe bias range. Importantly, applying *V*_g_ as a transient pulse before the *I-V* sweep has negligible effect on the switching characteristics (Supplementary Fig. 13). This indicates that the gate tuning is volatile, distinct from that of bias pulse tuning, and instead electrostatically modulates the channel conditions in real time to set the switching threshold.

We also thermal-cycle device D1 and measure the *I-V* characteristics at elevated temperatures. As temperature increases, the threshold voltage for the set process decreases (Fig. 3b). We observe that the LRS conductance decreases with increasing temperature (see the inset of Fig. 3b). To exclude the temperature-dependent PPC effect, we measure the conductance of device D1 at different temperatures with light consistently on (Supplementary Fig. 14). The result corroborates the metallic temperature dependence. The resistive switching becomes more gradual above 150 K due to thermal broadening, and is indistinguishable from the ferroelectric-induced resistance switching at 300 K. We would like to point out that the threshold voltages can vary at the same temperature of different thermal cycles.

To gain further insights into the photo-activated resistive switching effect, we try light sources with different wavelengths. Supplementary Fig. 15 shows *I-V* curves at 2 K illuminated with different pulsed lights of similar power density. Similar set/reset features and threshold voltages are observed when illuminated by pulsed white, 633 nm and 1050 nm light, in which the 633 nm light appears to generate the largest LRS current. On the other hand, when illuminated with a 1200 nm light of similar intensity, the device remains at the HRS. The contrasting behaviors suggest the activation of digital resistive switching is highly related to the defect states close to the valence band in α-In_2_Se_3_^17^.

### Model of photoactivated digital resistive switching

The photo-activated digital resistive switching observed here could, in principle, originate from two distinct microscopic processes: (i) a defect-mediated reorganization that creates localized conductive channels, or (ii) a defect-facilitated reversal of ferroelectric domains that drastically lowers the contact barrier. Critical experimental observations, however, strongly favor the former. First, the sharpness of the transition (3 dec mV^‒1^) and the subsequent negative differential resistance are characteristics of formation of percolative conductive channels, not typically observed in the more gradual resistance modulation of ferroelectric switching (see gate dependences in Supplementary Fig. 2b). Second, the volatile nature of gate-tunability of *V*_set_ aligns with electrostatic control of defect-mediated reorganization process, whereas gate tuning of ferroelectricity is expected to be non-volatile due to the coupled in-plane and out-of-plane ferroelectric order in α-In_2_Se_3_. Nevertheless, the present evidence does not exclude a contribution from ferroelectric switching, and the interplay between defect dynamics and ferroelectric polarization remains an important open question that warrants further investigation.

We therefore propose a model in which photo-activated and field-controlled migration of charged selenium vacancies leads to the formation and rupture of conductive paths near the contacts^31^. Our scanning transmission electron microscopy (STEM) and density functional theory (DFT) results indicate that V_Se-3_ defects are present and are energetically favorable (Supplementary Figs. 16, 17). Upon above-bandgap illumination, V_Se-3_ defects are expected to become negatively charged through the trapping of photogenerated carriers. DFT calculations further yield a migration barrier of approximately 0.76 eV for V_Se-3_ (Supplementary Fig. 18). In the presence of strong local electric fields near the Schottky contacts and photoinduced changes in defect charge state, migration of these defects near the contacts is expected to be energetically accessible despite the micrometer-scale channel length^32^ (upper inset of Fig. 3c). The abundant photogenerated electron-hole pairs may also lower the migration barrier for these charged vacancies by modulating the potential energy landscape. Within this picture, charged selenium vacancies can accumulate near one of the contacts (Fig. 3d) and reorganize to create locally percolating conduction channels. Once established, these conducting paths persist long after the light is removed (Fig. 3e). A subsequent negative bias ruptures or depletes the vacancy accumulation (Fig. 3f), resetting the device to its HRS. When the bias is swept back to a positive value above the threshold voltage, the conductive paths reform (Fig. 3g), giving rise to the observed digital switching. In fact, field-driven ion migration over comparable barriers has been reported in several memristive systems under strong local electric fields at cryogenic temperatures^33,34^.

The gate tunability of *V*_set_ arises from the effect of *V*_g_ on the spatial dimension for defect aggregation. A positive *V*_g_ reduces the width of the Schottky barrier near the contacts due to band bending (lower inset of Fig. 3c), which lowers the local field needed to form conductive channels. Concurrently, the doped electrons in the channel may also electrostatically screen the repulsive interactions between the negatively charged selenium vacancies, thereby cooperatively lowering the migration barrier and *V*_set_.

### Photoresponse and synaptic behaviors at low temperatures

**Fig. 4:**
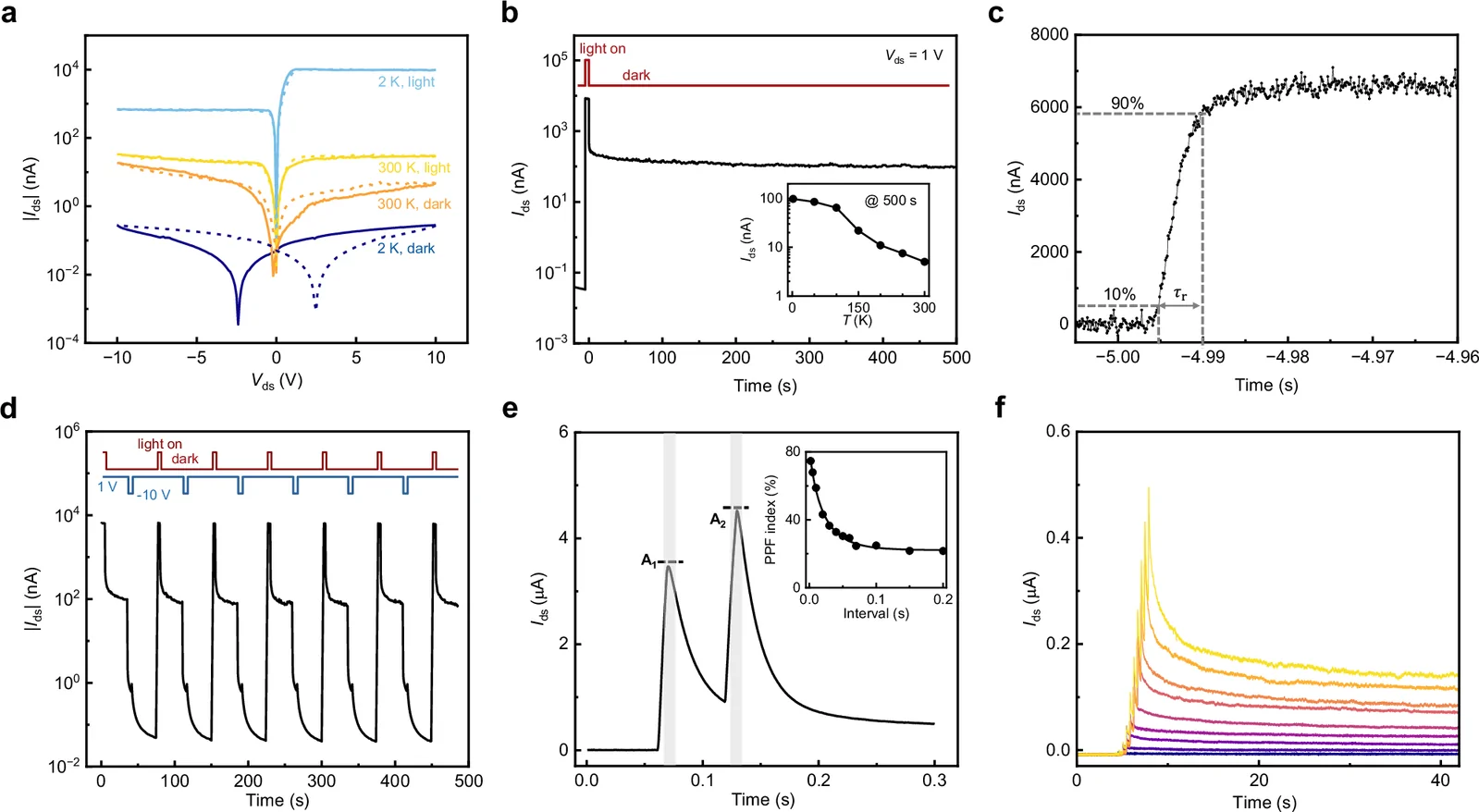
Photoresponse and synaptic behaviors of α-In_2_Se_3_ at cryogenic temperatures. **a** Semilog *I-V* characteristics measured under different conditions: yellow, 300 K with light on; orange; 300 K in the dark; light blue, 2 K with light on; dark blue, 2 K in the dark. **b** Main panel: Transient photoresponse measured at *V*_ds_ = 1 V, *T* = 2 K. The device is illuminated for 5 s by a white light LED with a power density of 0.14 mW cm^‒2^, a persistent photoconductivity is observed when the light is turned off. Bottom right inset: Current measured 500 s after the light is off as a function of temperature. **c** Determination of the rise time in (**b**). **d** Transient photoresponse with alternating optical and electrical stimuli. Top inset: Timing of optical (0.14 mW cm^‒2^, 5 s) and electrical (−10 V, 5 s) pulse trains. **e** Light-induced PPF effect, where a pair of light pulses is applied (0.14 mW cm^‒2^, 10 ms, with an interval of 50 ms), the read bias voltage is 1 V. Inset: PPF ratio as a function of the pulse interval, with the solid line representing a stretched exponential decay fit. **f** SNDP demonstration of the device, showing transition from STM to LTM.

We now examine how constant illumination modifies the transport at low temperatures. Figure 4a compares *I-V* curves of D1 in the dark and under illumination. For the same bias (*V*_ds_ = 1 V), the dark current is suppressed by ~20 times at *T* = 2 K compared to room temperature, whereas under illumination the current is enhanced by 650 times. Accordingly, the photoresponsivity *R* = *I*_ph_/*P* is estimated to increase from 167 A W^‒1^ to 110,000 A W^‒1^. The specific detectivity D^*^ = $R\sqrt{A\Delta f}/{I}_{n}$ at *T* = 2 K is estimated to be 1.8 × 10^16^ Jones, assuming the dark current noise (*I*_n_) is dominated by shot noise (see Supplementary Note 1 for more information). Thus, the sensitivity of the device to light is significantly improved after cooling, which aligns with the formation of conductive channels near the contacts after photoactivation. Supplementary Table 2 compares the photoresponsivity and specific detectivity of different α-In_2_Se_3_-based photodetectors.

We also characterize the transient photoresponse at various temperatures. The white light is switched on and off by a voltage pulse. As shown in the main panel of Fig. 4b, the current drops quickly within 100 ms at 2 K after the light is switched off. However, even after 500 s, the current still retains a significantly higher level than the baseline in the dark, suggesting the presence of PPC with a long lifetime. The temperature dependence of the current at 500 s after illumination is plotted in the bottom right inset of Fig. 4b. As temperature decreases, the PPC increases by more than 10 times. In Fig. 4c, we show zoom-in measurement of the rising process, from which we determine the rise time to be 5.8 ms (time for the current to rise from 10% to 90% of the saturation value). Analysis and discussion of the falling process are provided in Supplementary Fig. 19. The transient photoresponse suggests that the device can retain the photoinduced conductance state for an extended period at cryogenic temperatures, thereby functioning as a cryogenic photomemory. The key performance metrics are benchmarked against representative cryogenic photomemory systems in Supplementary Table 3, providing a broader context for evaluating its retention characteristics and optical programming condition. More importantly, the memory state in the present device is characterized not only by persistent photoconductivity, but also by a long-lived photo-activated digital resistive switching capability, distinguishing it from conventional cryogenic photomemory systems.

The PPC effect is usually undesired for photodetectors. For our α-In_2_Se_3_ devices, we can reset the conductivity by rupturing the conductive channels with a large negative bias. Figure 4d shows the response of D1 with alternating light and electrical stimuli. The −10 V electrical pulses (last for 5 s) can quickly reset the persistent current from ~100 nA to below 0.1 nA, the same level as the dark current at 2 K (Fig. 1b). When light is turned on again, the current increases to the initial value quickly. Similar behaviors are observed in multiple cycles, suggesting the reliability of the device as a photodetector.

We then study the synaptic plasticity of device under electrical and optical stimuli. Figure 4e shows the post-synaptic current under a pair of light pulses, separated by 50 ms and lasting for 10 ms each. A small bias of 1 V is applied to read the conductance of the device. Two current spikes (A_1_ and A_2_, A_2_ > A_1_) are observed, indicating the paired-pulse facilitation (PPF) effect. The PPF index ((A_2_-A_1_)/A_1_) as a function of the time interval between the paired pulses is shown in the inset of Fig. 4e. The PPF index can be fitted into a double exponential function: ${C}_{0}+{C}_{1}\exp \left(-{t/\tau }_{1}\right)+{C}_{2}\exp (-t/{\tau }_{2})$, where *τ*_1_ and *τ*_2_ are characteristic relaxation times. The best fit yields *τ*_1_ = 15.7 ms and *τ*_2_ = 71.7 ms. Figure 4f shows the spike-number-dependent plasticity (SNDP) of the same device. As the number of identical light pulses increases, the long-term conductance increases, corresponding to the transition from a short-term memory (STM) to a long-term memory (LTM). Additional demonstrations of the spike-timing-dependent plasticity, spike-frequency-dependent plasticity and spike-amplitude-dependent plasticity of the same device can be found in Supplementary Fig. 20.

Supplementary Fig. 21a plots the conductance as a function of the number of optical or electrical pulses, exhibiting optical potentiation and electrical depression functionalities. With the obtained multiple conductance levels, we simulate the image recognition by building a three-layer artificial neural network (ANN), consisting of 784 input neurons for modified National Institute of Standards and Technology (MNIST) images with 28 × 28 pixels, 128 hidden neurons and 10 output neurons for digits ranging from 0 to 9. We first trained the ANN with 60,000 images randomly chosen from the MNIST dataset. Following each training epoch, the image recognition accuracy and loss are evaluated with 10000 distinct random images from the MNIST dataset. Over 100 epochs, the recognition accuracy increases while the loss decreases, reaching 91% and 0.32, respectively. More information about the simulation can be found in Supplementary Fig. 21.

## Discussion

In summary, we report a photo-activated digital resistive switching effect in α-In_2_Se_3_ that persists for extended periods at cryogenic temperatures. We attribute this phenomenon to photo-activated and field-driven migration of selenium vacancies, which create conductive paths near the contact interfaces. The process can be dynamically modulated by optical pulses, reset voltage pulses, and an electrostatic gate, providing versatile control over the device’s logic. These capabilities enable photo-addressable cryogenic digital memory with long retention, high endurance, and electrically tunable switching thresholds. In addition, the same physical mechanism gives rise to dramatically enhanced photoresponse, controllable PPC and synaptic functionalities. Together, these results highlight the potential of α-In_2_Se_3_ for multifunctional cryogenic optoelectronics, where memory, sensing, and signal-processing capabilities can be realized within a single cryogenic platform.

## Methods

### Device fabrication

α-In_2_Se_3_ crystals are sourced from HQ Graphene. For most devices (including device D1, D2), prepatterned Cr/Au (7/70 nm) electrodes were defined via e-beam lithography and thermal evaporation on 285 nm SiO_2_/Si or hBN substrates. Selected α-In_2_Se_3_ flakes were then transferred onto the electrodes with a dry transfer technique inside an oxygen-free glovebox. For other devices, α-In_2_Se_3_ flakes are mechanically exfoliated onto SiO_2_ substrates directly inside the glovebox, followed by deposition of Cr/Au electrodes on them. We have also tried to use hexagonal boron nitride flakes to encapsulate the α-In_2_Se_3_ flakes in device D3 to minimize further degradation. α-In_2_Se_3_ samples for STEM measurements were picked up by PVA stamps, then dropped down onto a silicon nitride support grid (with a 15-nm-thick membrane on the grid holes) at 160 °C.

### Material characterizations

Raman and photoluminescence spectra of exfoliated α-In_2_Se_3_ flakes on SiO_2_ substrate were obtained on a homemade imaging system equipped with a Horiba iHR320 spectrometer and an excitation laser of 532 nm. Atomic force microscopy and piezoresponse force microscopy were carried out on an Oxford Instruments Asylum Research MFP-3D Origin AFM at ambient conditions. ASYELEC-01-R2 probes coated with 5-nm Ti and 20-nm Ir were used.

### Electrical and optoelectronic measurements

All electrical and optoelectronic measurements were performed in-situ under high vacuum across a temperature range of 2–300 K using a Quantum Design Opticool system. In the electrical measurements and most of the optoelectronic measurements, the *I-V* curves were obtained using a Keithley 2636b Source Measure Unit. The transient photocurrent (particularly for the fast response) was measured with a Femto DLPCA-200 current amplifier and a USB-6212 NI DAQ device. Four different light sources are used in the optoelectronic measurements, i.e. a white light (Thorlabs MNWHL4), a 633 nm He-Ne laser, a 1050 nm LED (50 nm FWHM), and a 1200 nm LED (70 nm FWHM). For the white light LED, we controlled the exposure time by an electrical pulse generated by USB-6212.

### Scanning transmission electron microscopy measurements

High-angle annular dark-field scanning transmission electron microscopy (HAADF-STEM) images were acquired using an FEI Titan Cubed G2 60−300 aberration-corrected S/TEM operated at an accelerating voltage of 300 kV, with a probe convergent semi-angle of 22.5 mrad, a beam current of 0.06 nA, and a camera length of 73 mm.

### Density functional theory calculations

DFT calculations were performed using the Vienna Ab initio Simulation Package (VASP)^35^. The projector augmented wave pseudopotential and Perdew-Burke-Ernzerhof exchange correlation functional were used^36,37^. The valence electrons considered included In (4*d*^*10*^5*s*^*2*^5*p*^1^) and Se (4*s*^*2*^4*p*^4^). A vacuum layer of more than 20 Å was introduced in the out-of-plane direction to minimize spurious interactions between periodic images. The migration energy barrier of the Se-3 vacancy (V_Se-3_) was calculated using the climbing image nudged elastic band (CI-NEB) method^38^. A 5 × 5 × 1 supercell containing 124 atoms (incorporating one Se-3 vacancy) was employed. Structural relaxations were performed until the forces on each atom converged to less than 0.02 eVÅ^‒1^.

The formation energies for the five types of vacancy defects were calculated using the following equation: $\Delta H\left(w\right)={E}_{{{\rm{tot}}}}\left(w\right)-{E}_{{{\rm{tot}}}}\left({{\rm{host}}}\right)+({E}_{i}+{\mu }_{i})$. Here, ${E}_{{{\rm{tot}}}}\left(w\right)$ is the total energy of the 124-atom supercell containing a specific vacancy defect, and ${E}_{{{\rm{tot}}}}({{\rm{host}}})$ is the total energy of the pristine 125-atoms supercell. The term $({E}_{i}+{\mu }_{i})$ accounts for the chemical potential changes caused by atomic substitution during defect formation. ${E}_{i}$ represent the chemical potential of element i in its most stable elementary state, and ${\mu }_{i}$ indicates the relative chemical potential, capturing the influence of environmental conditions on the defect formation process. In our calculations, both In-rich and Se-rich limits were considered. The chemical potential ranges were strictly constrained to avoid the precipitation of pure elemental In or Se, as well as the formation of the common competing secondary phase, InSe. Finally, the points along the solid blue line in Supplementary Fig. 17a align with all of the requirements.

## Supplementary information


Supplementary Information
Transparent Peer Review file


## Data Availability

Source Data has been deposited in figshare under accession code (https://doi.org/10.6084/m9.figshare.30415576).
