# Supplementary material for: Photo-activated digital resistive switching in a cryogenic photomemory
Source: Nat Commun. 2026 Jul 28;17:9167. doi: 10.1038/s41467-026-76121-2 (PMC13522526; doi:10.1038/s41467-026-76121-2)
Supplement: Supplementary file 1 [file 41467_2026_76121_MOESM1_ESM.pdf]

Supporting Information for

Photo-activated digital resistive switching in a cryogenic photomemory

Jiarui Wang[1], Yu-Ting Huang[2], Yuhao Li[1,3], Meiyu Wang[4], Shengsheng Lin[1], Yuanhao Wei[1], Jingjing Chang[5,6], Yun Li[1], Fengqiu Wang[1], Songlin Li[1], Xian-Bin Li[2], Xinran Wang[1,7,8,9,*], Yi Shi[1,*], Zaiyao Fei[1,3,*]

1. National Laboratory of Solid-State Microstructures, School of Electronic Science and Engineering and Collaborative Innovation Center of Advanced Microstructures, Nanjing University, Nanjing 210093, Jiangsu, China
2. State Key Laboratory of Integrated Optoelectronics, College of Electronic Science and Engineering, Jilin University, Changchun 130012, Jilin, China
3. National Key Laboratory of Spintronics, Nanjing University, Suzhou 215163, Jiangsu, China
4. Center for Shared Scientific Research Facilities, Nanjing University, Nanjing 210023, Jiangsu, China
5. Advanced Interdisciplinary Research Center for Flexible Electronics, Academy of Advanced Interdisciplinary Research, Xidian University, Xi'an 710071, Shanxi, China
6. State Key Laboratory of Wide-Bandgap Semiconductor Devices and Integrated Technology, School of Microelectronics, Xidian University, Xi'an 710071, Shanxi, China
7. School of Integrated Circuits, Nanjing University, Suzhou 215163, Jiangsu, China
8. Suzhou Laboratory, Suzhou 215123, Jiangsu, China
9. Interdisciplinary Research Center for Future Intelligent Chips (Chip-X), Nanjing University, Suzhou 215163, Jiangsu, China

[*]Correspondence to: xrwang@nju.edu.cn, yshi@nju.edu.cn, zyfei@nju.edu.cn

Contents:

Supplementary Figures 1-21
Supplementary Tables 1-3
Supplementary Note 1
Supplementary References

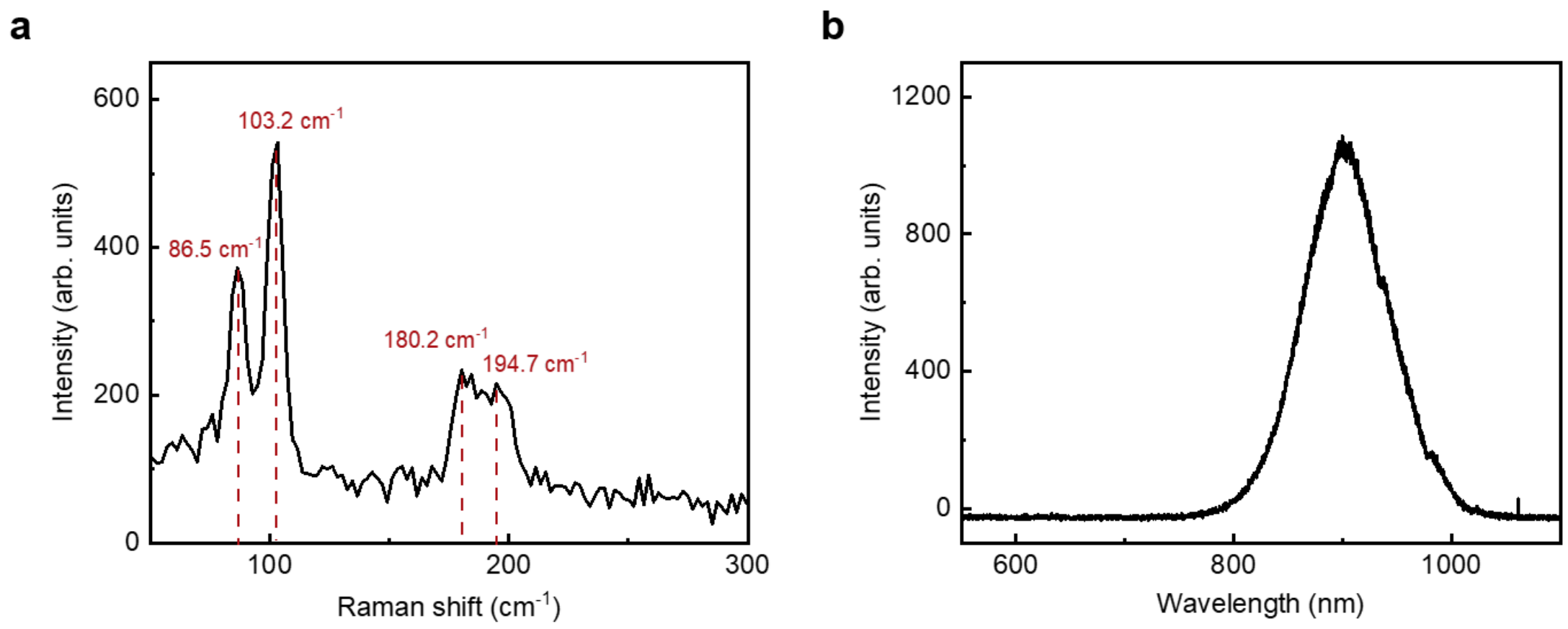

Supplementary Fig. 1 | Raman (a) and photoluminescence (b) spectra of the crystal. The A_1^1 peak appears at 103.2 cm^{-1}, earlier than in the β- and γ-phases (> 110 cm^{-1}). The absence of peaks beyond 200 cm^{-1} confirms the α-phase, while the strong E^2 peak (86.5 cm^{-1}), exceeding half the A_1^1 peak intensity, suggest the 2H polytype[1,2].

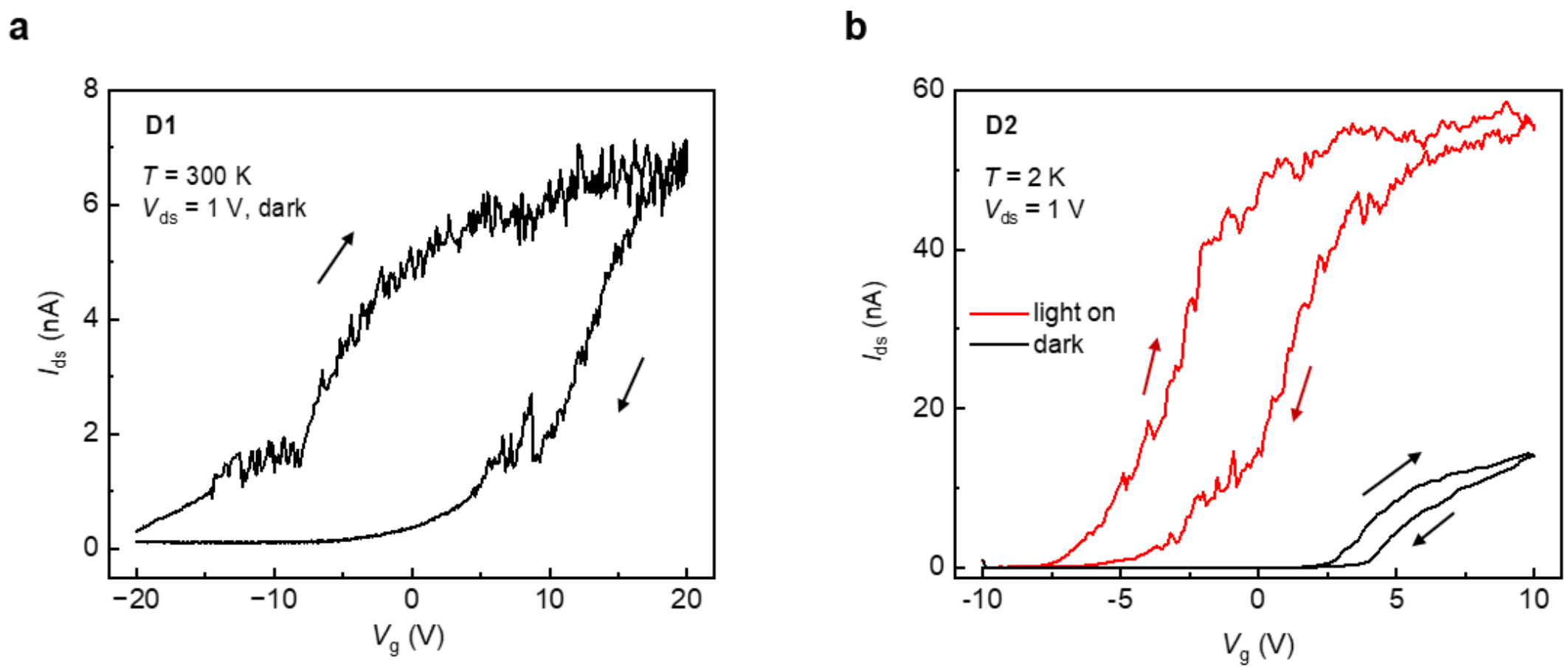

Supplementary Fig. 2 | Transfer characteristics of devices D1 & D2. a, At room temperature, the clockwise hysteresis loop of device D1 is consistent with reported behavior of α-In_2Se_3 ferroelectric semiconductor field-effect transistor with a high effective oxide thickness[3]. **b**, Transfer characteristics of device D2 at 2 K measured in dark and under continuous illumination, respectively. No abrupt transitions are observed during ferroelectric switching.

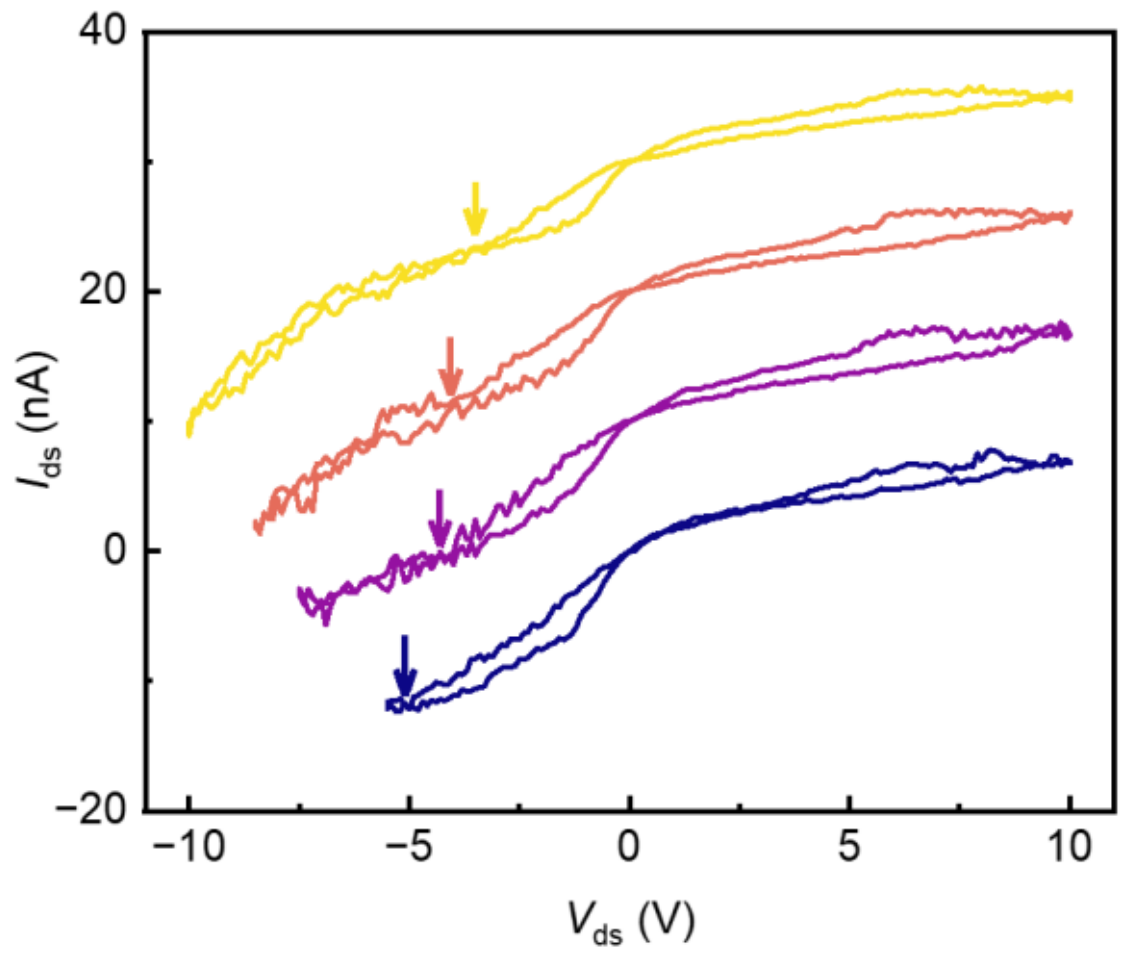

Supplementary Fig. 3 | *I*–*V* characteristics of device D1 at room temperature under different voltage sweep ranges. The curves (vertically offset for clarity) correspond to sweep ranges of –5.5, –7.5, –8.5, and –10 V to 10 V, respectively. As the sweep range increases, the crossing voltage becomes less negative (marked by the arrows).

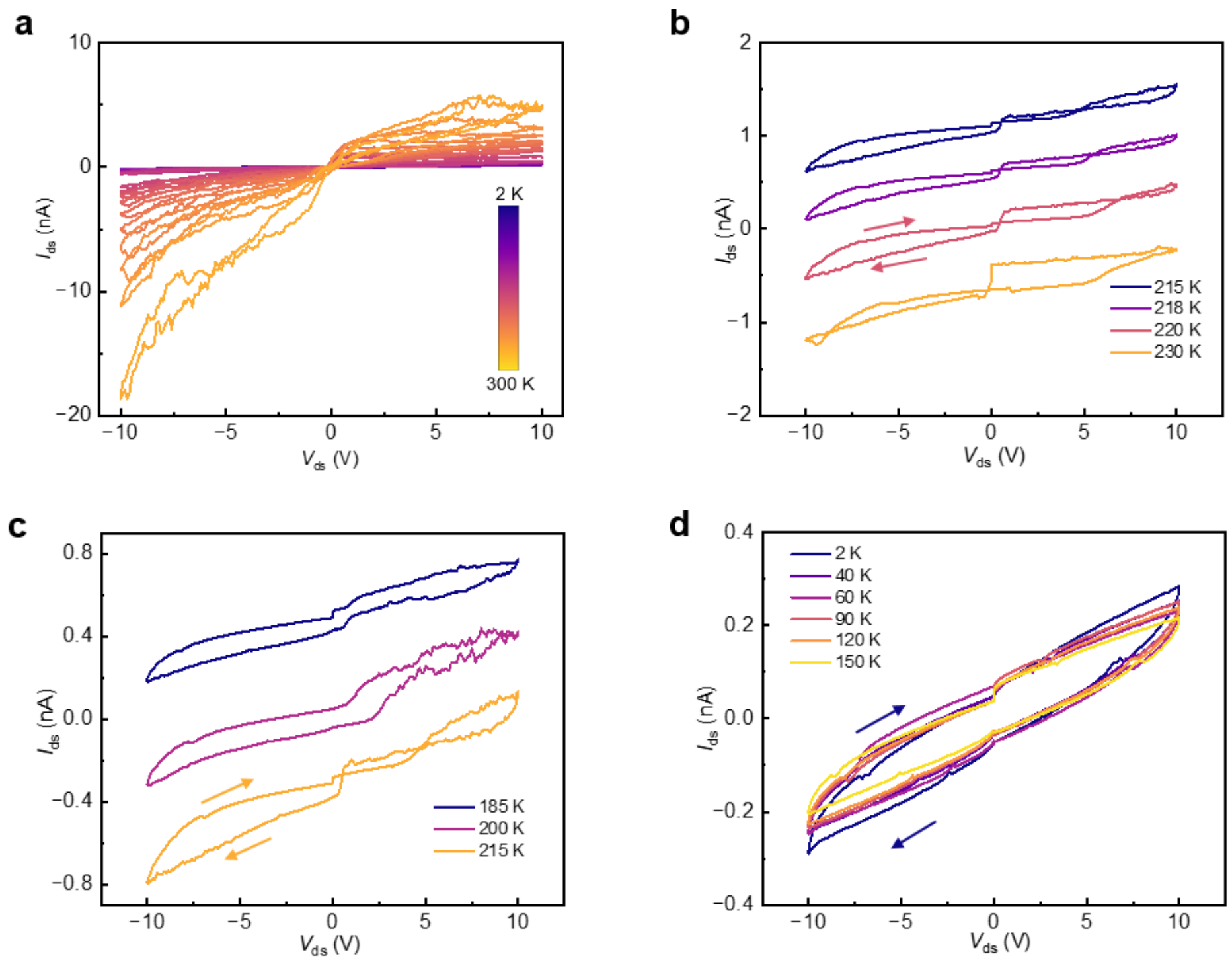

Supplementary Fig. 4 | Temperature-dependent Evolution of I–V characteristics of device D1 in the dark. During the initial cooling stage (300-230 K), the resistance increases gradually, and overall sweep resembles that at 300 K. Upon further cooling (**b**), the crossover at negative bias disappears, while a crossover emerges at positive bias and becomes more pronounced with decreasing temperature. With continued cooling (**c**), the positive-bias crossover also diminishes. Below 185 K (**d**), the *I–V* characteristics predominantly exhibit a clockwise hysteresis loop, accompanied by a slight decrease in resistance.

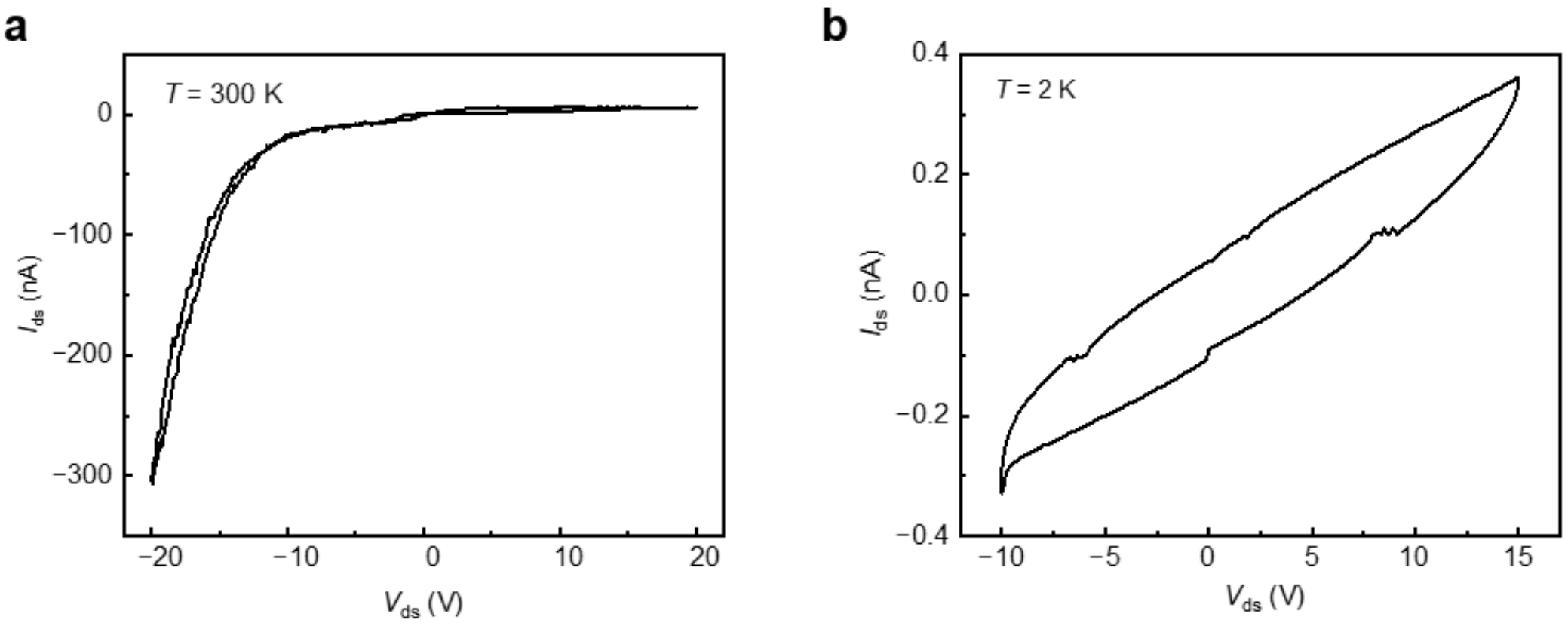

Supplementary Fig. 5 | *I*–*V* characteristics of device D1 over a large sweep range in the dark at *T* = 300 K (a) and 2 K (b).

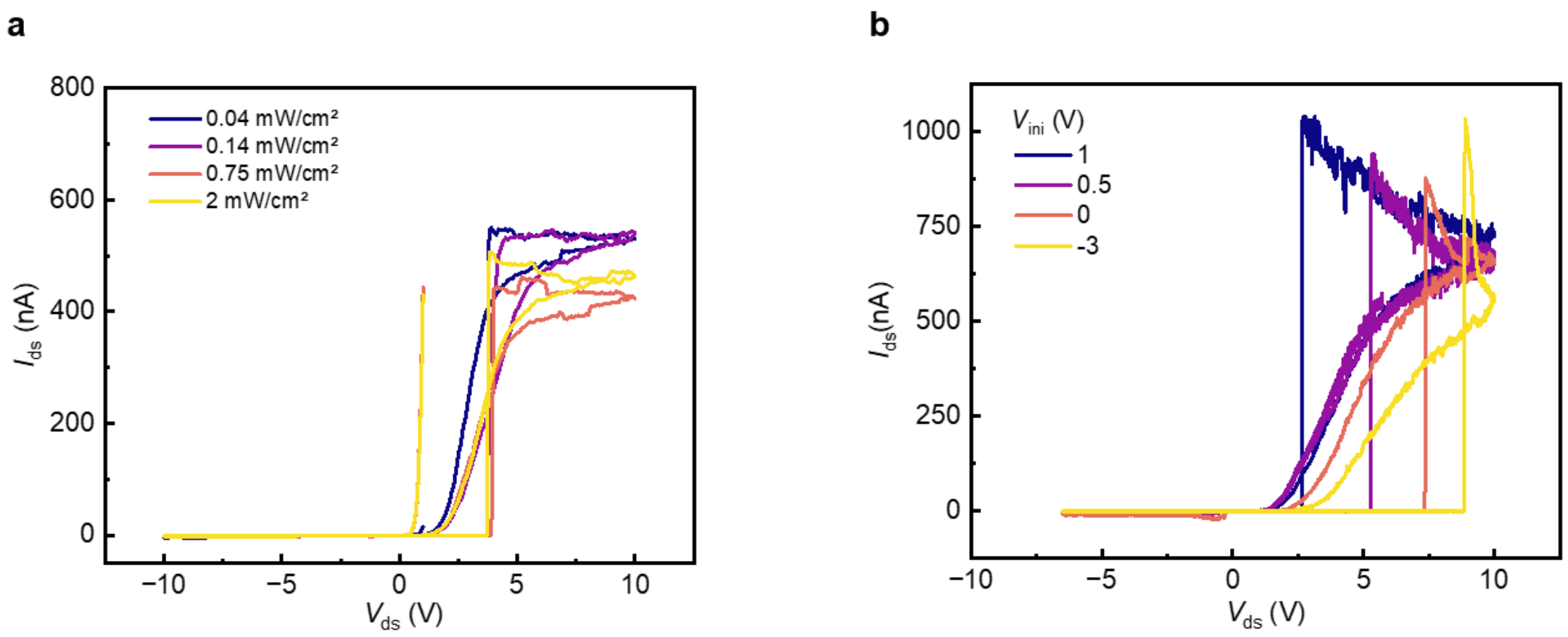

Supplementary Fig. 6 | Resistive switching of device D1 following pulsed illumination at different power densities (a) and initial bias voltages (b). The threshold voltage and the LRS current shows only weak dependence on the applied light intensity, whereas the switching process is facilitated when the device is illuminated under a larger positive bias.

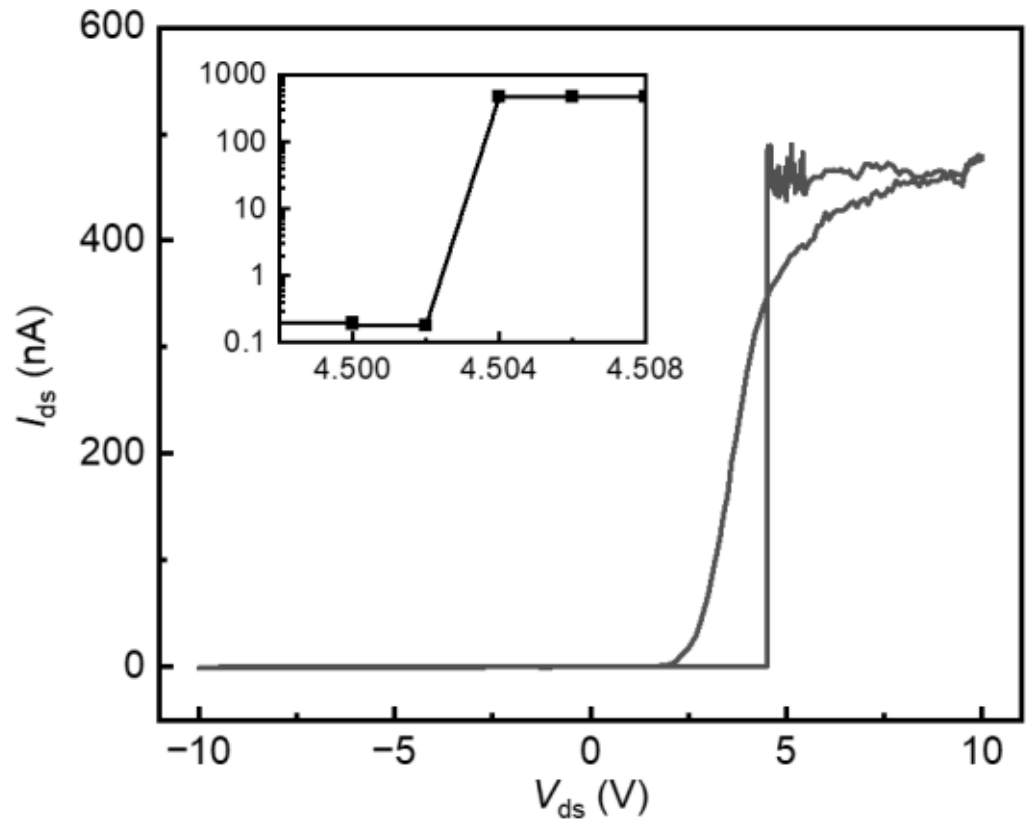

Supplementary Fig. 7 | Photo-activated digital resistive switching at a small sweep rate of 2 mV s^{-1} near the transition. The inset highlights the abrupt transition, showing that the current increases by more than three orders of magnitude within a 2 mV bias interval (3 dec mV^{-1}).

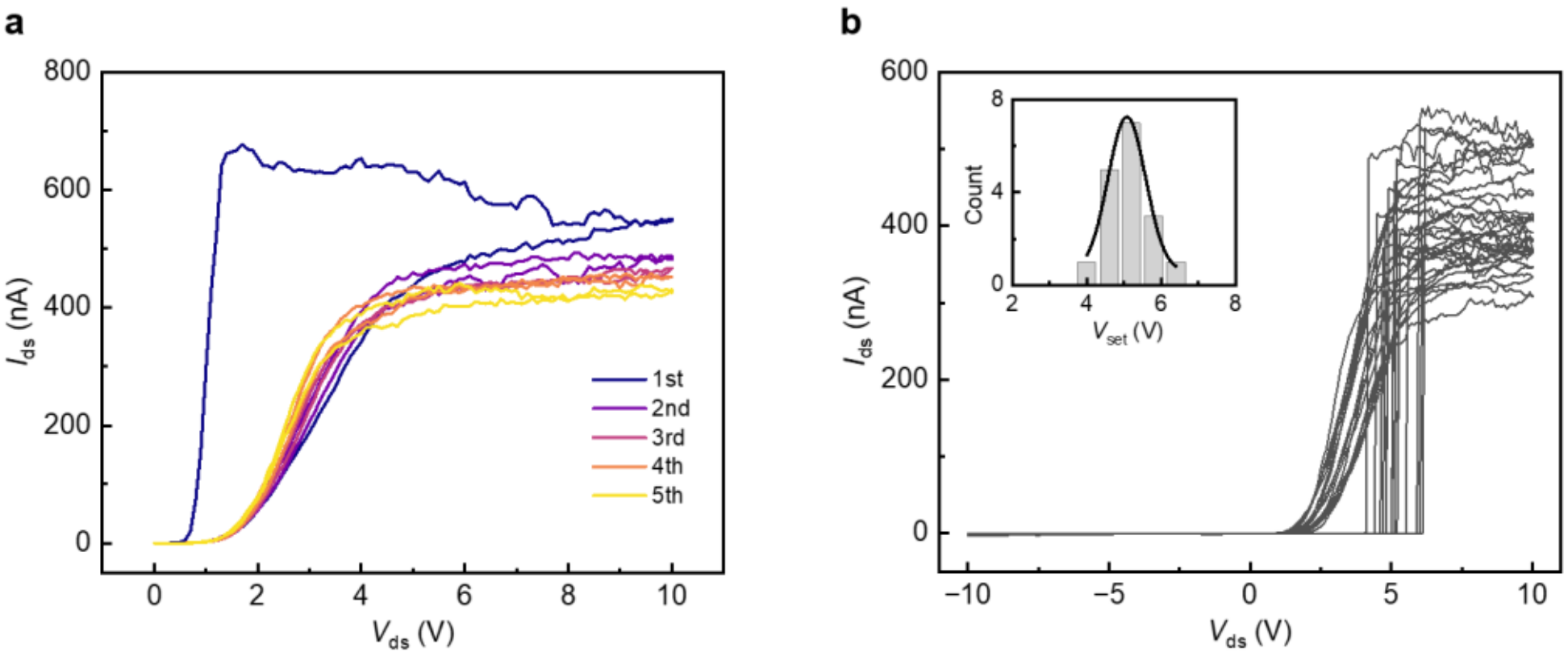

Supplementary Fig. 8 | Repeated *I*–*V* sweeps of device D1 at 2 K, with the bias restricted to positive values following a single pulsed light illumination (**a**), and with identical optical resets before each measurement (**b**). The switching threshold consistently appears near 5 V. Inset: Statistical distribution of threshold voltages, well described by a Gaussian centered at 5 V with a standard deviation of ~1.2 V. The repeated measurements under identical conditions are used to quantitatively evaluate the reproducibility and statistical variation of V_{set}.

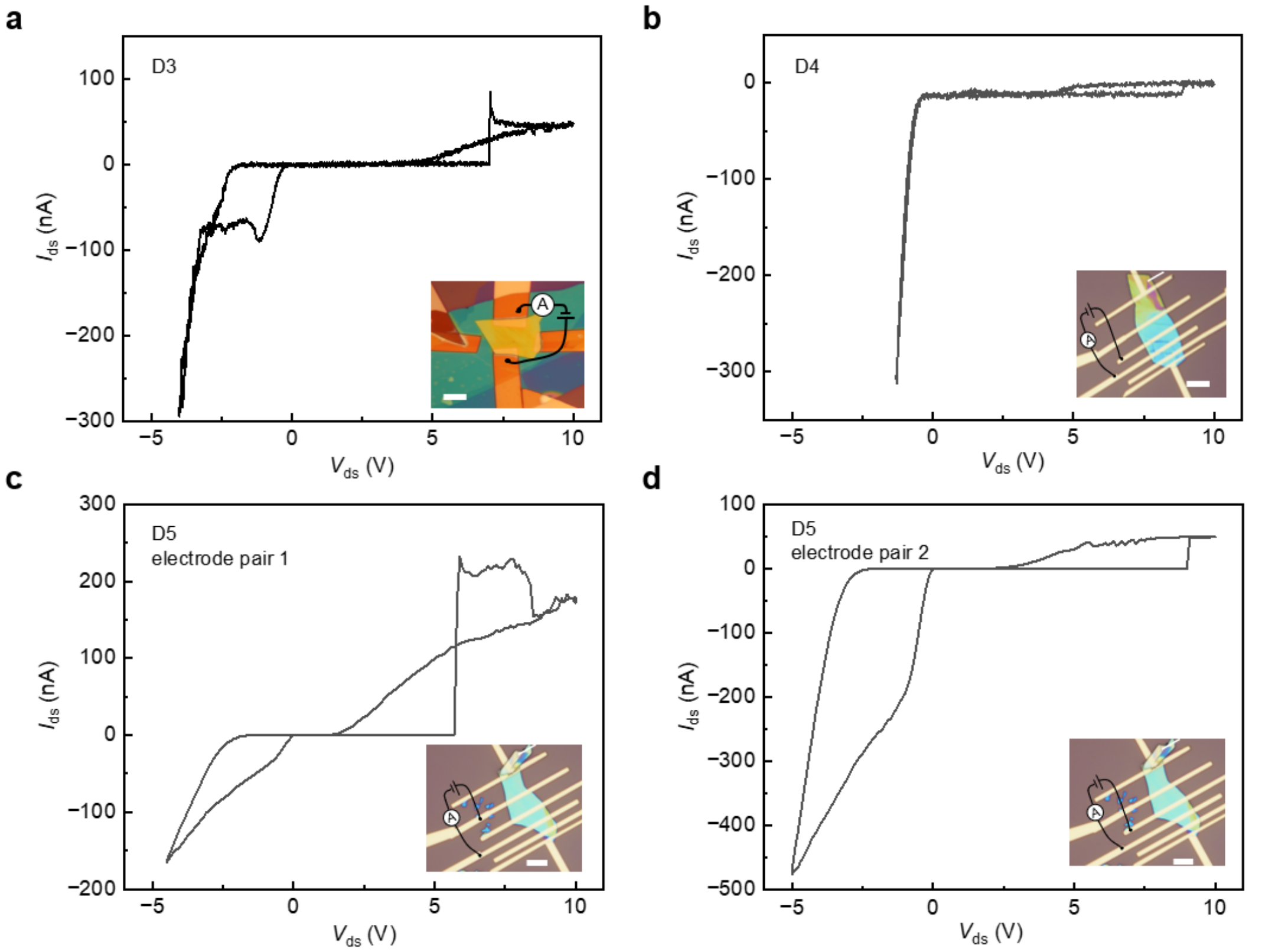

Supplementary Fig. 9 | Photo-activated digital resistive switching in devices D3-D5. Measurements presented in panels (c) and (d) were performed on different pairs of electrodes of the same device. Scale bars: 5 µm. Variations in V_{set}, on/off ratio and overall conductivity are observed across devices, which are attributed to differences in contact quality, intrinsic defect density, and device dimensions.

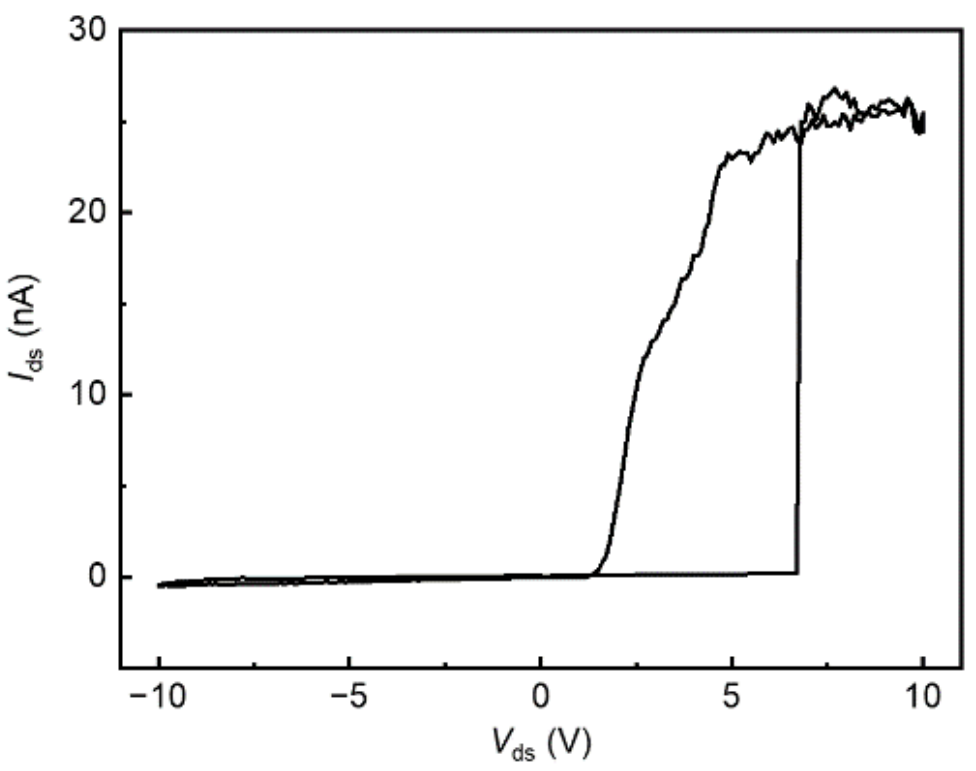

Supplementary Fig. 10 | *I–V* characteristics of device D1 measured 12 hours after the application of a light pulse. Digital resistive switching behavior persist, although the LRS current is significantly reduced.

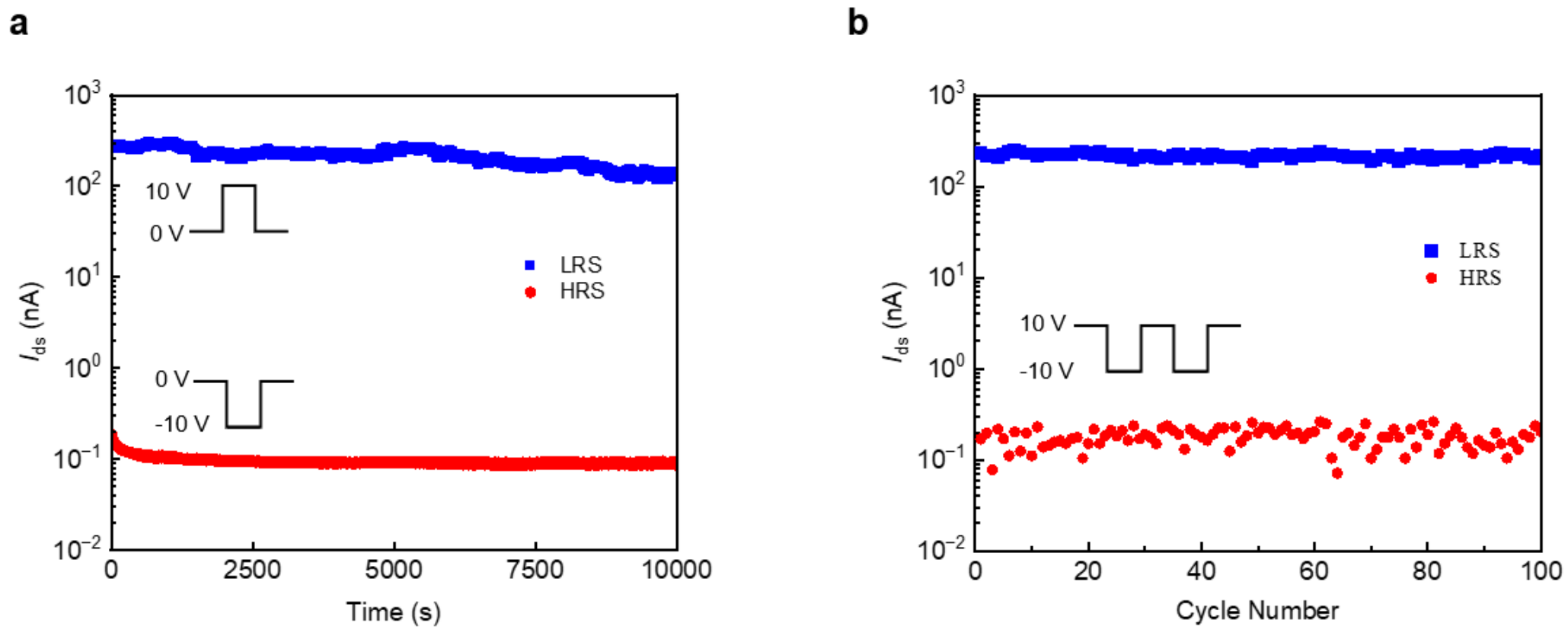

Supplementary Fig. 11 | Retention (a) and endurance (b) tests of device D1 at 2 K. The readout voltage is 4 V. The durations of the set (10 V) and reset (-10 V) electrical pulses are both 1 ms.

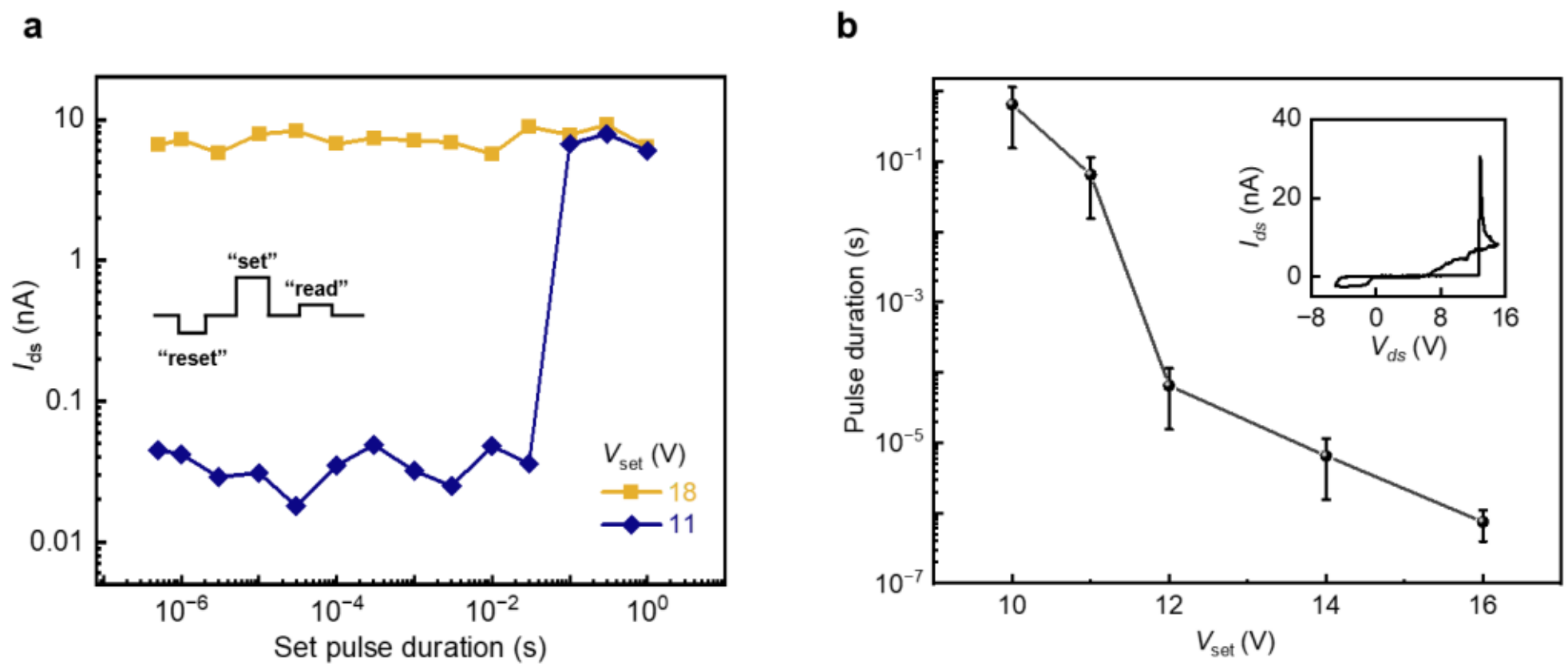

Supplementary Fig. 12 | Switching-speed measurements of α-In_2Se_3 (device D6). **a**, Read current (at V_{read} = 8 V) after representative set pulses (11 V and 18 V) of different durations. Prior to the set pulse, a reset pulse of -9 V, 1 s was applied. **b**, Critical set pulse duration as a function of V_{set}. The critical set pulse duration is identified where the device switches from high- to low-resistance states. Inset: the *I*-*V* sweep performed after the pulsed light illumination (0.14 mW cm^{-2}, 5 s).

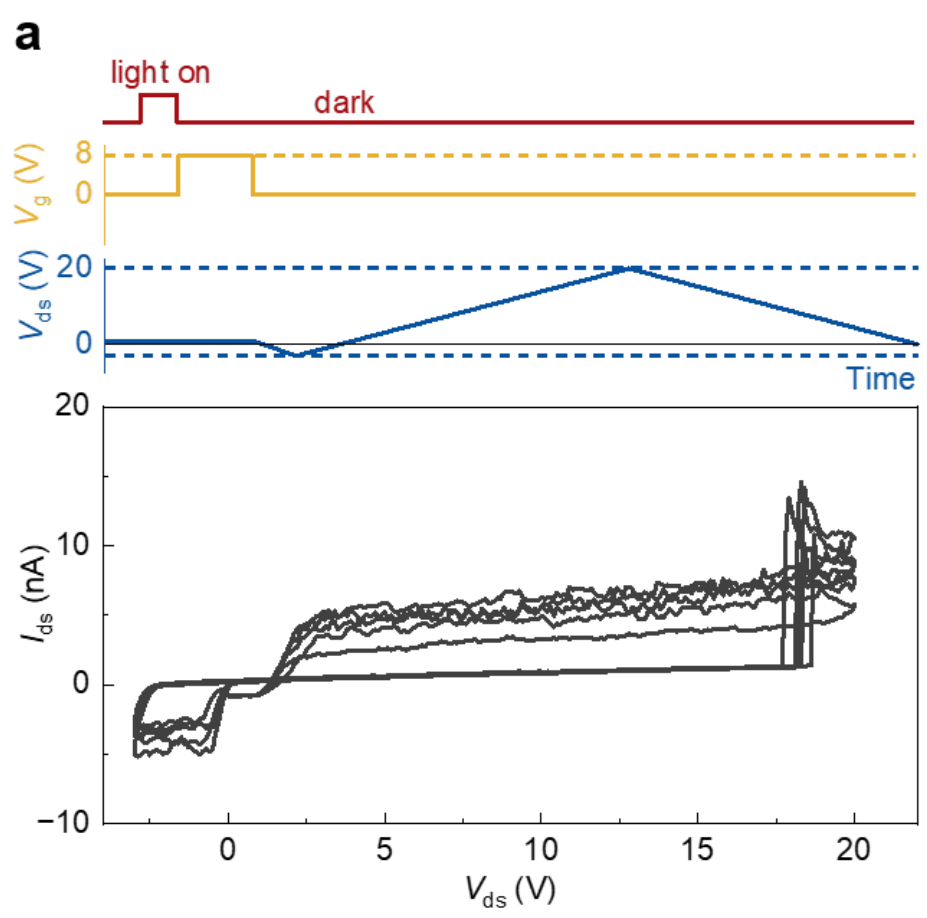

Supplementary Fig. 13 | Effect of gate voltage pulses on the resistive switching. After the illumination, a gate voltage pulse of 8 V (a) or -8 V (b) is applied for 10 seconds, followed by a V_{ds} scan in the sequence: 0 V, -3 V, 20 V, 0 V, under V_g = 0 V. The obtained V_{set} is found to fall within the standard deviation. This demonstrates the effect of V_g is volatile.

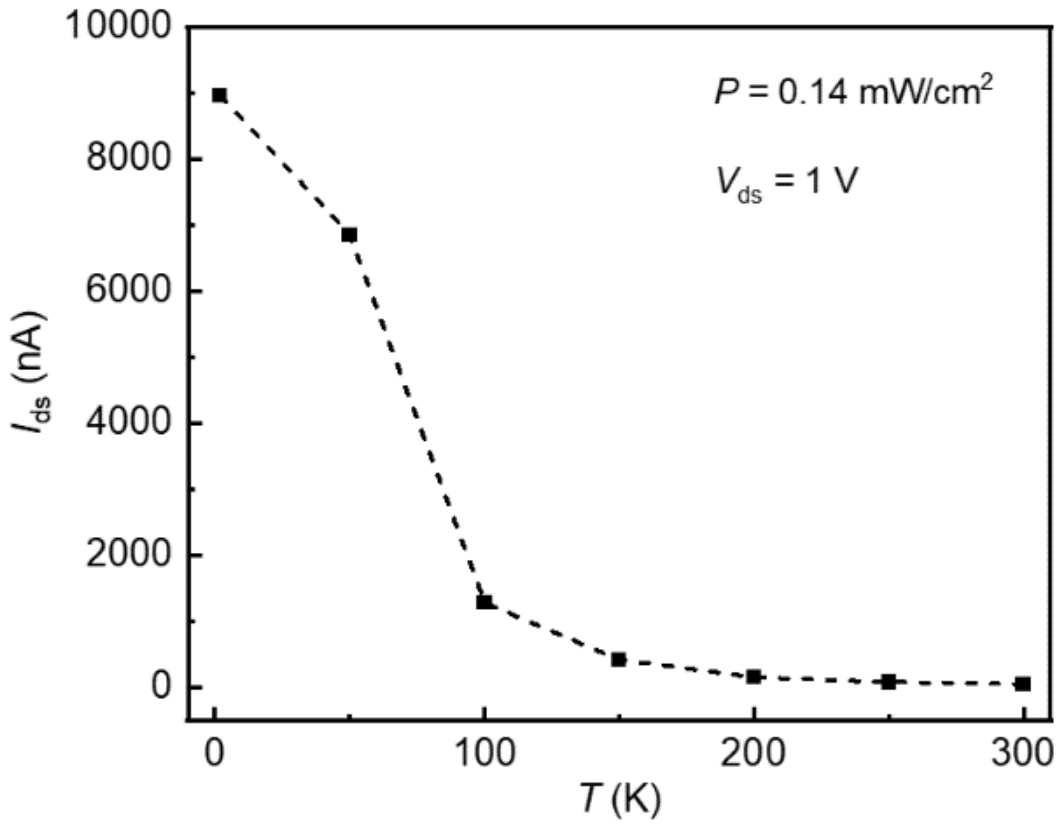

Supplementary Fig. 14 | Temperature-dependent photocurrent under continuous illumination.

Measurements were performed at an optical intensity of 0.14 mW cm^{-2} with V_{ds} = 1 V.

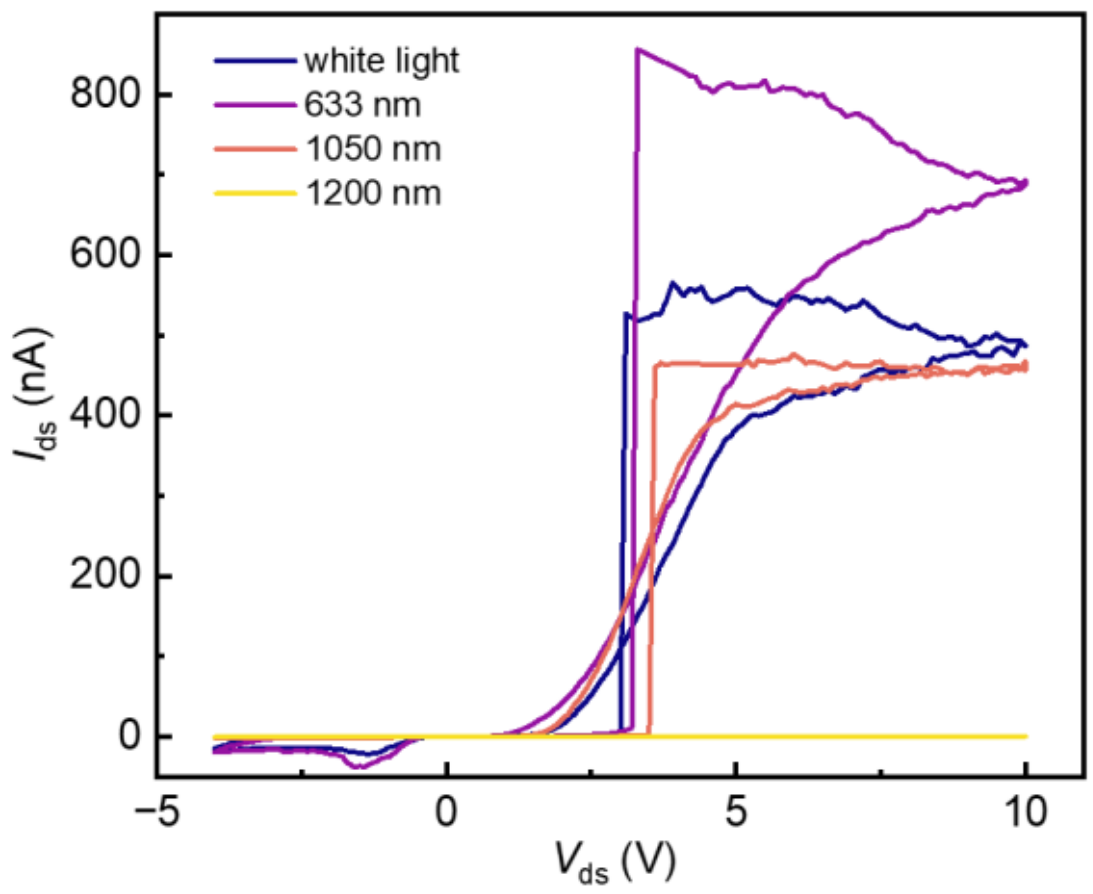

Supplementary Fig. 15 | Wavelength dependence of photo-activated resistive switching. *I*-*V* sweeps (0 V, -4 V, 10 V, 0 V) following illumination by different pulsed lights at V_{ds} = 1 V. No signature of resistive switching is observed after illumination with 1200 nm light.

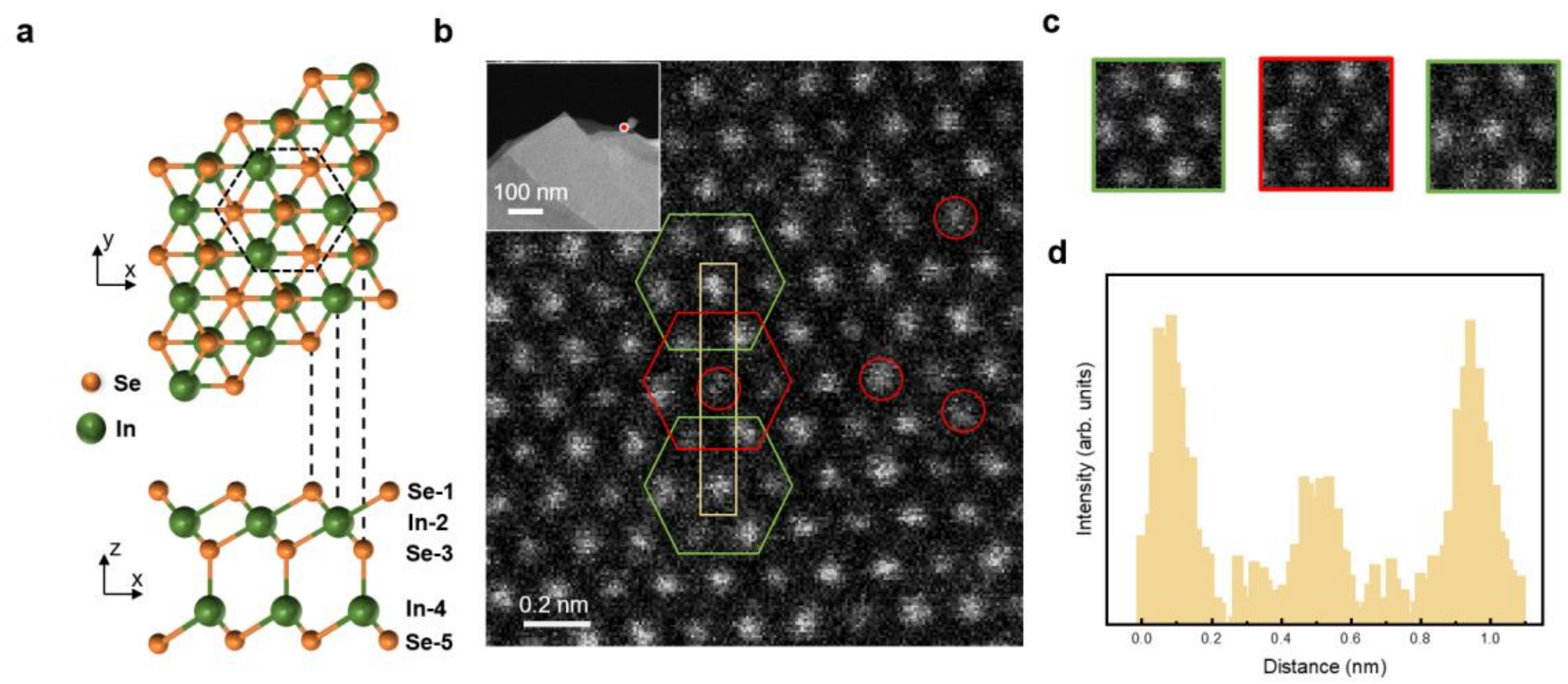

Supplementary Fig. 16 |Structural analysis of α-In_2Se_3 thin flakes. a, Schematic crystal structure of α-In_2Se_3 in top and side views. **b,** Atomic-resolution top-view STEM image of an α-In_2Se_3 thin flake. The red and green polygons outline representative defective and pristine unit cells, respectively, and the red circles indicate the positions of the selenium vacancy ($V_{Se\text{-}3}$). The inset shows the image of the flake, with the red dot indicating the location where the STEM image was acquired. **c,** Representative pristine and defective regions extracted from **b** for comparison. **d,** Line profile of the STEM intensity extracted along the yellow box in **b**, the peak intensity at the center of the profile is approximately half of the at the two neighboring sites, consistent with the presence of $V_{Se\text{-}3}$ at the center of the box.

a

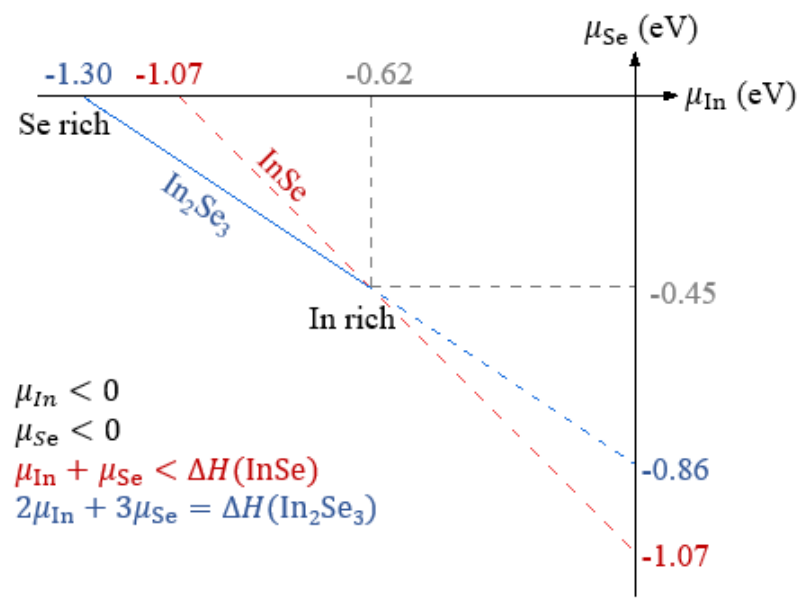

b

Formation Energy (eV)	In rich	Se rich
$V_{Se\text{-}1}$	1.73	2.18
$V_{In\text{-}2}$	2.78	2.10
$V_{Se\text{-}3}$	0.25	0.70
$V_{In\text{-}4}$	3.72	3.04
$V_{Se\text{-}5}$	0.84	1.29

Supplementary Fig. 17 | Formation energies of different vacancy defects in α-In_2Se_3. a, Chemical potential phase diagram defining the thermodynamically allowed In-rich and Se-rich growth conditions for α-In_2Se_3. **b,** Calculated formation energies of different vacancy defects under the In-rich and Se-rich limits.

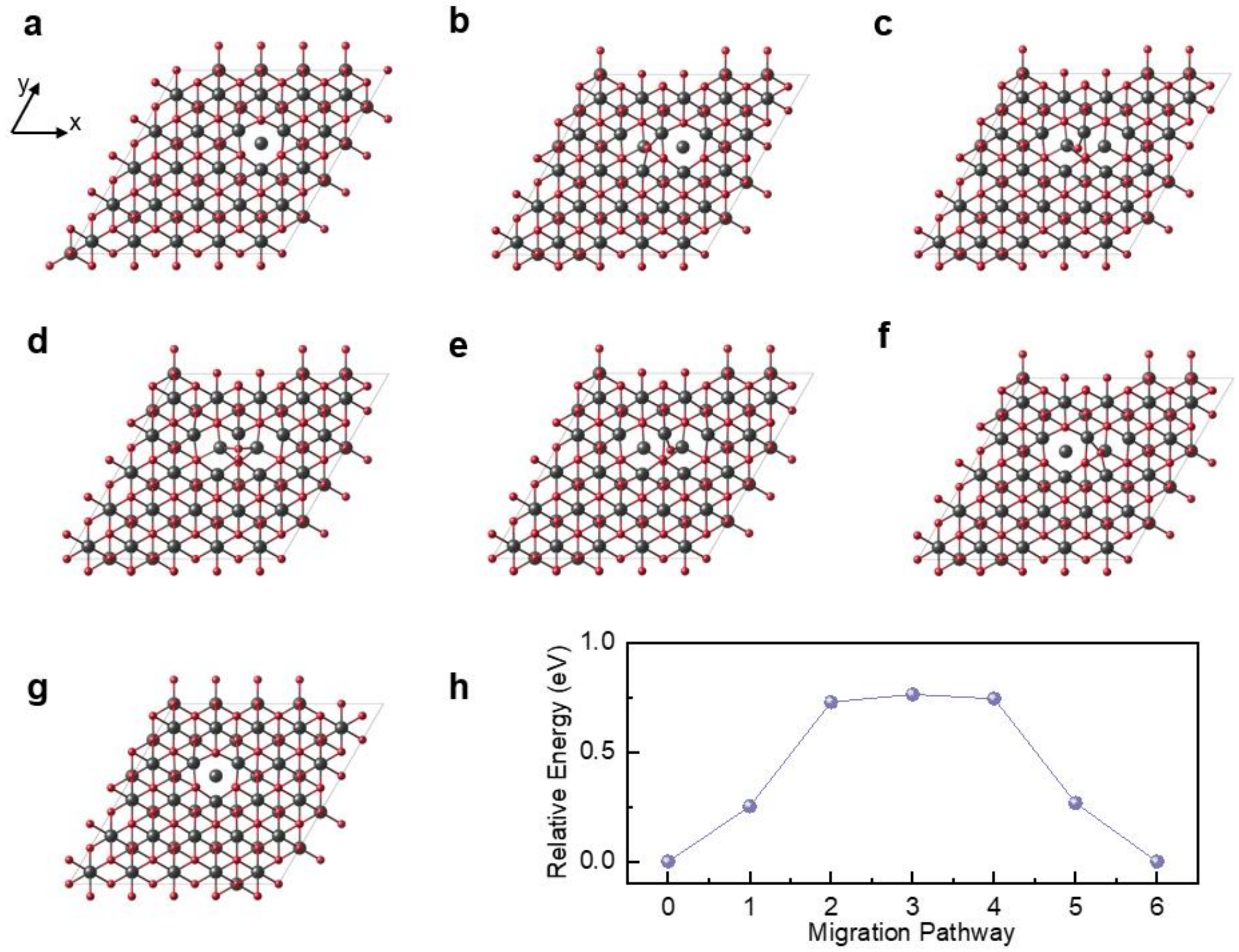

Supplementary Fig. 18 | Simulation of the migration barrier for a single $V_{Se\text{-}3}$ defect. a-g, Atomic configurations along the migration pathway of a $V_{Se\text{-}3}$ defect. **h,** The calculated relative energy profile corresponding to the migration pathway shown in **a-g**.

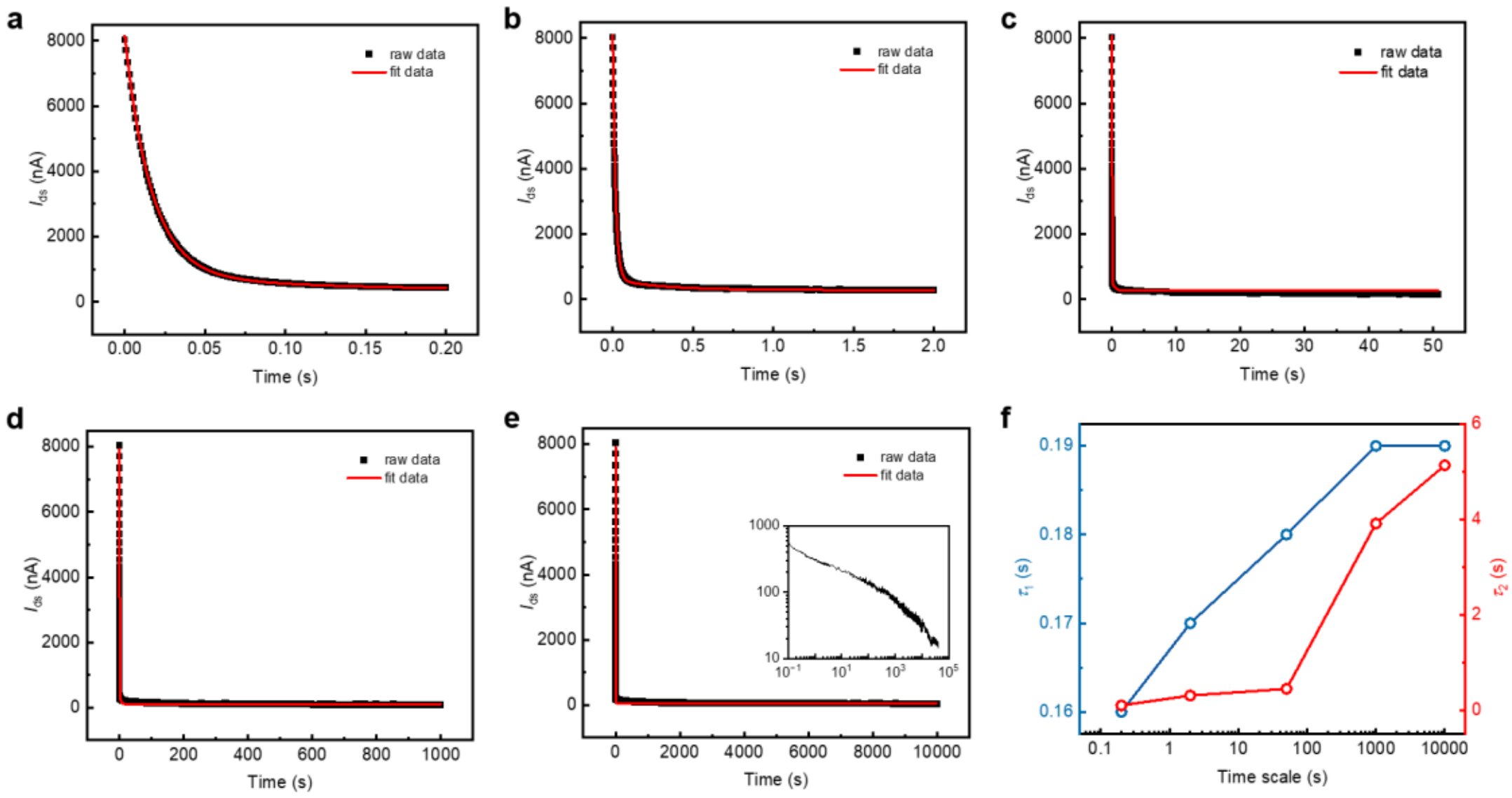

Supplementary Fig. 19 | Analysis of the transient photoresponse decay. The photocurrent decay was fitted using a double-exponential function, $I(\mathrm{t}) = C_0 + C_1 \exp(-t/\tau_1) + C_2 \exp(-t/\tau_2)$, at selected time windows after illumination: 0.2 s (**a**), 2 s (**b**), 50 s (**c**), 1000 s (**d**), and 10,000 s (**e**). The extracted time constants τ_1 and τ_2 as a function of time window are plot in (**f**), indicating strong dependence on the chosen time range and suggesting that the relaxation dynamics cannot be fully described by a simple double exponential decay. Moreover, the log-log plot in the inset of (**e**) further suggests that the decay may instead follow a double-power law behavior over a long time period.

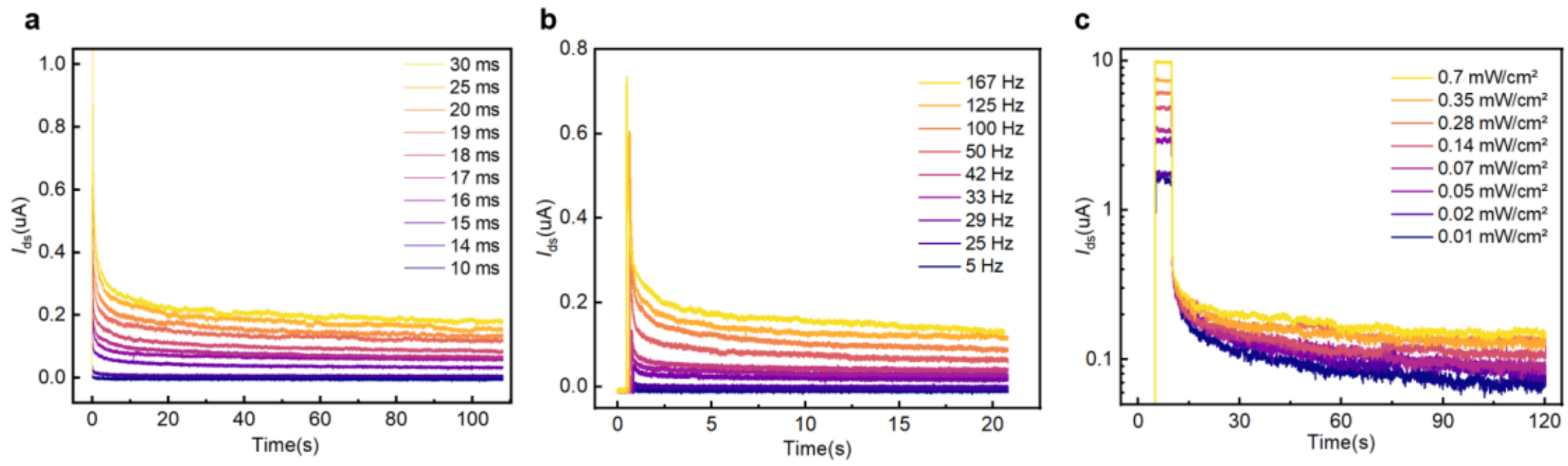

Supplementary Fig. 20 | The multilevel conductance states at V_{ds} = 1 V obtained by other programming strategies. a, Spike-timing-dependent plasticity (STDP): 2 ms light pulses at 0.05 mW cm^{-2} with varying time intervals. **b**, Spike-frequency-dependent plasticity (SFDP): trains of 10 optical pulses, each with a duration of 2 ms and an intensity of 0.05 mW cm^{-2}, at varying frequencies. **c**, Spike-amplitude-dependent (SAD) plasticity: 5 s light pulses with different intensities.

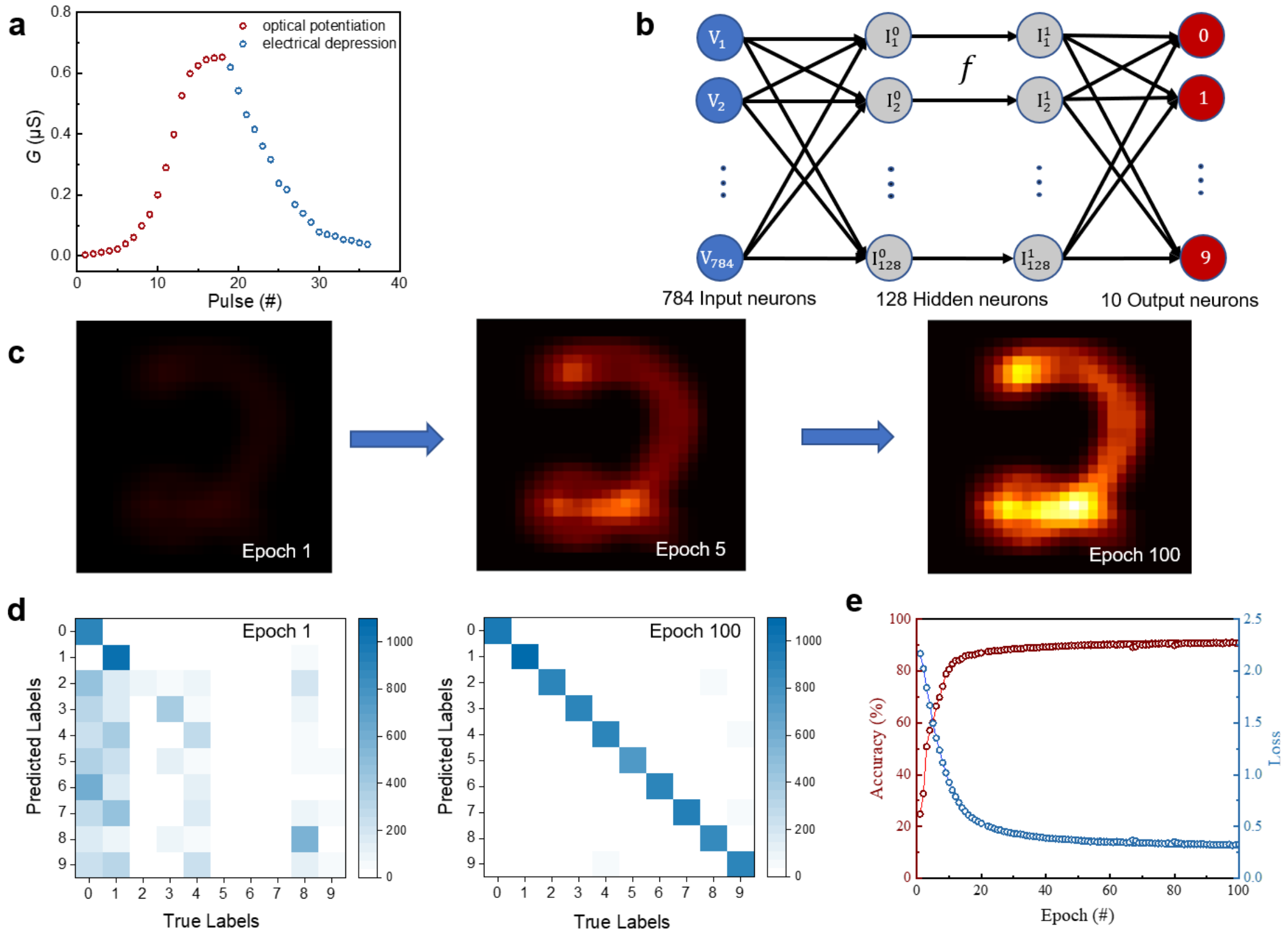

Supplementary Fig. 21 | Multilevel conductance states and handwritten digit recognition using an artificial neural network (ANN). a, Optical potentiation and electrical depression tests of the device with optical and electrical pulses, displaying 36 distinct conductance levels (V_{read} = 2 V). These experimentally extracted conductance values serve as synaptic weights in the ANN simulation shown in **b-e**. **b**, Schematic of our ANN structure. The input image is decomposed into a 28×28 data matrix and flattened into a 784×1 vector (input layer). This vector is then processed through summation via a weight matrix to produce 128 intermediate variables, followed by transformation using a Rectified Linear Unit (ReLU) activation function (hidden layer). Finally, the output is mapped to digits 0 through 9 (output layer), corresponding to the classification of the input image. The entire network was trained iteratively. **c**, Reconstructed images of the digit "2" after different training epochs. **d**, Confusion matrix after the 1st and 100th training epoch. **e**, Recognition accuracy and loss as a function of the training epoch. With increasing epoch number, the recognition accuracy steadily improves while the loss decreases, ultimately reaching ~91% and ~0.32, respectively, after 100 epochs. The simulation serves as a proof-of-principle demonstration that the observed multilevel conductance states are sufficient for neuromorphic image recognition, rather than a full hardware validation.

Supplementary Table 1 | Comparison of key metrics of memristors operating at cryogenic temperatures.

Structure	Temperature	Switching speed	On/off ratio	Retention time	Reference
ITO/HfOx/ITO	10 K	NA	100	10^6 s	[4]
Pt/HfOx/TiN	4 K	NA	100	NA	[5]
Perovskite (La, Pr, Ca)MnO_3	2 K	1 ms	10^5	NA	[6]
Ge/GeSi	25 K	NA	10^3	10^4 s	[7]
HZO	10 K	≳900 ns	NA	10^4 s	[8]
W/AlO_x/Al_2O_3/Pt	100 K	28 ns	10^3	10^6 s	[9]
Al_2O_3/TiO_{2-x}	4.2 K	200 ns	2	over a month	[10]
α-In_2Se_3	2 K	<500 ns	10^3	10^4 s	This work

Supplementary Table 2 | Comparison of responsivity and specific detectivity of α-In_2Se_3-based photodetectors.

Structure	Geometry	Wavelength (nm)	Bias(V)	R(A/W)	D*(Jones)	Reference
α-In_2Se_3 nanowire	lateral	500	3	89	NA	[11]
α-In_2Se_3 nanosheet	lateral	300	5	3.95×10^3	2.26×10^{12}	[12]
α-In_2Se_3	lateral	640	0.05	$\sim 10^5$	3×10^{13}	[13]
2H α-In_2Se_3	lateral	700	0.2	1081.5	$\sim 1.03 \times 10^{11}$	[14]
α-In_2Se_3 nanosheet	lateral	850	0.5	3.43	NA	[15]
2H α-In_2Se_3	lateral	650	1	75	1.46×10^{10}	[16]
2H α-In_2Se_3	lateral	400	1	1936	2.1×10^{13}	[17]
2H α-In_2Se_3	lateral	447	1	42.9	1×10^{12}	[18]
2H α-In_2Se_3	lateral	808	1	720	2.2×10^{12}	[19]
ML α-In_2Se_3	lateral	532	1	340	NA	[20]
3R α-In_2Se_3	lateral	750	-10	34500	NA	[21]
2H α-In_2Se_3	lateral	White LED	1	167	1.26×10^{13}	This work
2H α-In_2Se_3 (2 K)	lateral	White LED	1	110000	1.8×10^{16}	This work
3R α-In_2Se_3	vertical	680	0.5	1000	$>10^{13}$	[22]
2H α-In_2Se_3	vertical	520	1.5	4.3×10^4	2.3×10^{14}	[23]

Supplementary Table 3 | Comparison of key metrics of representative cryogenic photomemory systems.

Structure	Optical retention	On/off ratio	Optical conditions	Temperature	Reference
MoS_2 FET	30 days	10^2	29.6 mW cm^{-2}, 5 min, 365 nm laser	6 K	[24]
GaAsN	4000 s	5	700 s, 945 nm LED	77 K	[25]
GeSbTe	10^4 s	1.5	1 mW, 3 s, 880 nm laser	4.1 K	[26]
Sb_2Se_3	Hours	~10^5	10 mW cm^{-2}, 1 min, 520 nm	22 K	[27]
GaP	3000 s	NA	10 mW, 200 s, White light	50 K	[28]
SnO_2	6 s	1.3	60 µW mm^{-2}, 10 s, 405 nm laser	9 K	[29]
$ZnO_{1-\delta}$	152.5 s	10^3	485 µW cm^{-2}, 600 s, UV illumination	11 K	[30]
$Cd_{0.97}Mn_{0.03}Te$	Hours	10^4	10^{16} photons cm^{-2} s^{-1}, 30 min, White light	40 K	[31]
β-Ga_2O_3	4×10^4 s	10^2	1 h, 600 nm laser	90 K	[32]
WS_2 FET	~5000 s	~500	0.5 mW, 20 min, 462 nm laser	20 K	[33]
Tl_6SeI_4	2 hours	1.6×10^4	2000 mW cm^{-2}, 60 s, 405 nm laser	104 K	[34]
α-In_2Se_3 FET	> 10^3 s	> 10^3	0.14 mW cm^{-2}, 5 s, White light	2 K	This work

Supplementary Note 1. Evaluation of the photoresponsivity and specific detectivity

The optical power density at the sample plane was determined to be 0.14 mW cm^{-2} using a calibrated photodiode power meter with an active sensing area of 70.88 mm^2. Device D1 has a channel length of ~5.5 μm and a width of ~11 μm, yielding an optical exposure area of ~60 μm^2. Accordingly, the incident optical power on the device is 9×10^{-8} mW. The device's photoresponsivity $R = I_{ph}/P$ at V_{ds} = 1 V is calculated to be 167 and 1.1×10^5 A W^{-1} at 300 K and 2 K, respectively.

The specific detectivity (D^*) is defined as $D^* = \sqrt{A\Delta f}/\mathrm{NEP}$, where A is the photosensitive area of the device, Δf is the measurement bandwidth and NEP represents the Noise Equivalent Power. The NEP can be expressed as NEP = I_n/R, where I_n denotes the noise current of the dark current (I_d). Several mechanisms contribution to I_n, including shot noise, the Johnson-Nyquist noise and 1/f noise. For device D1, the shot noise ($I_{\mathrm{shot}} = \sqrt{2qI_{\mathrm{d}}\Delta f}$) dominates over the Johnson-Nyquist noise ($I_{JN} = \sqrt{4k_B T\Delta f/R}$). Using this relation, we estimate the specific detectivity of device D1 to be 1.3×10^{13} and 1.8×10^{16} Jones at 300 K and 2 K, respectively.

Supplementary References:

1. Liu, L. X. et al. Atomically Resolving Polymorphs and Crystal Structures of In_2Se_3. *Chem. Mater.* **31**, 10143–10149 (2019).
2. Wu, Y. et al. Stacking selected polarization switching and phase transition in vdW ferroelectric α-In_2Se_3 junction devices. *Nat. Commun.* **15**, 10481 (2024).
3. Si, M. W. et al. A ferroelectric semiconductor field-effect transistor. *Nat. Electron.* **2**, 580–586 (2019).
4. Shang, J. et al. Thermally Stable Transparent Resistive Random Access Memory based on All-Oxide Heterostructures. *Adv. Funct. Mater.* **24**, 2171–2179 (2014).
5. Fang, R., Chen, W., Gao, L., Yu, W. & Yu, S. Low-Temperature Characteristics of HfO_x-Based Resistive Random Access Memory. *IEEE Electron Device Lett.* **36**, 567–569 (2015).
6. Yi, H. T., Choi, T. & Cheong, S. W. Reversible colossal resistance switching in (La,Pr,Ca)MnO_3: Cryogenic nonvolatile memories. *Appl. Phys. Lett.* **95**, 063509 (2009).
7. Bradicich, A. & Lu, T.-M. Programmable Cryogenic Memory in a Ge/GeSi Heterostructure. *Adv. Electron. Mater.* **11**, e00102 (2025).
8. Das, A. et al. Sub-2 nm Equivalent-Oxide-Thickness Ferroelectric Transistors for Cryogenic Memory and Computing. *ACS Nano* **20**, 10905–10918 (2026).
9. Huang, X. D. et al. Forming-Free, Fast, Uniform, and High Endurance Resistive Switching From Cryogenic to High Temperatures in W/AlO_x/Al_2O_3/Pt Bilayer Memristor. *IEEE Electron Device Lett.* **41**, 549–552 (2020).
10. Mouny, P.-A. et al. Analog programming of CMOS-compatible Al_2O_3/TiO_{2-x} memristor at 4.2 K after metal-insulator transition suppression by cryogenic reforming. *Appl. Phys. Lett.* **123**, 163505 (2023).
11. Zhai, T. et al. Fabrication of High-Quality In_2Se_3 Nanowire Arrays toward High-Performance Visible-Light Photodetectors. *ACS Nano* **4**, 1596–1602 (2010).
12. Jacobs-Gedrim, R. B. et al. Extraordinary Photoresponse in Two-Dimensional In_2Se_3 Nanosheets. *ACS Nano* **8**, 514–521 (2014).
13. Island, J. O., Blanter, S. I., Buscema, M., van der Zant, H. S. & Castellanos-Gomez, A. Gate Controlled Photocurrent Generation Mechanisms in High-Gain In_2Se_3 Phototransistors. *Nano Lett.* **15**, 7853–7858 (2015).
14. Tang, B. et al. UV-SWIR broad range photodetectors made from few-layer α-In_2Se_3 nanosheets. *Nanoscale* **11**, 12817–12828 (2019).
15. Li, X. et al. Multi-Functional Platform for In-Memory Computing And Sensing Based on 2D Ferroelectric Semiconductor α-In_2Se_3. *Adv. Funct. Mater.* **34**, 2306486 (2024).
16. Liu, T. et al. Ferroelectric Tunable Nonvolatile Polarization Detection Based on 2H α-In_2Se_3. *ACS Appl. Mater. Interfaces* **17**, 18592–18600 (2025).
17. Wang, S. et al. Strong Anisotropic Two-Dimensional In_2Se_3 for Light Intensity and Polarization Dual-Mode High-Performance Detection. *ACS Appl. Mater. Interfaces* **15**,

3357–3364 (2023).

18. Zhang, W., Su, Q., Zhang, B., Peng, J. & Li, Y. α-In_2Se_3 Nanostructure-Based Photodetectors for Tunable and Broadband Response. *ACS Appl. Nano Mater.* **6**, 8795–8803 (2023).
19. Anandan, M. et al. High-responsivity broad-band sensing and photoconduction mechanism in direct-Gap α-In_2Se_3 nanosheet photodetectors. *Nanotechnology* **31**, 465201 (2020).
20. Zhou, J. et al. Controlled Synthesis of High-Quality Monolayered α-In_2Se_3 via Physical Vapor Deposition. *Nano Lett.* **15**, 6400–6405 (2015).
21. Seo, D. H. et al. Characteristics of Rhombohedral (3R) Structure of α-In_2Se_3 Nanosheets by Mechanical Exfoliation. *Electron. Mater. Lett.* **20**, 192–198 (2024).
22. Mech, R. K. et al. High Responsivity and Photovoltaic Effect Based on Vertical Transport in Multilayer α-In_2Se_3. *physica status solidi (a)* **217**, 1900932 (2020).
23. Liu, X. et al. Two-dimensional α-In_2Se_3-based ferroelectric semiconductor junction for reconfigurable photodetectors. *Appl. Phys. Lett.* **122** (2023).
24. George, A. et al. Giant persistent photoconductivity in monolayer MoS_2 field-effect transistors. *npj 2D Mater. Appl.* **5**, 15 (2021).
25. Field, R. L. et al. Influence of N incorporation on persistent photoconductivity in GaAsN alloys. *Phys. Rev. B* **87**, 155303 (2013).
26. Ovadyahu, Z. Coexistence of electron-glass phase and persistent photoconductivity in GeSbTe compounds. *Phys. Rev. B* **91**, 094204 (2015).
27. Herklotz, F. et al. Persistent photoconductivty in n-type Sb_2Se_3. *Phys. Rev. Mater.* **9**, 084604 (2025).
28. Hartnett, J. G. et al. Observation of persistent photoconductivity in bulk gallium arsenide and gallium phosphide samples at cryogenic temperatures using the whispering gallery mode method. *J. Appl. Phys.* **104**, 113714 (2008).
29. Martinez-Gazoni, R. F., Allen, M. W. & Reeves, R. J. Persistent Photoconductivity in SnO2 Thin Films Grown by Molecular Beam Epitaxy: The Dominant Roles of Water Vapor and Carrier Concentration. *J. Phys. Chem. C* **125**, 26967–26977 (2021).
30. Mondal, S. & Basak, D. Temperature-dependent photoconductivity as a tool to unveil defect-mediated carrier dynamics in $ZnO_{1-\delta}$ thin films. *Opt. Mater.* **171**, 117756 (2026).
31. Semaltianos, N. G., Karczewski, G., Wojtowicz, T. & Furdyna, J. K. Persistent photoconductivity and photoionization of deep electron traps in Ga-doped $Cd_{1-x}Mn_xTe$. *Phys. Rev. B* **47**, 12540–12549 (1993).
32. Traoré, A., Okumura, H. & Sakurai, T. Photoconductivity buildup and decay kinetics in unintentionally doped β-Ga_2O_3. *Jpn. J. Appl. Phys.* **61**, 091002 (2022).
33. Qin, M. et al. Light Controllable Electronic Phase Transition in Ionic Liquid Gated Monolayer Transition Metal Dichalcogenides. *Nano Lett.* **21**, 6800–6806 (2021).
34. Das, S. et al. Charge Transport and Observation of Persistent Photoconductivity in Tl_6SeI_4

Single Crystals. *J. Phys. Chem. Lett.* **8**, 1538–1544 (2017).
