# Supplementary material for: Photo-activated digital resistive switching in a cryogenic photomemory
Source: Nat Commun. 2026 Jul 28;17:9167. doi: 10.1038/s41467-026-76121-2 (PMC13522526; doi:10.1038/s41467-026-76121-2)
Supplement: Supplementary file 2 [file 41467_2026_76121_MOESM2_ESM.pdf]

nature portfolio

Peer Review File

Photo-activated digital resistive switching in a cryogenic photomemory

Corresponding Author: Professor Zaiyao Fei

This file contains all reviewer reports in order by version, followed by all author rebuttals in order by version.

Version 1:

Reviewer comments:

Reviewer #1

(Remarks to the Author)
The manuscript reports intriguing measurements of non-volatile resistance changes in three terminal In_2Se_3 devices as a function of drain-source bias, temperature, gate bias, and illumination. The central novel finding is a well-defined threshold for resistive switching under positive drain source bias (negative biases can reset the resistance) that is largest at low temperatures and can be controlled by the intensity and duration of illumination with visible and near-IR light. The threshold can also be tuned by the gate voltage, where majority carrier accumulation reduces the threshold. The underlying kinetics of the switching process support recognized programming schemes such as paired-pulse facilitation, albeit at rather low frequencies.

In Figure 3, the manuscript provides a possible explanation for the observed resistive switching. Specifically, it is attributed to the movement of positively charged selenium vacancies adjacent to electrodes such that the depletion region associated with the Schottky barrier contacts can be bridged by conducting filaments. This explanation is reasonable, but speculative; there is no direct experimental evidence for the accumulation of vacancies, nor for the potential drop (and hence resistance modulation) arising from (one of?) the contacts. Furthermore, given the reported polarization switching in Fig. 1c, which occurs at biases below those used in subsequent device measurements, the following statement on page 6 raises creates some confusion: “The photo-activated digital resistive switching we observe could, in principle, originate from two distinct microscopic processes: (i) a defect-mediated reorganization that creates localized conductive channels, or (ii) a defect-facilitated reversal of ferroelectric domains that drastically lowers the contact barrier. Critical experimental observations, however, strongly favor the former.” The manuscript provides direct evidence for polarization reversal but not vacancy accumulation. Are both perhaps present? If so, how do they interact?

Overall, the studies are carefully done, the effects are clear, and the claims are appropriately qualified. The manuscript is of high quality with results that are of broad interest. That said, the potential impact on device applications is unclear. Rather high biases are applied for rather long times to achieve the behaviors of interest, and without clear evidence for the underlying switching mechanism, it is not possible to assess whether voltage thresholds and switching times may be reduced into practical regimes.

Reviewer #2

(Remarks to the Author)
Recommendation: Minor revision

The manuscript by Wang et al. reports the discovery of a photo-activated digital resistive switching effect in α In_2Se_3 lateral devices operating at cryogenic temperatures. The authors demonstrate that a single optical pulse enables digital resistive switching between high- and low-resistance states, with threshold voltages tunable via electrical reset pulses and electrostatic gating. The same defect-mediated mechanism also gives rise to a giant photoresponse, electrically resettable persistent photoconductivity, and cryogenic synaptic plasticity. The work is novel, technically sound, and of potential interest to the broad readership of Nature Communications. However, several issues remain that should be addressed before publication.

1. Regarding the microscopic mechanism, the authors favor a conductive channel formation mechanism over ferroelectric switching. However, direct imaging of the conductive filament (e.g. via in-situ TEM or conductive AFM) would make the arguments more convincing. Please comment on the feasibility of such experiments and, if not feasible, clarify what indirect evidence most strongly supports their model.

2. Multiple capabilities have been demonstrated in the same device (cryogenic operation, optical activation, gate tuning, binary wavelength selectivity), and several applications (reconfigurable cryogenic memory, photodetector, neuromorphic functionalities) are mentioned without clearly prioritizing the most defensible one. The authors should identify and justify the most unique and compelling application for this particular device?

3. The authors reproduced the cryogenic photo-activated digital resistive switching behavior across multiple α- In2Se3 devices. However, key parameters such as Vset, on/off ratio and conductivity at negative bias appear to vary among these devices. What makes the differences?

4. The authors state that in the dark, V_{set} is inaccessible. Have the authors attempted applying higher bias voltages to determine whether switching can be triggered in the dark? If not, what limits the bias range?

5. In Fig. 2e, additional illumination under negative bias can effectively suppress the device's switching capability. Is this observation consistent with their microscopic model?

6. The authors apply a bias voltage of 1 V during the initial illumination stage. Is there a specific reason for this choice? Does the photo-activation also occur at other bias voltages, or even in the absence of bias?

7.The authors cite Ref 29 to explain the "crossing point" in I-V loops as a competition between ferroelectric switching and defect migration. Ref 29 refers to β'-In2Se3, whereas this work focuses on the α-phase. While the mechanisms are related, the structural differences (e.g., the interlocking of in-plane and out-of-plane polarization in α-phase) may lead to different migration barriers.

8. The manuscript contains typographic errors, e.g. "defectscal" in the abstract. Please carefully proofread the entire text.

Reviewer #3

(Remarks to the Author)
The authors report photo-activated resistive switching in In2Se3 at 2 K. While the physical concept is interesting, the manuscript lacks direct mechanistic evidence and falls short on practical memory metrics. I recommend major revisions based on the following points:

1. The mechanism of the proposed microscopic model inferred solely from I-V curves. Please provide direct microstructural evidence (e.g., TEM images) or DFT calculations.

2. It would be beneficial to experimentally measure the switching speed of the device and discuss the results.

3. Supplementary Fig. 17 illustrates a simulated application of the device (ANN training and reasoning). The performance is measured at 2k. But the real ANN training and reasoning could not be performed at 2k. It differs significantly from practical scenarios.

4. The device is compared only with other In_2Se_3-based works, which is insufficient. It is suggested adding a benchmarking table that compares the device with state-of-the-art photomemory devices at cryogenic temperatures across key metrics.

5. Please clarify the purpose of performing Gaussian fitting on the switching threshold.

6. On page 1, line 29, "defectscal pulses" appears to be a typo. Please correct it.

Version 2:

Reviewer comments:

Reviewer #1

(Remarks to the Author)
The authors have addressed the points of confusion raised in my review. While the cryogenic resistive switching mechanism(s) is(are) not identified unambiguously, the additional experiments and calculations provide support for the involvement of photo-activated vacancies. The biases required for switching are still impractically large, but the revised manuscript at provides additional experiments showing that switching time scales can be reduced, and the observed trends in voltages and switching times provide useful points of comparison. It is important for researchers working on ferroelectric materials, particularly layered materials, to be aware of the prevalence of low energies for defect formation and migration, as well as the impact on device behaviors. The manuscript increases that awareness and adds an interesting element of

plausible photoactivated defect migration.

Reviewer #2

(Remarks to the Author)
The authors have supplemented HAADF-STEM characterization and density functional theory (DFT) calculations, substantiating the presence of selenium vacancies in α-In_2Se_3 and providing theoretical estimates of their formation energies and migration barriers. Although these results do not constitute direct evidence of vacancy migration and accumulation during the switching process, it is acknowledged that operando imaging under simultaneous cryogenic and illumination conditions remains a formidable experimental challenge. Furthermore, the authors have reported sub-microsecond switching speeds, representing a competitive value among previously demonstrated cryogenic memristive devices. The scope of this work has been appropriately focused on photo-addressable cryogenic digital memory, establishing a clear distinction from conventional low-temperature optical storage systems. Notably, the present device retains long-lived photo-activated switching capability well beyond the duration of photoexcitation, thereby enabling subsequent digital resistive switching—a functionality of considerable interest for cryogenic computing and quantum information systems.

The revised manuscript demonstrates a high level of novelty in its physical findings and a substantial degree of rigor in mechanism validation. The supplementary experiments and discussions have effectively addressed the principal concerns raised in the earlier review. While direct operando visualization of the switching mechanism remains an outstanding challenge, the current body of evidence provides a plausible mechanistic explanation for the observed photo-activated digital resistive switching behavior.

Reviewer #3

(Remarks to the Author)
The authors have provided a comprehensive point-by-point response and have resolved all my concerns. I have no additional comments. I recommend the acceptance of this paper.

Open Access This Peer Review File is licensed under a Creative Commons Attribution 4.0 International License, which permits use, sharing, adaptation, distribution and reproduction in any medium or format, as long as you give appropriate credit to the original author(s) and the source, provide a link to the Creative Commons license, and indicate if changes were made.
In cases where reviewers are anonymous, credit should be given to 'Anonymous Referee' and the source.
The images or other third party material in this Peer Review File are included in the article’s Creative Commons license, unless indicated otherwise in a credit line to the material. If material is not included in the article’s Creative Commons license and your intended use is not permitted by statutory regulation or exceeds the permitted use, you will need to obtain permission directly from the copyright holder.
To view a copy of this license, visit https://creativecommons.org/licenses/by/4.0/

Photo-activated digital resistive switching in a cryogenic photomemory

Response to Reviewers' Comments

We thank the reviewers for their insightful and constructive comments, which have helped us improve the quality and clarity of the manuscript. Below are our point-by-point responses to the comments. We have also made several modifications to the manuscript, which are highlighted in red in the revised manuscript. A list of major changes is summarized at the end of the report.

Reviewer #1 (Remarks to the Author):

The manuscript reports intriguing measurements of non-volatile resistance changes in three terminal In2Se3 devices as a function of drain-source bias, temperature, gate bias, and illumination. The central novel finding is a well-defined threshold for resistive switching under positive drain source bias (negative biases can reset the resistance) that is largest at low temperatures and can be controlled by the intensity and duration of illumination with visible and near-IR light. The threshold can also be tuned by the gate voltage, where majority carrier accumulation reduces the threshold. The underlying kinetics of the switching process support recognized programming schemes such as paired-pulse facilitation, albeit at rather low frequencies.

In Figure 3, the manuscript provides a possible explanation for the observed resistive switching. Specifically, it is attributed to the movement of positively charged selenium vacancies adjacent to electrodes such that the depletion region associated with the Schottky barrier contacts can be bridged by conducting filaments. This explanation is reasonable, but speculative; there is no direct experimental evidence for the accumulation of vacancies, nor for the potential drop (and hence resistance modulation) arising from (one of?) the contacts. Furthermore, given the reported polarization switching in Fig. 1c, which occurs at biases below those used in subsequent device measurements, the following statement on page 6 raises creates some confusion: "The photo-activated digital resistive switching we observe could, in principle, originate from two distinct microscopic processes: (i) a defect-mediated reorganization that creates localized conductive channels, or (ii) a defect-facilitated reversal of ferroelectric domains that drastically lowers the contact barrier. Critical experimental observations, however, strongly favor the former." The manuscript provides direct evidence for polarization reversal but not vacancy accumulation. Are both perhaps present? If so, how do they interact?

Overall, the studies are carefully done, the effects are clear, and the claims are appropriately qualified. The manuscript is of high quality with results that are of broad interest. That said, the potential impact on device applications is unclear. Rather high biases are applied for rather long times to achieve the behaviors of interest, and without clear evidence for the underlying switching mechanism, it is not possible to assess whether voltage thresholds and switching times may be reduced into practical regimes.

Response:

We thank the reviewer for recognizing the studies are carefully done and the manuscript is of high quality. Below are the responses to the concerns brought up by the reviewer.

1. **"There is no direct experimental evidence for the accumulation of vacancies,**

nor for the potential drop (and hence the resistance modulation) arising from (one of?) the contacts."

(1) We thank the reviewer for raising this critical concern. We fully agree that direct visualization of the formation and rupture of conductive channels near one of the contacts, typically via Scanning Transmission Electron Microscopy (STEM), Scanning Tunneling Microscopy (STM) or Scanning Probe Microscopy (SPM), provides the most conclusive evidence for the switching mechanism (*Yang et. al. Nat. Commun. 3, 732, 2012*; *Valov et. al. Nat. Mat. 11, 530-535, 2012*; *Yang et. al. Nat. Commun. 8, 15173, 2017*).

In our case, however, such experiments are particularly challenging because the abrupt resistive switching occurs only at low temperatures under in-situ illumination with above-bandgap light. Implementing operando imaging under these combined conditions is currently beyond the capabilities available to us. We therefore adopted an indirect approach, combining experimental observations with structural and computational analysis, to evaluate the vacancy accumulation mechanism.

- **Structural evidence:** We performed room-temperature HAADF-STEM imaging on thin α-In_2Se_3 flakes processed under similar conditions to our switching devices. The images, shown in Fig. R1, reveal the existence of vacancies. From the relative intensity analysis, we determine they are most likely middle-layer selenium vacancies, denoted as $V_{Se\text{-}3}$. We have adopted this notation throughout the revised manuscript to maintain consistency with the convention commonly used in Ref. 17.

- **Theoretical validation:** We performed DFT calculations on α-In_2Se_3 with different types of vacancy defects. As shown in Fig. R2, neutral $V_{Se\text{-}3}$ defects have the lowest formation energy. Upon above-bandgap illumination, photoexcited carriers get trapped by these defects (deep-level acceptors), leading to a change in defect charge state and a pronounced PPC response. We also calculate a migration barrier of approximately 0.76 eV using the climbing image nudged elastic band method (CI-NEB, Fig. R3). Although this barrier is substantial, the migration process is expected to be strongly assisted by the intense local electric fields near the Schottky contact and by the photoinduced change in defect charge state. Importantly, the electric field is concentrated within a narrow depletion region near the contact and can therefore be substantially larger than the nominal channel-averaged field. Field-driven ion migration over comparable barriers has previously been reported in several memristive systems under strong local electric fields (*Shang et al. Adv. Funct. Mater. 24, 2171–2179, 2014*; *Quesada et al. IEEE Electron Device Lett. 45, 2391–2394, 2024*).

Taken together, these observations do not constitute direct proof of vacancy accumulation, but they provide independent structural, theoretical, and transport evidences that strongly supports the proposed mechanism. Direct visualization of conductive-channel formation under cryogenic illumination would certainly be valuable, and we hope future advances in instrumentation will enable such measurements.

We have revised the discussion of the microscopic model to incorporate the newly obtained structural and theoretical evidences. The revised text reads as follows:

"We therefore propose a model in which photo-activated and field-controlled migration of charged selenium vacancies leads to the formation and rupture of conductive paths near the contacts[31]. Our scanning transmission electron microscopy (STEM) and density functional theory (DFT) results indicate that $V_{Se\text{-}3}$ defects are present and are

energetically favorable (Supplementary Fig. 16 and Fig. 17). Upon above-bandgap illumination, V_{Se-3} defects are expected to become negatively charged through the trapping of photogenerated carriers. DFT calculations further yield a migration barrier of approximately 0.76 eV for V_{Se-3} (Supplementary Fig. 18). In the presence of strong local electric fields near the Schottky contacts and photoinduced change in defect charge state, migration of these defects near the contacts is expected to be energetically accessible despite the micrometer-scale channel length[32] (upper inset of Fig. 3c). The abundant photogenerated electron-hole pairs may also lower the migration barrier for these charged vacancies by modulating the potential energy landscape. Within this picture, charged selenium vacancies can accumulate near one of the contacts (Fig. 3d), reorganize to create locally percolating conduction channels. Once established, these conducting paths persist long after the light is removed (Fig. 3e). A subsequent negative bias ruptures or depletes the vacancy accumulation (Fig. 3f), resetting the device to its HRS. When the bias is swept back to a positive value above the threshold voltage, the conductive paths reform (Fig. 3g), giving rise to the observed digital switching. In fact, field-driven ion migration over comparable barriers has been reported in several memristive systems under strong local electric fields at cryogenic temperatures[33,34]."

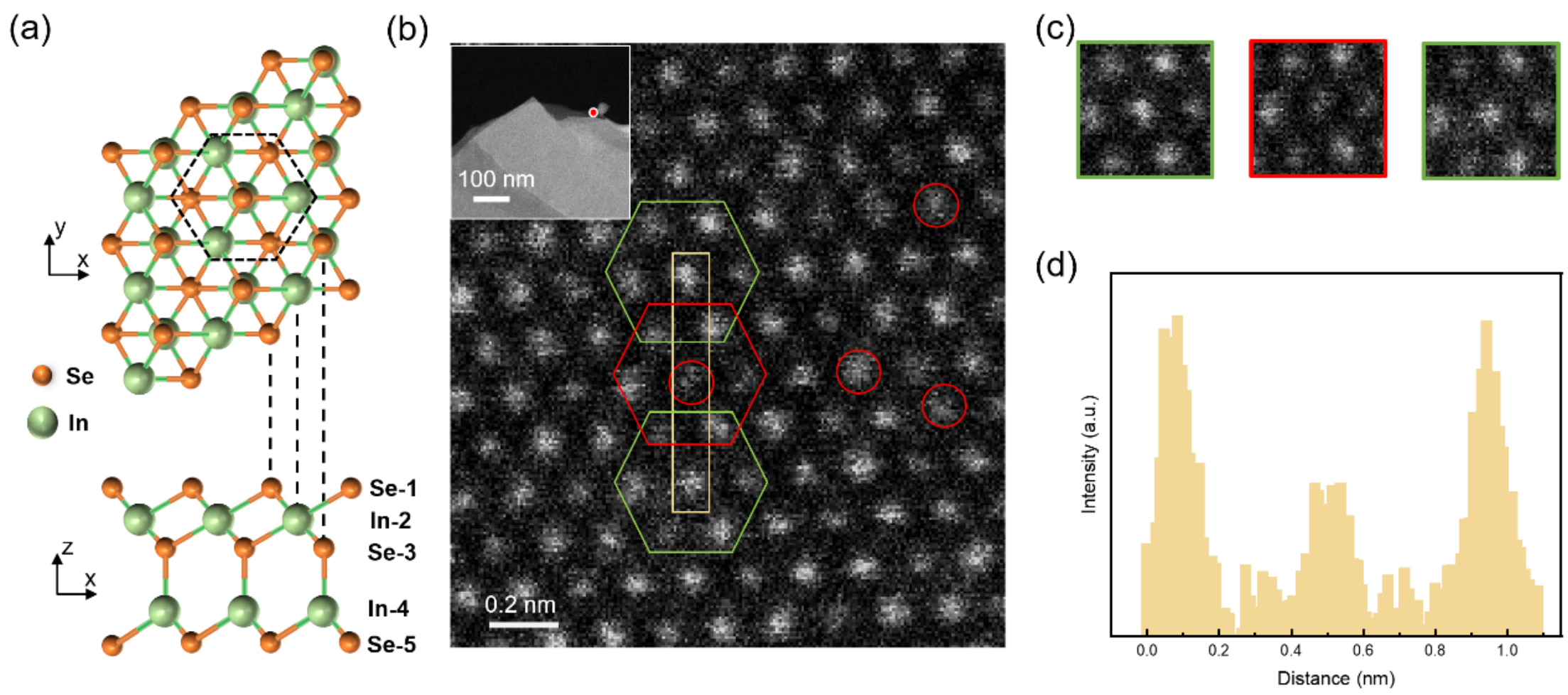

Fig. R1 | Structural analysis of α-In_2Se_3 thin flakes. a, Schematic crystal structure of α-In_2Se_3 in top and side views. **b,** Atomic-resolution top-view STEM image of an α-In_2Se_3 thin flake. The red and green polygons outline representative defective and pristine unit cells, respectively, and the red circles indicate the positions of the selenium vacancy (V_{Se-3}). The inset shows the image of the flake, with the red dot indicating the location where the STEM image was acquired. **c,** Representative pristine and defective regions extracted from **b** for comparison. **d,** Line profile of the STEM intensity extracted along the yellow box in **b**, the peak intensity at the center of the profile is approximately half of the at the two neighboring sites, consistent with the presence of V_{Se-3} at the center of the box.

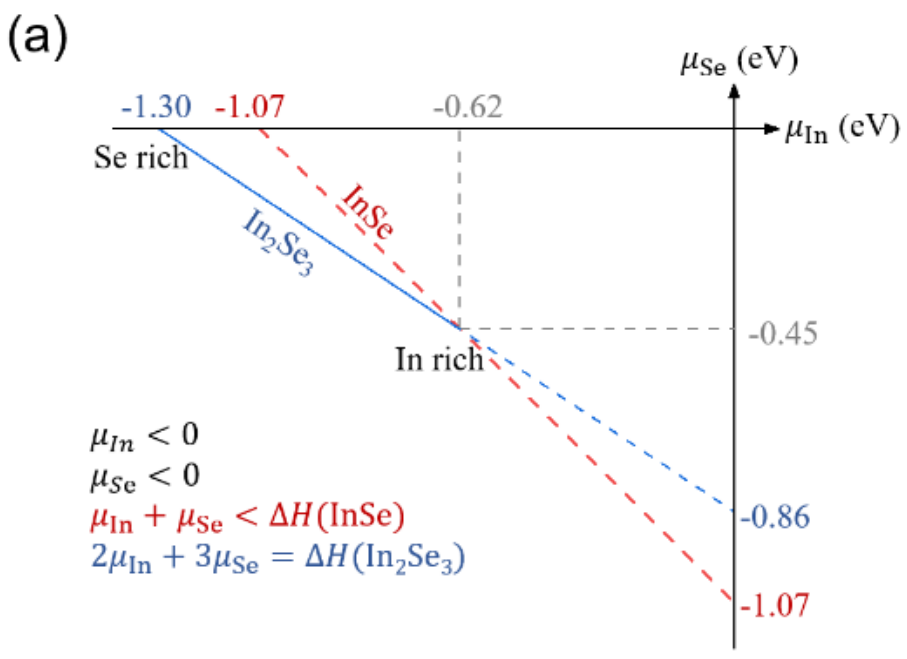

(b)

Formation Energy (eV)	In rich	Se rich
$V_{Se\text{-}1}$	1.73	2.18
$V_{In\text{-}2}$	2.78	2.10
$V_{Se\text{-}3}$	0.25	0.70
$V_{In\text{-}4}$	3.72	3.04
$V_{Se\text{-}5}$	0.84	1.29

Fig. R2 | Formation energies of different vacancy defects in α-In_2Se_3. a, Chemical potential phase diagram defining the thermodynamically allowed In-rich and Se-rich growth conditions for α-In_2Se_3. **b,** Calculated formation energies of different vacancy defects under the In-rich and Se-rich limits.

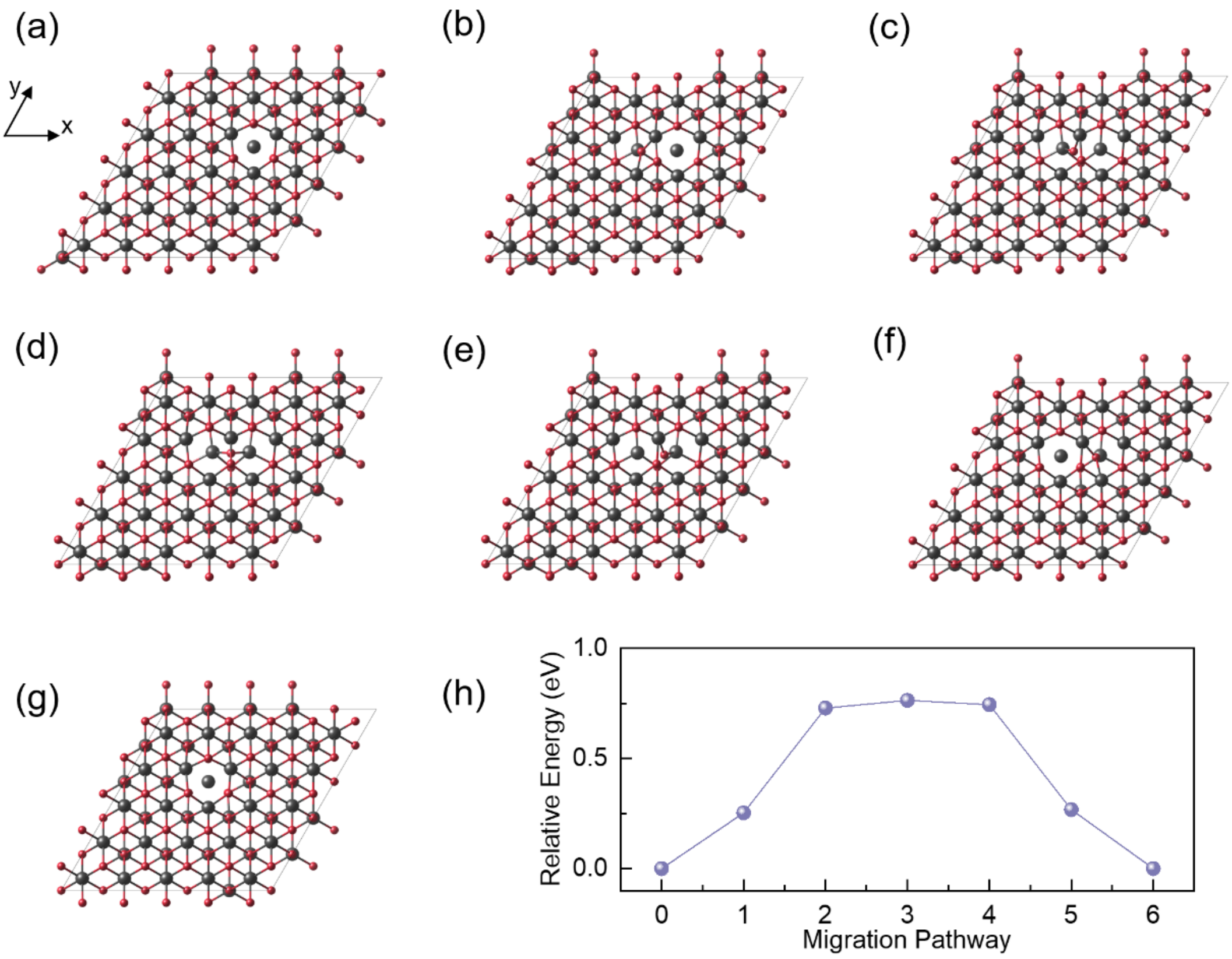

Fig. R3 | Simulation of the migration barrier for a single $V_{Se\text{-}3}$ defect. a-g, Atomic configurations along the migration pathway of a $V_{Se\text{-}3}$ defect. **h,** The calculated relative energy profile corresponding to the migration pathway shown in **a-g**.

(2) Regarding the potential drop near one of the contacts (not necessarily for both contacts), we agree that a direct spatial mapping of the electrostatic potential would be highly desirable. While such measurements are not available in the present work, several observations indicate that transport is strongly influenced by the contact regions in the devices exhibiting photo-activated digital switching.

First, devices shown photo-activated digital resistive switching typically exhibit exceptionally large two-terminal resistances and strongly nonlinear ***I-V*** characteristics even at room temperature. Second, similar contact pairs even on the same flake sometimes show much lower resistances. Third, a pronounced asymmetry is observed between positive and negative biases at low temperatures under illumination (Fig. 4a), suggesting that the two Schottky contacts are not electrically equivalent. Taken together, these observations indicate that the contact resistance varies substantially among different contacts and can dominate the overall device resistance.

Consequently, for devices exhibiting digital switching, we expect a significant fraction of the applied bias to drop within the narrow depletion region near the contacts rather than uniformly across the entire channel. This also naturally generates a strong local electric field that drives the migration and accumulation of charged selenium vacancies despite the micrometer-scale channel length.

2. **"The polarization switching in Fig. 1c occurs at biases below those used in subsequent device measurements."**

Thank you for pointing this out. Figure 1c is primarily intended to characterize the **out-of-plane** ferroelectricity of α-In_2Se_3 using PFM at **room temperature** (α-In_2Se_3 is known for the coupled out-of-plane and in-plane ferroelectricity). In these measurements, the voltage is applied between the tip and the graphite substrate underneath the α-In_2Se_3 flake. By contrast, the resistive switching measurements at **cryogenic temperatures** are performed in a **lateral** device geometry, where the bias is applied between the source and drain contacts. The direct comparison of the two voltages is therefore not appropriate.

However, one may note that the ferroelectric switching voltage in Fig. 1b (measured at room temperature) is comparable to the digital resistive switching threshold voltage in Fig. 2 (measured at 2 K). But as shown in Fig. 1b, Fig. S2 and Fig. S4, the transport signatures associated with ferroelectric switching evolve smoothly and gradually with bias at all temperatures, in marked contrast to the abrupt digital switching. Furthermore, the threshold voltage can be continuously tuned by electrostatic gating, and the gate-induced modulation is volatile. Such behavior is difficult to reconcile with a mechanism dominated by ferroelectric polarization reversal. Taken together, these observations suggest that the photo-activated digital resistive switching is more consistent with a vacancy-accumulation scenario, which provides a consistent framework for understanding the abrupt switching, strong illumination dependence, and gate tunability observed in our devices.

3. **"Are both polarization reversal and vacancy accumulation perhaps present? If so, how do they interact?"**

We thank the reviewer for this insightful point. Although the available experimental and theoretical evidence suggests that vacancy accumulation (formation of conductive channels) plays the dominant role in the observed photo-activated digital resistive switching, we agree that ferroelectric switching may also occur during the process and that the two mechanisms are likely coupled rather than independent.

Previous theoretical studies have revealed a strong polarization-defect coupling effect, where the vacancies can stabilize the ferroelectric phase and ferroelectric switching can also leads to defects transitions (*Ma et. al. Nano Lett. 25, 3726-3732, 2025*). These

results suggest a potentially bidirectional coupling between charged-defect dynamics and ferroelectric polarization. In our system, the accumulation of charged selenium vacancies may modify the local polarization landscape, while polarization switching may in turn affect the stability, charge state, or spatial distribution of vacancies. We have added a discussion of the interplay between vacancy accumulation and ferroelectric switching in the revised manuscript now.

"Nevertheless, the present evidence does not exclude a contribution from ferroelectric switching, and the interplay between defect dynamics and ferroelectric polarization remains an important open question that warrants further investigation."

4. "Rather high biases are applied for rather long times to achieve the behaviors of interest."

(1) High reset biases are applied for a long time in Figs. 2c & 2d.

In Fig. 2c&2d, following a negative reset pulse, the bias is continuously swept on the positive side, and the threshold voltage is extracted from the resulting switching event. We find that the threshold voltage depends strongly on the magnitude and duration of the preceding reset pulse, indicating that the reset process continuously modifies the internal state of the device. Within the proposed vacancy accumulation scenario, longer reset pulses drive charged selenium vacancies further away from the contact region, resulting in a more complete rupture of the conductive channel. Consequently, a higher positive bias is required to re-establish the conductive channel, leading to an increased threshold voltage. The pronounced time dependence of the threshold voltage over timescales approaching 100 s is therefore more consistent with a defect-migration process, in which the vacancy distribution evolves gradually with time.

Importantly, the long reset pulses are used to investigate threshold tunability rather than to achieve memory operation. As shown in Fig. S11, direct switching between the high- and low-resistance states can be repeatedly achieved using ±10 V voltage pulses with a duration of only 1 ms.

(2) The switching threshold voltage is high.

The switching thresholds in the main figures are relatively high, typically exceeding 5 V and reaching 20 V in some devices. However, these values should not be regarded as intrinsic limits of the switching mechanism. The threshold voltage is strongly device dependent and can vary with contact properties, density and distribution of intrinsic defects, and device dimensions. In addition, it can be tuned through both the reset protocol and electrostatic gating. For example, as shown in Fig. 2c, the switching threshold of device D1 can be reduced to 2.1 V following a (-4 V, 50 s) reset pulse.

(3) Set and reset voltage pulses of 1 ms are used in the memory demonstrations.

In Fig. S11, a sufficiently large bias of ±10 V was applied for 1 ms to demonstrate the endurance and retention characteristics of the device as a resistive switching memory. Although 1 ms pulses are sufficient for memory operations, they do not represent the intrinsic switching-speed limit of the device. We have performed additional switching-speed measurements on a new device (device D6), the results of which are shown in Fig. R4 of the revised manuscript. We find that the switching time decreases rapidly with increasing set voltage and reaches below 500 ns, which is currently limited by the temporal resolution of our measurement electronics. These results indicate that the intrinsic switching dynamics are substantially faster than suggested by the pulse

conditions used in the original memory demonstrations and are comparable to those reported for many memristive systems.

We have revised the manuscript to incorporate the measurements of switching speed as follows:

"To assess the potential of this photo-activated switching for non-volatile memory applications, we evaluated the key memory metrics including retention, endurance and switching speed. Supplementary Fig. 11 presents retention and endurance tests of the device measured at V_{ds} = 4 V. After 10000 s and 100 cycles, the current ratio between LRS and HRS remains high at 10^3. The switching speed was characterized on a similar device (device D6) by varying the set pulse duration at different amplitudes (see Supplementary Fig. 12). With a pulse amplitude of 18 V, the device switches from HRS to LRS within 500 ns. For larger pulse amplitudes, the switching timescale is expected to be even shorter. Such sub-microsecond switching speeds are among the fastest reported for memristors operate at cryogenic temperatures (Supplementary Table 1)."

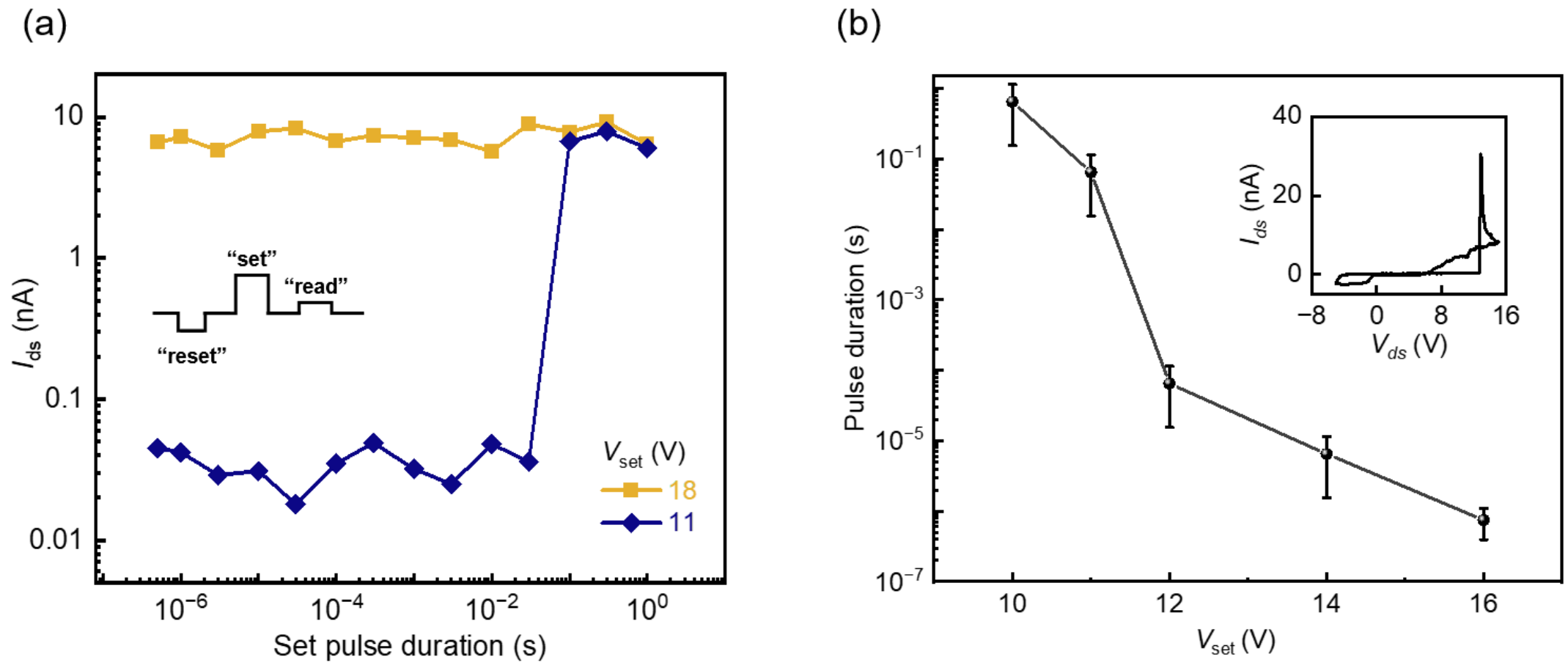

Figure R4 | Switching-speed measurements of α-In_2Se_3 (device D6). a, Read current (at V_{read} = 8 V) after representative set pulses (11 V and 18 V) of different durations. Prior to the set pulse, a reset pulse of -9 V, 1 s was applied. **b,** Critical set pulse duration as a function of V_{set}. The critical set pulse duration is identified where the device switches from high- to low-resistance states.

Reviewer #2 (Remarks to the Author):

Recommendation: Minor revision

The manuscript by Wang et al. reports the discovery of a photo-activated digital resistive switching effect in α In2Se3 lateral devices operating at cryogenic temperatures. The authors demonstrate that a single optical pulse enables digital resistive switching between high- and low-resistance states, with threshold voltages tunable via electrical reset pulses and electrostatic gating. The same defect-mediated mechanism also gives rise to a giant photoresponse, electrically resettable persistent photoconductivity, and cryogenic synaptic plasticity. The work is novel, technically sound, and of potential interest to the broad readership of Nature Communications. However, several issues remain that should be addressed before publication.

Response:

We thank the reviewer for recognizing our work is novel, technically sound and of potential interest to the broad readership of Nature Communications. Below are the point-by-point responses to their comments.

1. Regarding the microscopic mechanism, the authors favor a conductive channel formation mechanism over ferroelectric switching. However, direct imaging of the conductive filament (e.g. via in-situ TEM or conductive AFM) would make the arguments more convincing. Please comment on the feasibility of such experiments and, if not feasible, clarify what indirect evidence most strongly supports their model.

Response: We thank the reviewer for this helpful suggestion. We agree that direct imaging of the conductive channel formation and rupture, for example through STEM, conductive AFM, or related techniques, would make the microscopic switching mechanism more convincing and further strengthen the proposed interpretation.

In the present system, however, such experiments are particularly challenging because the abrupt resistive switching occurs only at low temperatures under in-situ illumination with above-bandgap light. Implementing operando imaging under these combined conditions is currently beyond the capabilities available to us. We therefore sought to evaluate the proposed mechanism through a combination of structural characterization, first principal calculations and transport measurements. Among the indirect evidences supporting the vacancy-accumulation scenario, we consider the following observations to be particularly important:

- **Structural evidence:** We performed room-temperature HAADF-STEM imaging on thin α-In_2Se_3 flakes processed under similar conditions to our switching devices. The images, shown in Fig. R5, reveal the existence of vacancies. From the relative intensity analysis, we determine they are most likely middle-layer selenium vacancies, denoted as $V_{Se\text{-}3}$. We have adopted this notation throughout the revised manuscript to maintain consistency with the convention commonly used in Ref. 17.

- **Theoretical validation:** We performed DFT calculations on α-In_2Se_3 with different types of defects. As shown in Fig. R6, neutral $V_{Se\text{-}3}$ defects have the lowest formation energy. Upon above-bandgap illumination, photoexcited carriers get trapped by these defects (deep-level acceptors), leading to a change in defect charge state and a pronounced PPC response. We also calculate a migration barrier of approximately 0.76 eV using the climbing image nudged elastic band method (CI-NEB, Fig. R7). Although

this barrier is substantial, the migration process is expected to be strongly assisted by the intense local electric fields near the Schottky contact and by the photoinduced change in defect charge state. Importantly, the electric field is concentrated within a narrow depletion region near the contact and can therefore be substantially larger than the nominal channel-averaged field. Field-driven ion migration over comparable barriers has previously been reported in several memristive systems under strong local electric fields (*Shang et al. Adv. Funct. Mater. 24, 2171–2179, 2014*; *Quesada et al. IEEE Electron Device Lett. 45, 2391–2394, 2024*).

- **Transport evidence:** We directly compared the photo-activated digital switching with ferroelectric switching measured in the same material system. As shown in Fig. 1b, Fig. S2, and Fig. S4, the transport signatures associated with ferroelectric switching evolve smoothly and gradually with bias, both at room temperature and under cryogenic conditions. Similarly, gate-induced ferroelectric switching does not produce abrupt resistance changes. By contrast, the photo-activated switching exhibits an exceptionally sharp threshold and an abrupt transition between the high- and low-resistance states. In addition, the switching threshold can be continuously tuned by the magnitude and duration of the preceding reset pulse, indicating a slowly evolving internal state. Such long-timescale threshold evolution is naturally explained by the gradual redistribution of charged vacancies and is difficult to reconcile with a mechanism dominated by ferroelectric polarization reversal.

Taken together, these observations do not constitute direct proof of vacancy accumulation, but they provide **independent structural, theoretical, and transport evidences that strongly support the proposed mechanism**. Direct visualization of conductive-channel formation under cryogenic illumination would certainly be valuable, and we hope future advances in instrumentation will enable such measurements.

We have revised the discussion of the microscopic model to incorporate the newly obtained structural and theoretical evidences. The revised text reads as follows:

"We therefore propose a model in which photo-activated and field-controlled migration of charged selenium vacancies leads to the formation and rupture of conductive paths near the contacts[31]. Our scanning transmission electron microscopy (STEM) and density functional theory (DFT) results indicate that $V_{Se\text{-}3}$ defects are present and are energetically favorable (Supplementary Fig. 16 and Fig. 17). Upon above-bandgap illumination, $V_{Se\text{-}3}$ defects are expected to become negatively charged through the trapping of photogenerated carriers. DFT calculations further yield a migration barrier of approximately 0.76 eV for $V_{Se\text{-}3}$ (Supplementary Fig. 18). In the presence of strong local electric fields near the Schottky contacts and photoinduced change in defect charge state, migration of these defects near the contacts is expected to be energetically accessible despite the micrometer-scale channel length[32] (upper inset of Fig. 3c). The abundant photogenerated electron-hole pairs may also lower the migration barrier for these charged vacancies by modulating the potential energy landscape. Within this picture, charged selenium vacancies can accumulate near one of the contacts (Fig. 3d), reorganize to create locally percolating conduction channels. Once established, these conducting paths persist long after the light is removed (Fig. 3e). A subsequent negative bias ruptures or depletes the vacancy accumulation (Fig. 3f), resetting the device to its HRS. When the bias is swept back to a positive value above the threshold voltage, the conductive paths reform (Fig. 3g), giving rise to the observed digital switching. In fact,

field-driven ion migration over comparable barriers has been reported in several memristive systems under strong local electric fields at cryogenic temperatures[33,34]."

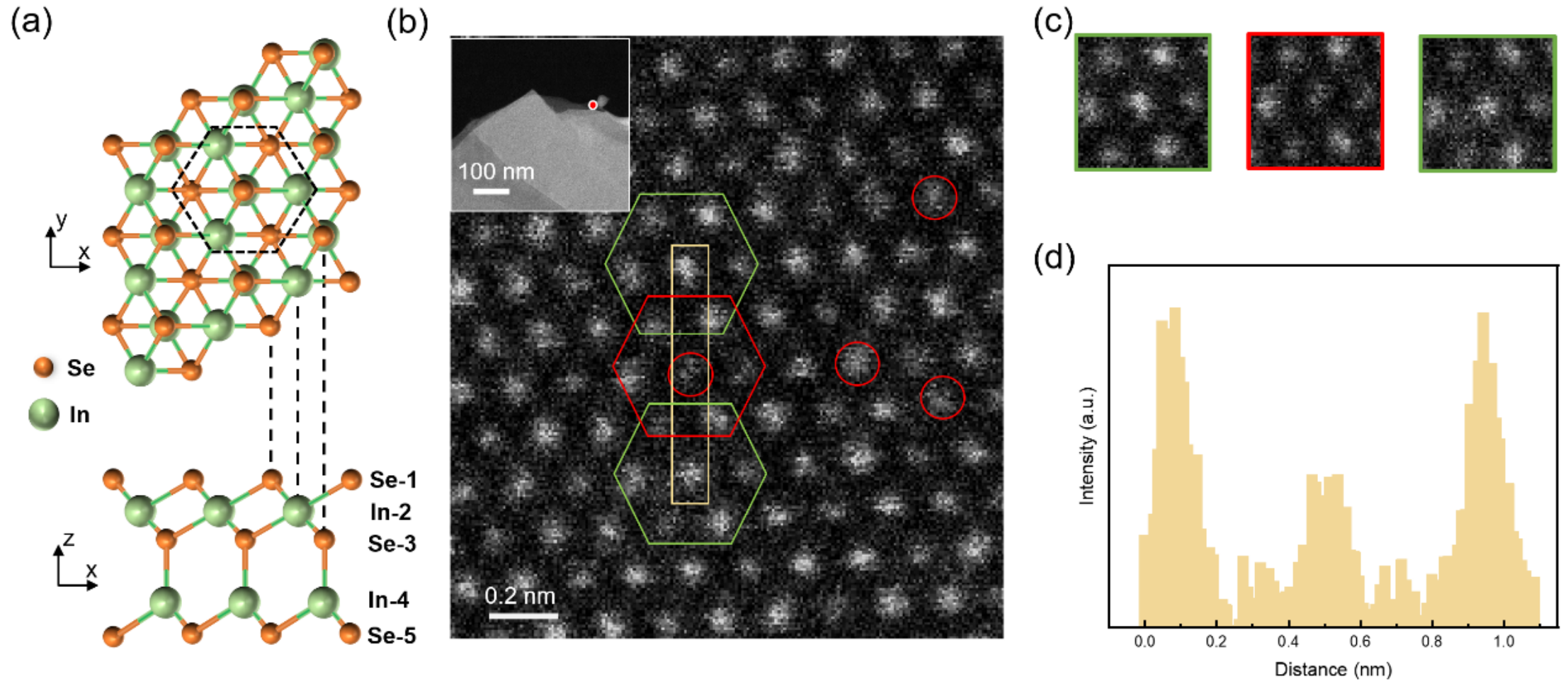

Fig. R5 | Structural analysis of α-In_2Se_3 thin flakes. a, Schematic crystal structure of α-In_2Se_3 in top and side views. **b,** Atomic-resolution top-view STEM image of an α-In_2Se_3 thin flake. The red and green polygons outline representative defective and pristine unit cells, respectively, and the red circles indicate the positions of the selenium vacancy (V_{Se-3}). The inset shows the image of the flake, with the red dot indicating the location where the STEM image was acquired. **c,** Representative pristine and defective regions extracted from **b** for comparison. **d,** Line profile of the STEM intensity extracted along the yellow box in **b**, the peak intensity at the center of the profile is approximately half of the at the two neighboring sites, consistent with the presence of V_{Se-3} at the center of the box.

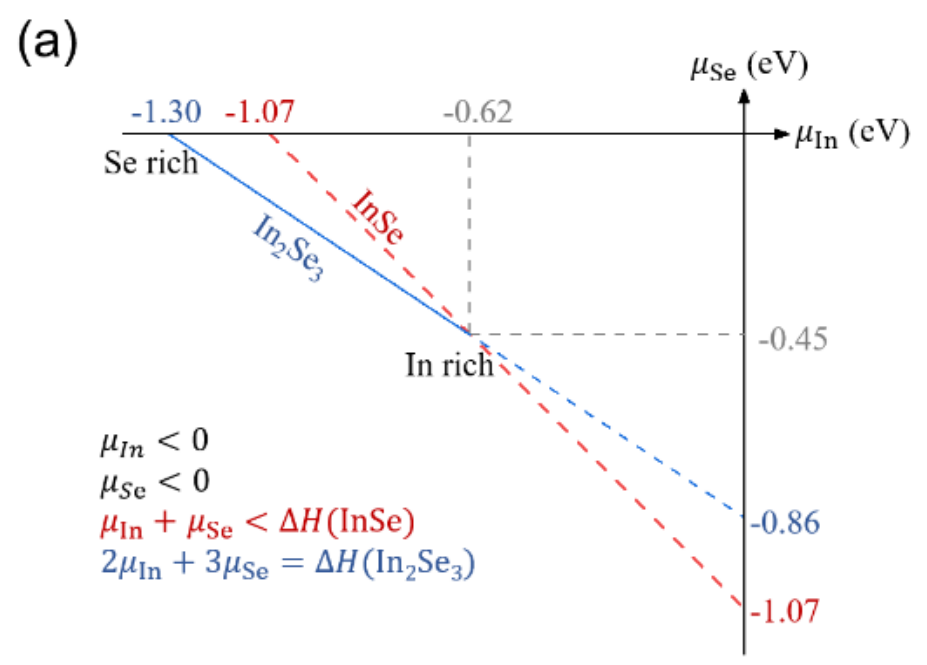

(b)

Formation Energy (eV)	In rich	Se rich
V_{Se-1}	1.73	2.18
V_{In-2}	2.78	2.10
V_{Se-3}	0.25	0.70
V_{In-4}	3.72	3.04
V_{Se-5}	0.84	1.29

Fig. R6 | Formation energies of different vacancy defects in α-In_2Se_3. a, Chemical potential phase diagram defining the thermodynamically allowed In-rich and Se-rich growth conditions for α-In_2Se_3. **b,** Calculated formation energies of different vacancy defects under the In-rich and Se-rich limits.

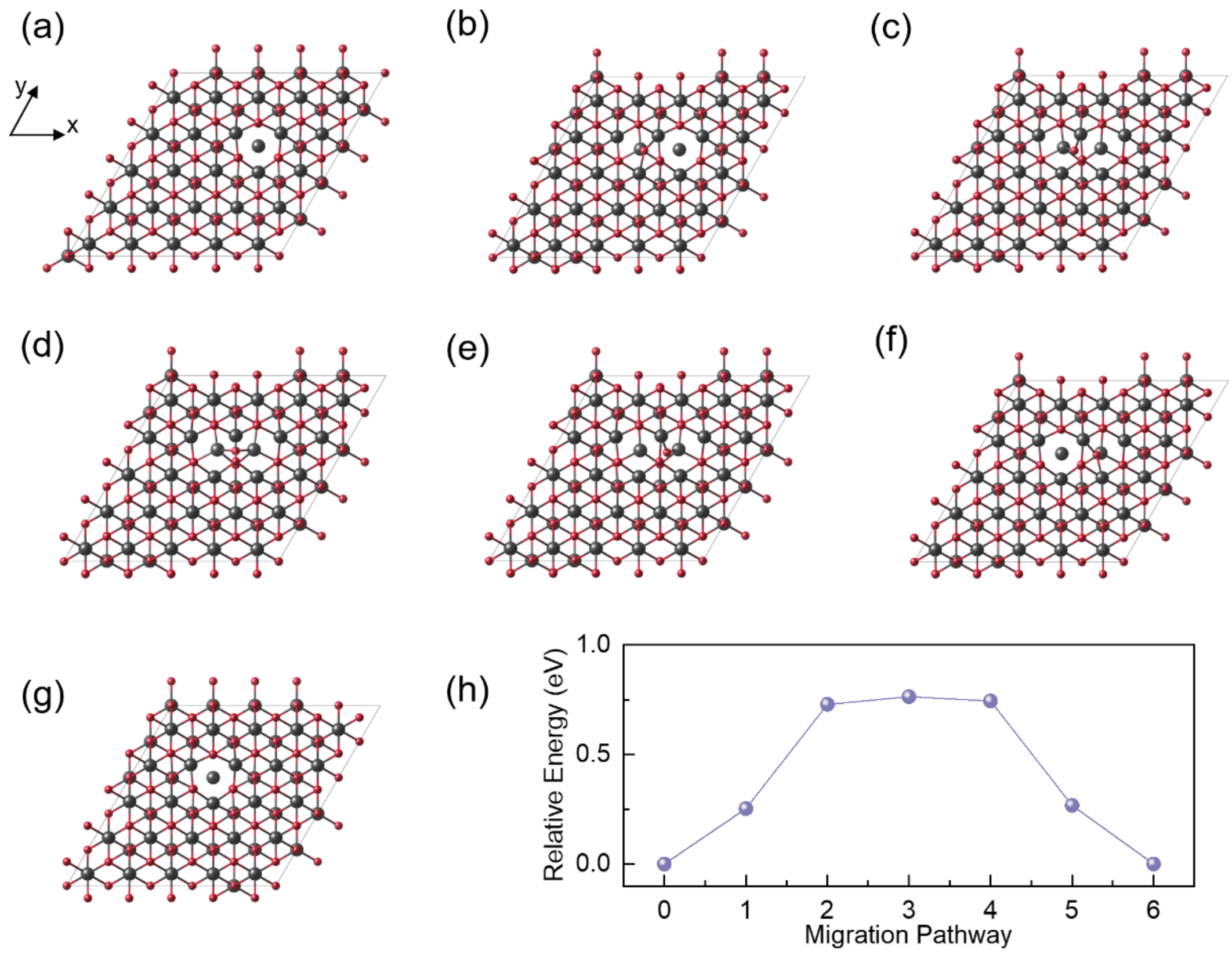

Fig. R7 | Simulation of the migration barrier for a single V_{Se-3} defect. a-g, Atomic configurations along the migration pathway of a V_{Se-3} defect. **h,** The calculated relative energy profile corresponding to the migration pathway shown in **a-g**.

2. Multiple capabilities have been demonstrated in the same device (cryogenic operation, optical activation, gate tuning, binary wavelength selectivity), and several applications (reconfigurable cryogenic memory, photodetector, neuromorphic functionalities) are mentioned without clearly prioritizing the most defensible one. The authors should identify and justify the most unique and compelling application for this particular device?

Response:

We thank the reviewer for this suggestion. Among the demonstrated capabilities and applications, the most unique and defensible application enabled by the newly discovered photo-activated digital switching effect is **an optically gated digital cryogenic memory**. Unlike conventional photomemory devices that store only a photoinduced conductance state, the present device retains a long-lived photo-activated switching capability, enabling subsequent digital resistive switching long after optical excitation. This functionality is particularly attractive for cryogenic computing and quantum-information systems, where optical addressing through low-heat optical fibers may offer a scalable alternative to conventional electrical control lines and help mitigate thermal-management challenges.

To clarify this point, we have added a discussion following the PPC measurements to emphasize the distinction between conventional photomemory behavior and the photo-activated digital resistive switching capability reported here.

"More importantly, the memory state in the present device is characterized not only by persistent photoconductivity, but also by a long-lived photo-activated digital resistive switching capability, distinguishing it from conventional cryogenic photomemory systems."

In addition, we revised the Conclusion section to more clearly summarize the key central finding of this work and its implications for cryogenic optoelectronic technologies. The revised Conclusion reads as follows:

"In summary, we report a novel photo-activated enduring digital resistive switching effect at cryogenic temperatures in α-In_2Se_3. We attribute this phenomenon to photo-activated and bias-driven migration of selenium vacancies, which creates conductive paths near the contact interfaces. The process can be dynamically modulated by optical pulses, reset voltage pulses, and an electrostatic gate, providing versatile control over the device's logic. These capabilities uniquely enable photo-addressable cryogenic digital memory with long retention, high endurance, and electrically tunable switching thresholds. In addition, the same physical mechanism gives rise to dramatically enhanced photoresponse, controllable PPC and synaptic functionalities. Together, these results highlight the potential of α-In_2Se_3 for multifunctional cryogenic optoelectronics, where memory, sensing, and signal-processing capabilities can be realized within a single cryogenic platform."

3. The authors reproduced the cryogenic photo-activated digital resistive switching behavior across multiple α- In2Se3 devices. However, key parameters such as Vset, on/off ratio and conductivity at negative bias appear to vary among these devices. What makes the differences?

Response:

Thank you for this important question. Indeed, variations in key parameters such as V_{set}, on/off ratio and conductivity at negative bias are observed across devices that exhibit photo-activated digital resistive switching behavior. We believe these differences primarily originate from variations in contact properties and defect density, with additional contributions from device dimensions:

(1) **Contact properties**: The proposed switching mechanism is highly sensitive to the local electric field near the contact region. Variations in contact cleanliness, interface quality, and local band alignment can modify the Schottky barrier height and depletion width at the α-In_2Se_3/Au interface, leading to significant differences in V_{set}, conductivity, and switching sharpness.

(2) **Intrinsic defect density**: Variations in the density of intrinsic defect (e.g. selenium vacancies) in the bulk crystals can be inherited by the exfoliated flakes, affecting V_{set}, on/off ratio and the overall conductivity.

(3) **Device dimensions**: Differences in flake thicknesses and channel lengths can alter the local electric field distribution and migration paths for the charged defects, thereby contributing to device-to-device variations.

We have discussed these differences in the revised Supplementary Information and included a table summarizing key parameters of the measured devices (Table Sxxx).

"Variations in V_{set}, on/off ratio and overall conductivity are observed across devices, which are attributed to differences in contact quality, intrinsic defect density, and device dimensions."

4. The authors state that in the dark, V_{set} is inaccessible. Have the authors attempted applying higher bias voltages to determine whether switching can be triggered in the dark? If not, what limits the bias range?

Response:

We thank the reviewer for raising this question. For device D1, we applied bias voltages up to 15 V at 2 K in the dark (Fig. S5) and observed no abrupt switching. For a new device (D6), we extended the bias range to ±30 V at 2 K in the dark, and still no abrupt switching occurred (Fig. R8). The absence of switching up to ±30 V suggests that the dark V_{set} may exceed the accessible bias range or the switching process may be strongly suppressed in the absence of photo-activated charged defects. Within our proposed mechanism, above-bandgap illumination generates photoexcited carriers that become trapped by selenium-vacancy defects, thereby modifying their charge state and enabling field-driven defect migration. Without illumination, the concentration of charged mobile defects is expected to be significantly reduced, making the formation of conductive channels much less favorable. Higher biases were avoided in our experiments to prevent dielectric breakdown of the α-In_2Se_3 flake or failure of the contacts.

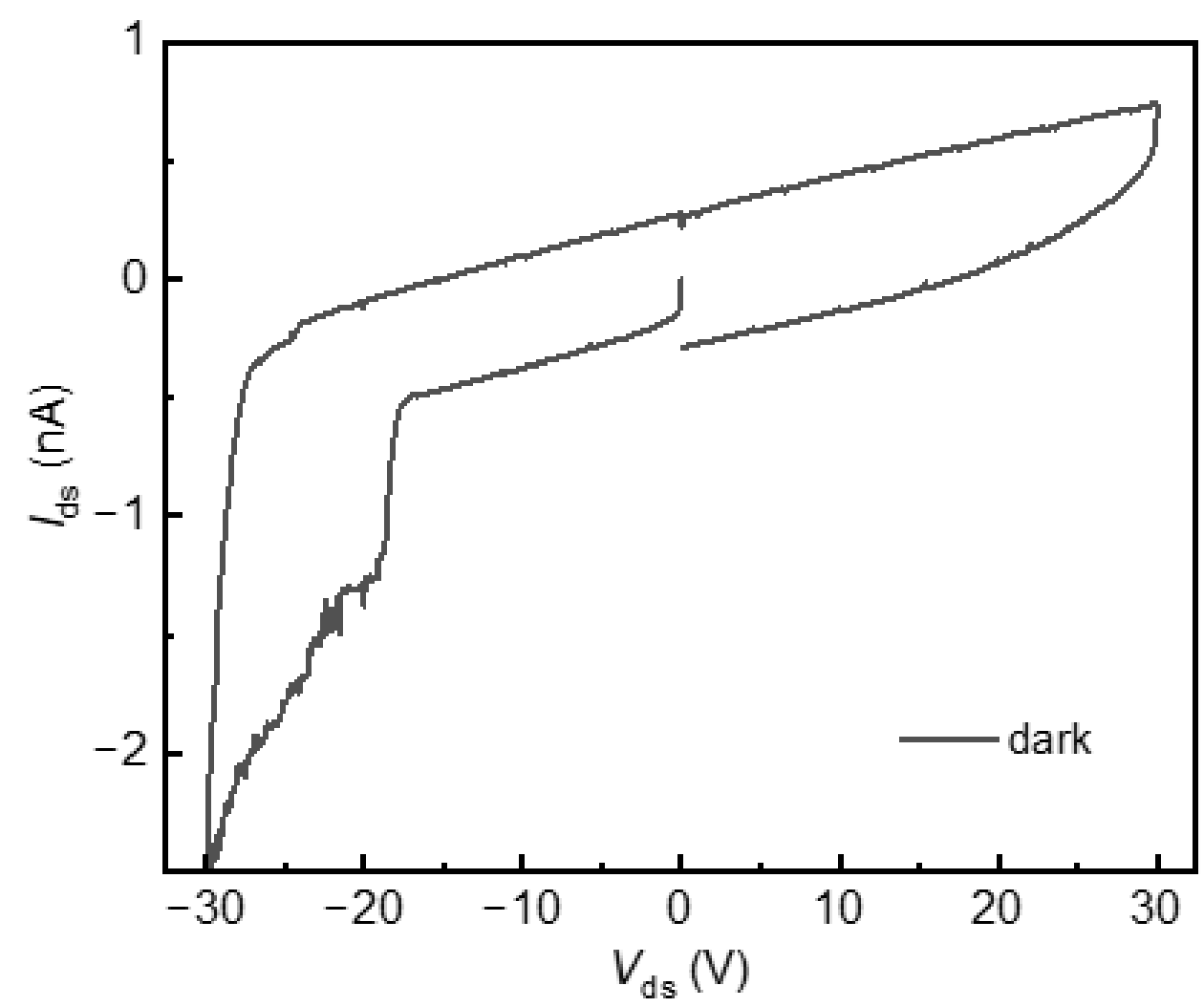

Fig. R8 | I–V sweep curve of device D6 in the dark.

5. In Fig. 2e, additional illumination under negative bias can effectively suppress the device's switching capability. Is this observation consistent with their microscopic model?

Response:

We thank the reviewer for this insightful question. We would like to clarify that illumination plays two different roles in our model. First, photoexcited carriers can be trapped by deep-level defects (selenium vacancies), modifying their charge state and giving rise to the persistent photoconductivity observed experimentally. Second, the abundant photocarriers present during illumination can modulate the potential energy landscape and facilitate the migration of charged vacancies.

Importantly, illumination itself does not determine the direction of vacancy migration. Rather, it enhances the mobility of charged vacancies, while the migration direction is

determined by the electric field. Under positive bias, the increased vacancy mobility promotes the accumulation of charged vacancies near the contact regions, facilitating the formation of conductive channels and reducing the switching threshold. In contrast, when illumination is applied under negative bias, the enhanced mobility promotes the rupture of conductive channels formed during the initial activation. Therefore, the combined electro-optical reset process efficiently suppresses the subsequent switching capability of the device.

To clarify this point, we have revised the discussion of the microscopic model in the manuscript as follows:

"The abundant photogenerated electron-hole pairs may also lower the migration barrier for these charged vacancies by modulating the potential energy landscape. Within this picture, charged selenium vacancies can accumulate near one of the contacts (Fig. 3d), reorganize to create locally percolating conduction channels."

6. The authors apply a bias voltage of 1 V during the initial illumination stage. Is there a specific reason for this choice? Does the photo-activation also occur at other bias voltages, or even in the absence of bias?

Response:

Thank you for raising the question. The bias voltage applied during the initial illumination stage is not unique to 1 V and can significantly influence the subsequent switching behavior of the device. As shown in Fig. R9, we measured the ***I-V*** characteristics of device D1 after illuminating it under different bias voltages (V_{ini}). When the activation bias voltage decreases from 1 V to -3 V, V_{set} increases from 2.7 V to 8.9 V. Thus, the photo-activation also occurs at other bias voltages and even in the absence of bias. We chose 1 V for the main experiment because it yields a relatively low V_{set} for this device.

To clarify this point, we have added Supplementary Fig. 6 (b) and revised the main text accordingly. Specifically, the relevant sentence now reads:

"Figure 2a shows the ***I-V*** curve (highlighted in blue) of device D1 at 2 K after illuminating it for 5 seconds under V_{ds} = 1 V (white light LED, 0.14 mW/cm^2, unless otherwise specified; see Supplementary Fig. 6 for measurements with different incident powers and initial bias voltages, respectively)."

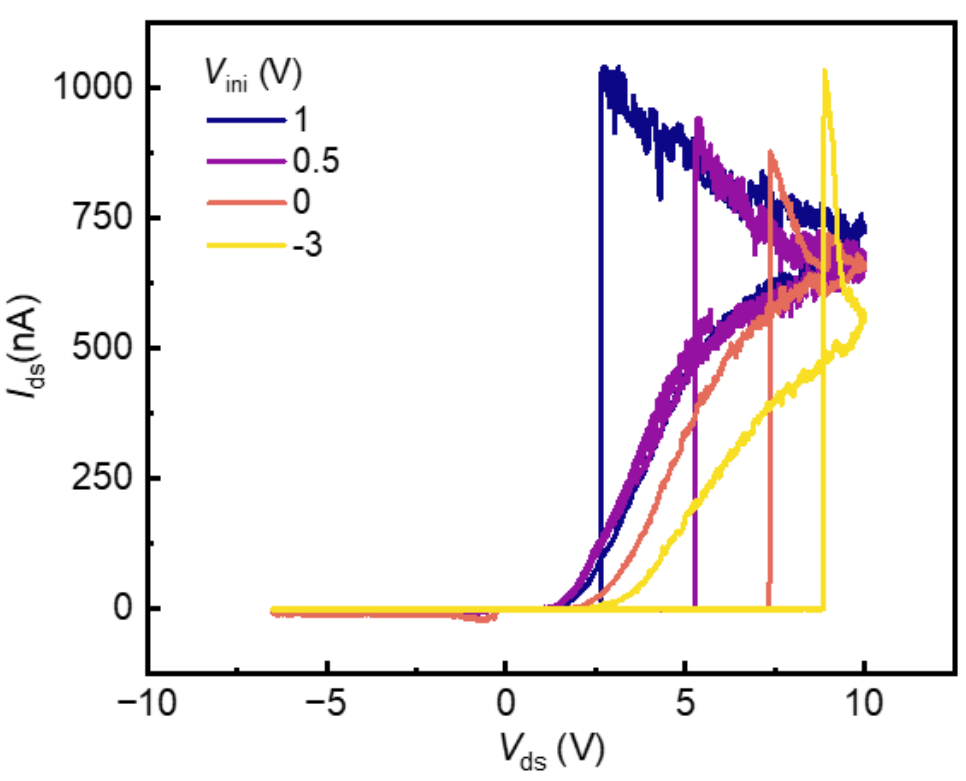

Figure R9 | I–V characteristics of device D1 after illuminating it (0.14 mW/cm^2, 5 s) at different bias voltages. The switching threshold increases from 2.7 V to 8.9 V when the activation bias voltage decreases from 1 V to -3 V.

7. The authors cite Ref 29 to explain the "crossing point" in I-V loops as a competition between ferroelectric switching and defect migration. Ref 29 refers to β'-In2Se3, whereas this work focuses on the α-phase. While the mechanisms are related, the structural differences (e.g., the interlocking of in-plane and out-of-plane polarization in α-phase) may lead to different migration barriers.

Response:

We thank the reviewer for this careful observation. Indeed, Ref. 29 refers to β'-In_2Se_3, a different polymorph from α-phase studied in our work, and the migration barriers may differ between the two different phases. In the original manuscript, we cited this reference to highlight that a similar crossing behavior has been observed in another In_2Se_3 polymorph, which was interpreted as arising from the interplay between ferroelectric switching and defect migration. We agree that the specific migration barriers and the detailed coupling between ferroelectricity and defect motion may different in α- In_2Se_3.

To avoid overinterpreting Ref. 29, we have revised the manuscript as follows:

"Such a crossing has been observed in another In_2Se_3 polymorph before and was attributed to the competition between ferroelectric switching and defect migration[29]."

8. The manuscript contains typographic errors, e.g. "defectscal" in the abstract. Please carefully proofread the entire text.

Response:

Thank you for the careful reading. We have corrected "defectscal" to "optical" in the abstract. Additionally, we have thoroughly proofread the entire manuscript and corrected all other typographical errors we identified.

Reviewer #3 (Remarks to the Author):

The authors report photo-activated resistive switching in In_2Se_3 at 2 K. While the physical concept is interesting, the manuscript lacks direct mechanistic evidence and falls short on practical memory metrics. I recommend major revisions based on the following points:

Response:

We thank the reviewer for acknowledging that the physical concept is interesting and for raising constructive concerns regarding mechanistic evidence and practical memory metrics. Below we provide point-by-point responses to each of their questions.

1. The mechanism of the proposed microscopic model inferred solely from I-V curves. Please provide direct microstructural evidence (e.g., TEM images) or DFT calculations.

Response: We thank the reviewer for this very valuable suggestion. We agree that the microscopic mechanism should be supported by structural or theoretical evidences in addition to transport measurements. To address this concern, we have performed both HAADF-STEM characterization and first-principles calculations, and incorporate the results to the revise manuscript.

- **Structural evidence:** We performed room-temperature HAADF-STEM imaging on thin α-In_2Se_3 flakes fabricated under similar conditions to our switching devices. The images, shown in Fig. R10, reveal the existence of vacancies. Quantitative intensity analysis indicates that these defects are predominantly middle-layer selenium vacancies, denoted as $V_{Se\text{-}3}$. We have adopted this notation throughout the revised manuscript to maintain consistency with the convention commonly used in Ref. 17. This provides the direct evidence for the existence of intrinsic selenium-vacancy defects in our material system.

- **Theoretical validation:** We further carrier out DFT calculations on α-In_2Se_3 to evaluate the stability and electronic properties of different vacancy configurations. As shown in Fig. R11, neutral $V_{Se\text{-}3}$ defects have the lowest formation energy. Upon above-bandgap illumination, photoexcited carriers get trapped by these defects (deep-level acceptors), leading to a change in defect charge state and a pronounced PPC response. We also calculate a migration barrier of approximately 0.76 eV using the climbing image nudged elastic band method (CI-NEB, Fig. R12). Although this barrier is substantial, the migration process is expected to be strongly assisted by the intense local electric fields near the Schottky contact and by the photoinduced change in defect charge state. Importantly, the electric field is concentrated within a narrow depletion region near the contact and can therefore be substantially larger than the nominal channel-averaged field. Field-driven ion migration over comparable barriers has previously been reported in several memristive systems under strong local electric fields (*Shang et al. Adv. Funct. Mater. 24, 2171–2179, 2014*; *Quesada et al. IEEE Electron Device Lett. 45, 2391–2394, 2024*).

Taken together, the STEM observations establish the presence of selenium vacancies, while the DFT calculations support their photoactivity and field-driven migrations. These results provide independent structural and theoretical support for the vacancy-accumulation mechanism proposed to explain the photo-activated digital resistive switching behavior.

We have revised the discussion of the microscopic model to incorporate the newly obtained structural and theoretical evidences. The revised text reads as follows:

"We therefore propose a model in which photo-activated and field-controlled migration of charged selenium vacancies leads to the formation and rupture of conductive paths near the contacts[31]. Our scanning transmission electron microscopy (STEM) and density functional theory (DFT) results indicate that $V_{Se\text{-}3}$ defects are present and are energetically favorable (Supplementary Fig. 16 and Fig. 17). Upon above-bandgap illumination, $V_{Se\text{-}3}$ defects are expected to become negatively charged through the trapping of photogenerated carriers. DFT calculations further yield a migration barrier of approximately 0.76 eV for $V_{Se\text{-}3}$ (Supplementary Fig. 18). In the presence of strong local electric fields near the Schottky contacts and photoinduced change in defect charge state, migration of these defects near the contacts is expected to be energetically accessible despite the micrometer-scale channel length[32] (upper inset of Fig. 3c). The abundant photogenerated electron-hole pairs may also lower the migration barrier for these charged vacancies by modulating the potential energy landscape. Within this picture, charged selenium vacancies can accumulate near one of the contacts (Fig. 3d), reorganize to create locally percolating conduction channels. Once established, these conducting paths persist long after the light is removed (Fig. 3e). A subsequent negative bias ruptures or depletes the vacancy accumulation (Fig. 3f), resetting the device to its HRS. When the bias is swept back to a positive value above the threshold voltage, the conductive paths reform (Fig. 3g), giving rise to the observed digital switching. In fact, field-driven ion migration over comparable barriers has been reported in several memristive systems under strong local electric fields at cryogenic temperatures[33,34]."

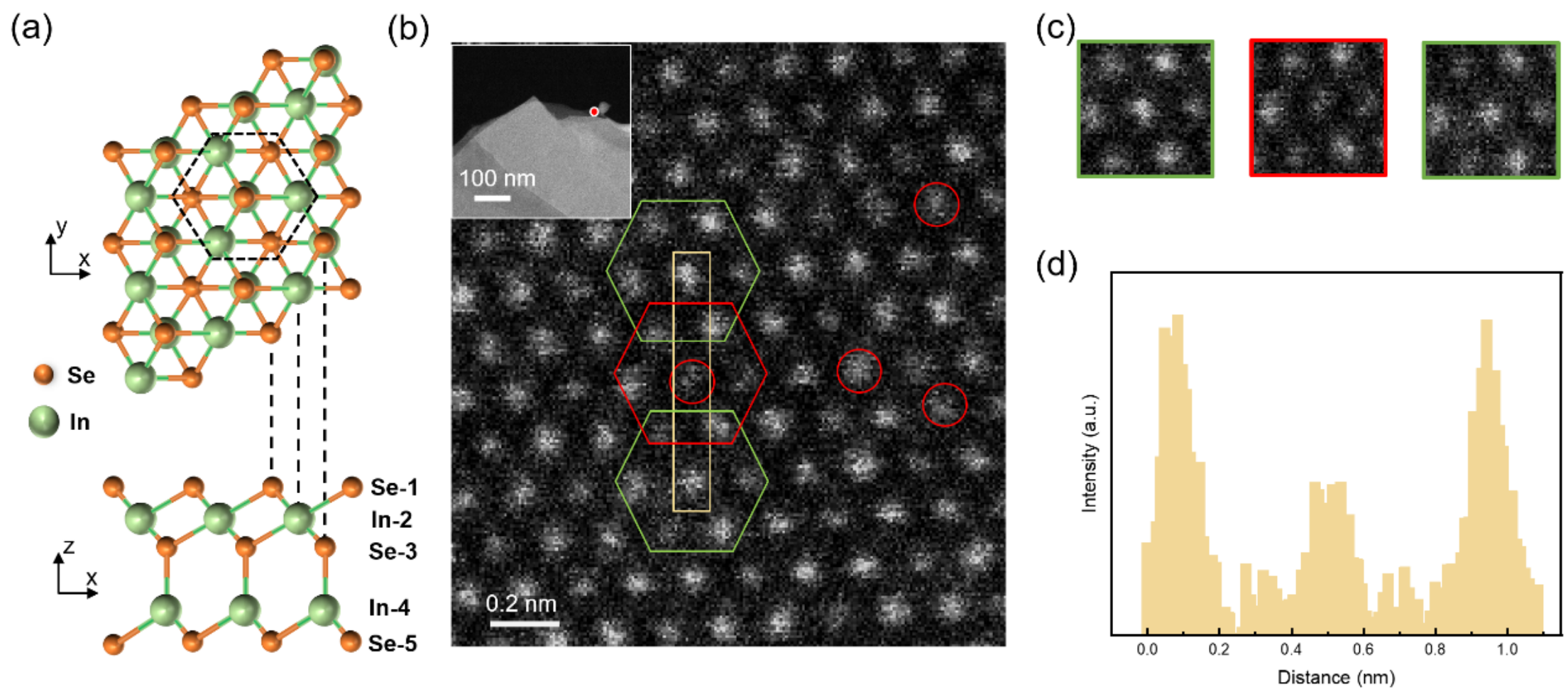

Fig. R10 | Structural analysis of α-In_2Se_3 thin flakes. a, Schematic crystal structure of α-In_2Se_3 in top and side views. **b,** Atomic-resolution top-view STEM image of an α-In_2Se_3 thin flake. The red and green polygons outline representative defective and pristine unit cells, respectively, and the red circles indicate the positions of the selenium vacancy ($V_{Se\text{-}3}$). The inset shows the image of the flake, with the red dot indicating the location where the STEM image was acquired. **c,** Representative pristine and defective regions extracted from **b** for comparison. **d,** Line profile of the STEM intensity extracted along the yellow box in **b**, the peak intensity at the center of the profile is approximately half of the at the two neighboring sites, consistent with the presence of $V_{Se\text{-}3}$ at the center of the box.

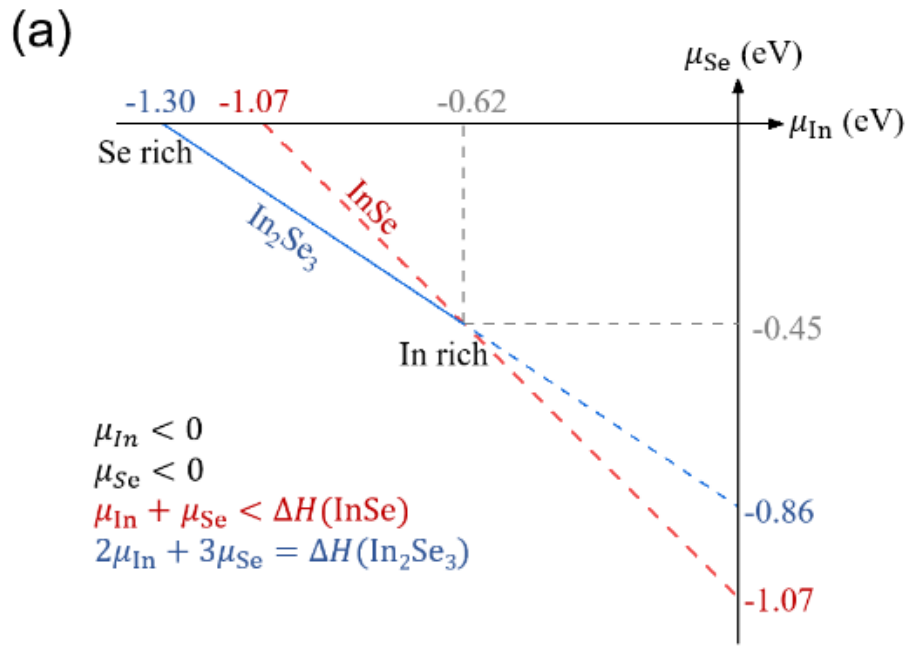

(b)

Formation Energy (eV)	In rich	Se rich
$V_{Se\text{-}1}$	1.73	2.18
$V_{In\text{-}2}$	2.78	2.10
$V_{Se\text{-}3}$	0.25	0.70
$V_{In\text{-}4}$	3.72	3.04
$V_{Se\text{-}5}$	0.84	1.29

Fig. R11 | Formation energies of different vacancy defects in α-In_2Se_3. a, Chemical potential phase diagram defining the thermodynamically allowed In-rich and Se-rich growth conditions for α-In_2Se_3. **b,** Calculated formation energies of different vacancy defects under the In-rich and Se-rich limits.

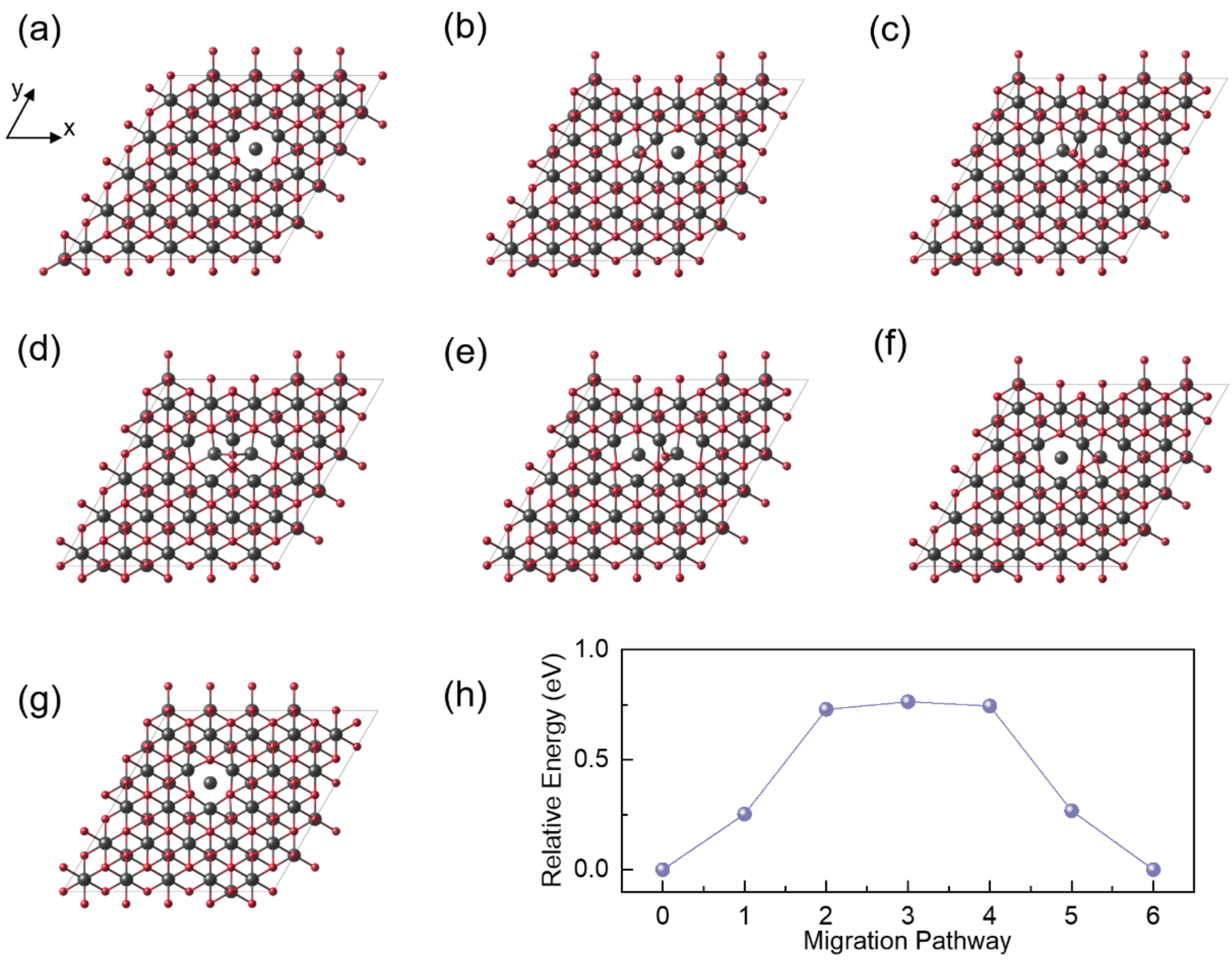

Fig. R12 | Simulation of the migration barrier for a single $V_{Se\text{-}3}$ defect. a-g, Atomic configurations along the migration pathway of a $V_{Se\text{-}3}$ defect. **h,** The calculated relative energy profile corresponding to the migration pathway shown in **a-g**.

2. It would be beneficial to experimentally measure the switching speed of the device and discuss the results.

Response:

We sincerely thank the reviewer for this insightful suggestion. To address this, we fabricated a new device (device D6) and systematically characterized its switching speed.

The measurement protocol was as follow: after optical activation, the device was first reset to the HRS using an electrical reset pulse (-9 V, 1 s). We then applied set pulses with varying amplitudes and progressively increasing the pulse duration (the minimum pulse duration achievable is 500 ns, limited by our instrumentation). After each setting pulse, we measure the steady-state current at a read voltage of 8 V to determine whether the device had switched to the LRS.

Figure R13a shows the readout current as a function of set pulse duration for pulse amplitudes of 11 V and 18 V. At an amplitude of 11 V, the device switches from HRS to LRS for pulse durations of ~0.1 s. At 18 V, however, the device switches reliably even at the shortest pulse (500 ns), indicating that the switching time scale is below 500 ns. Figure R13b summarizes the switching time as a function of the set pulse amplitude.

We have also added a discussion of these results to the main text:

"To assess the potential of this photo-activated switching for non-volatile memory applications, we evaluated the key memory metrics including retention, endurance and switching speed. Supplementary Fig. 11 presents retention and endurance tests of the device measured at V_{ds} = 4 V. After 10000 s and 100 cycles, the current ratio between LRS and HRS remains high at 10^3. The switching speed was characterized on a similar device (device D6) by varying the set pulse duration at different amplitudes (see Supplementary Fig. 12). With a pulse amplitude of 18 V, the device switches from HRS to LRS within 500 ns. For larger pulse amplitudes, the switching timescale is expected to be even shorter. Such sub-microsecond switching speeds are among the fastest reported for memristors operate at cryogenic temperatures (Supplementary Table 1)."

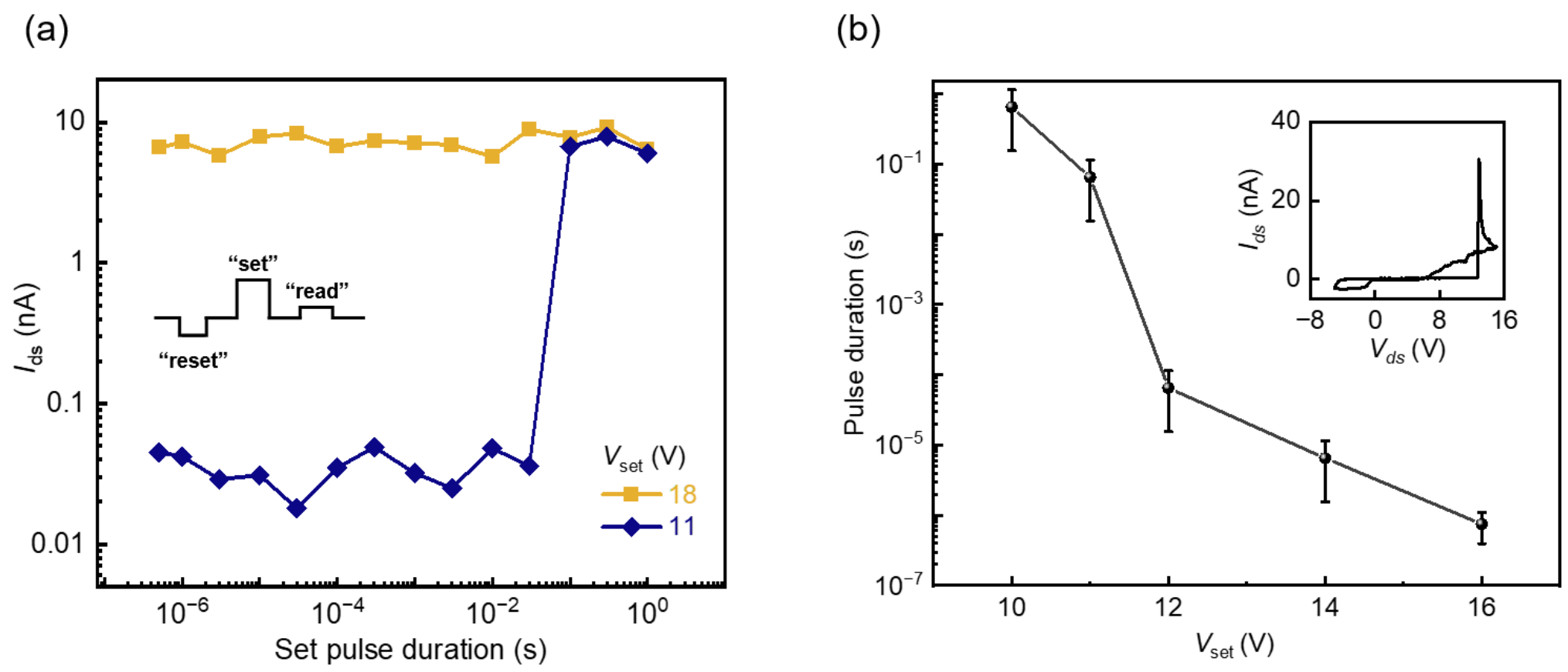

Figure R13 | Switching-speed measurements of α-In_2Se_3 (device D6). a, Read current (at V_{read} = 8 V) after representative set pulses (11 V and 18 V) of different durations. Prior to the set pulse, a reset pulse of -9 V, 1 s was applied. **b,** Critical set pulse duration as a function of V_{set}. The critical set pulse duration is identified where the device switches from high- to low-resistance states.

3. Supplementary Fig. 17 illustrates a simulated application of the device (ANN training and reasoning). The performance is measured at 2k. But the real ANN training and reasoning could not be performed at 2k. It differs significantly from practical scenarios.

Response:

We thank the reviewer for this comment. We agree that the ANN results shown in Supplementary Fig. 17 do not represent a hardware implementation of neural network

training and inference at 2 K. Rather, the simulation uses conductance states experimentally measured from our device at 2 K to evaluate whether the observed multilevel switching characteristics are, in principle, suitable for neuromorphic computing tasks.

Our intention was not to claim a complete cryogenic neuromorphic hardware demonstration, but instead to assess the computational utility of the experimentally obtained conductance states. In this sense, **the ANN simulation serves as a proof-of-concept benchmark** that connects the measured device characteristics to a representative machine-learning task. We agree that practical hardware implementation would require many additional considerations, including device-to-device variations, peripheral circuitry, and system-level operation at cryogenic temperatures.

To avoid overinterpreting the simulation results, we have clarified this point in the revised Supplementary Information:

"**Supplementary Fig. 17| Multilevel conductance states and handwritten digit recognition using an artificial neural network (ANN). a**, Optical potentiation and electrical depression tests of the device with optical and electrical pulses, displaying 36 distinct conductance levels (V_{read} = 2 V). These experimentally extracted conductance values serve as synaptic weights in the ANN simulation shown in **b-e**. **b**, Schematic of our ANN structure. The input image is decomposed into a 28×28 data matrix and flattened into a 784×1 vector (input layer). This vector is then processed through summation via a weight matrix to produce 128 intermediate variables, followed by transformation using a Rectified Linear Unit (ReLU) activation function (hidden layer). Finally, the output is mapped to digits 0 through 9 (output layer), corresponding to the classification of the input image. The entire network was trained iteratively. **c**, Reconstructed images of the digit "2" after different training epochs. **d**, Confusion matrix after the 1st and 100th training epoch. **e**, Recognition accuracy and loss as a function of the training epoch. With increasing epoch number, the recognition accuracy steadily improves while the loss decreases, ultimately reaching ~91% and ~0.32, respectively, after 100 epochs. The simulation serves as a proof-of-principle demonstration that the observed multilevel conductance states are sufficient for neuromorphic image recognition, rather than a full hardware validation."

4. The device is compared only with other In_2Se_3-based works, which is insufficient. It is suggested adding a benchmarking table that compares the device with state-of-the-art photomemory devices at cryogenic temperatures across key metrics.

Response:

We thank the reviewer for this valuable suggestion. We agree that comparing only with other In_2Se_3-based works is insufficient to place our work in a broader context. Accordingly, we have added a new benchmarking table (Table R1 or Supplementary Table R3) comparing our device with representative photomemory devices operating at cryogenic temperatures, including key metrics such as retention time, ON/OFF ratio, optical programming conditions, and operating temperature.

While the device exhibits conventional cryogenic photomemory behavior, the primary novelty of the present work lies in a distinct phenomenon that emerges from the photomemory state, namely photo-activated digital resistive switching. In our device, trapped photocarriers create a persistent activated state that subsequently enables an abrupt resistive transition upon the application of an electrical bias. To the best of our

knowledge, comparable examples of cryogenic photo-activated digital resistive switching remain scarce in the literature.

We have revised the corresponding text in the revised main manuscript as follows:

"The transient photoresponse suggests that the device can retain the photoinduced conductance state for an extended period at cryogenic temperatures, thereby functioning as a cryogenic photomemory. The key performance metrics are benchmarked against representative cryogenic photomemory systems in Supplementary Table 3, providing a broader context for evaluating its retention characteristics and optical programming condition. More importantly, the memory state in the present device is characterized not only by persistent photoconductivity, but also by a long-lived photo-activated digital resistive switching capability, distinguishing it from conventional cryogenic photomemory systems."

Structure	Optical retention	On/off ratio	Optical conditions	Temperature	Reference
MoS_2 FET	30 days	10^2	29.6 mW/cm², 5min, 365 nm laser	6 K	[24]
GaAsN	4000 s	5	700 s, 945 nm LED	77 K	[25]
GeSbTe	10^4 s	1.5	1 mW, 3s, 880 nm laser	4.1 K	[26]
Sb_2Se_3	Hours	~10^5	10 mW/cm², 1 min, 520 nm	22 K	[27]
GaP	3000 s	NA	10 mW, 200 s, White light	50 K	[28]
SnO_2	6 s	1.3	60 μW/mm², 10 s, 405 nm laser	9 K	[29]
$ZnO_{1-\delta}$	152.5 s	10^3	485 μW/cm², 600 s, UV illumination	11 K	[30]
$Cd_{0.97}Mn_{0.03}Te$	Hours	10^4	10^{16} photons/cm²·s, 30 min, White light	40 K	[31]
β-Ga_2O_3	4×10^4 s	10^2	1 h, 600 nm laser	90 K	[32]
WS_2 FET	~5000 s	~500	0.5 mW, 20 min, 462 nm laser	20 K	[33]
Tl_6SeI_4	2 hours	1.6×10^4	2000 mW/cm², 60 s, 405 nm laser	104 K	[34]
α-In_2Se_3 FET	> 10^3 s	> 10^3	0.14 mW/cm², 5 s, White light	2 K	This work

Table R1 | Comparison of metrics of representative cryogenic photomemory systems

5. Please clarify the purpose of performing Gaussian fitting on the switching threshold.

Response:

Thank you for asking this question. The purpose of performing Gaussian fitting on the switching threshold is to quantitatively assess the reproducibility and statistical variation of V_{set} under identical experimental conditions. As shown in Supplementary Fig. 8, repeated measurements on device D1 yield a distribution of switching thresholds that can be reasonably described by a Gaussian function, with a standard deviation of 1.2 V.

This statistical provides a reference scale for the intrinsic cycle-to-cycle variation of the device. It therefore allows us to assess whether the changes in V_{set} induced by different reset pulses or gate voltages are statistically significant. For example, the tuning of Vset

from 2.1 V to 8.7 V in Fig. 2c is substantially larger than the intrinsic fluctuation, confirming that the observed threshold modulation arises from the applied control parameters rather than from stochastic device variability.

We have clarified this point in the revised Supplementary Figure caption:

"**Supplementary Fig. 8| Repeated *I–V* sweeps of device D1 at 2 K,** with the bias restricted to positive values following a single pulsed light illumination (**a**), and with identical optical resets before each measurement (**b**). The switching threshold consistently appears near 5 V. Inset: Statistical distribution of threshold voltages, well described by a Gaussian centered at 5 V with a standard deviation of ~1.2 V. The repeated measurements are used to quantitatively evaluate the reproducibility and statistical variation of V_{set} under identical conditions."

6. On page 1, line 29, "defectscal pulses" appears to be a typo. Please correct it.

Response:

Thank you for the careful reading. We have corrected "defectscal pulses" to "optical pulses" in the abstract. Additionally, we have thoroughly proofread the entire manuscript and corrected all other typos we identified.

List of major changes (text changes highlighted in red):

Abstract:

- We have corrected the typographical error "defectscal pulses" to "optical pulses". The intended sentence now reads:
  "Crucially, the switching threshold voltage can be continuously tuned over a wide range by the reset voltage pulse, additional optical pulses, and electrostatic gating, providing multimodal control over the memory logic."

Main text:

- We have shown additional measurements with different initial bias voltages, and the corresponding results are presented in Supplementary Fig. 6b. The corresponding text has therefore been revised to:

  "…see Supplementary Fig. 6 for measurements with different incident powers and initial bias voltages, respectively."

- We have clarified in the main text that devices exhibiting cryogenic memristive behavior all show large and nonlinear resistance at room temperature.

  "This cryogenic photo-activated digital resistive switching behavior is reproducibly observed in a subset of α-In_2Se_3 devices that exhibit high and strongly nonlinear resistances at room temperature (see Supplementary Fig. 9 for data of devices D3-D5)."

- We performed additional switching-speed measurements on a new device. The corresponding results are summarized in Supplementary Fig. 12. Accordingly, we have revised the main text as follows:

  "To assess the potential of this photo-activated switching for non-volatile memory applications, we evaluated the key memory metrics including retention, endurance and switching speed. Supplementary Fig. 11 presents retention and endurance tests of the device measured at V_{ds} = 4 V. After 10000 s and 100 cycles, the current ratio between LRS and HRS remains high at 10^3. The switching speed was characterized on a similar device (device D6) by varying the set pulse duration at different amplitudes (see Supplementary Fig. 12). With a pulse amplitude of 18 V, the device switches from HRS to LRS within 500 ns. For larger pulse amplitudes, the switching timescale is expected to be even shorter. Such sub-microsecond switching speeds are among the fastest reported for memristors operating at cryogenic temperatures (Supplementary Table 1)."

- To address the reviewer's concern regarding the insufficient evidences for the claimed mechanism, we have incorporated additional STEM characterization and DFT calculations. These results are summarized in Supplementary Fig. 16–18. Accordingly, we have revised the relevant discussion and microscopic mechansim in the main text as follows:

  "The photo-activated digital resistive switching observed here could, in principle, originate from two distinct microscopic processes: (i) a defect-mediated reorganization that creates localized conductive channels, or (ii) a defect-facilitated reversal of ferroelectric domains that drastically lowers the contact barrier. Critical experimental observations, however, strongly favor the former. First, the extreme sharpness of the transition (3 dec/mV) and the subsequent negative differential

resistance are characteristics of formation of percolative conductive channels, not typically observed in the more gradual resistance modulation of ferroelectric switching (see gate dependences in Supplementary Fig. 2b). Second, the volatile nature of gate-tunability of V_{set} aligns with electrostatic control of defect-mediated reorganization process, whereas gate tuning of ferroelectricity is expected to be non-volatile due to the coupled in-plane and out-of-plane ferroelectric order in α-In_2Se_3. Nevertheless, the present evidence does not exclude a contribution from ferroelectric switching, and the interplay between defect dynamics and ferroelectric polarization remains an important open question that warrants further investigation."

"We therefore propose a model in which photo-activated and field-controlled migration of charged selenium vacancies leads to the formation and rupture of conductive paths near the contacts[31]. Our scanning transmission electron microscopy (STEM) and density functional theory (DFT) results indicate that $V_{Se\text{-}3}$ defects are present and are energetically favorable (Supplementary Fig. 16 and Fig. 17). Upon above-bandgap illumination, $V_{Se\text{-}3}$ defects are expected to become negatively charged through the trapping of photogenerated carriers. DFT calculations further yield a migration barrier of approximately 0.76 eV for $V_{Se\text{-}3}$ (Supplementary Fig. 18). In the presence of strong local electric fields near the Schottky contacts and photoinduced change in defect charge state, migration of these defects near the contacts is expected to be energetically accessible despite the micrometer-scale channel length[32] (upper inset of Fig. 3c). The abundant photogenerated electron-hole pairs may also lower the migration barrier for these charged vacancies by modulating the potential energy landscape. Within this picture, charged selenium vacancies can accumulate near one of the contacts (Fig. 3d), reorganize to create locally percolating conduction channels. Once established, these conducting paths persist long after the light is removed (Fig. 3e). A subsequent negative bias ruptures or depletes the vacancy accumulation (Fig. 3f), resetting the device to its HRS. When the bias is swept back to a positive value above the threshold voltage, the conductive paths reform (Fig. 3g), giving rise to the observed digital switching. In fact, field-driven ion migration over comparable barriers has been reported in several memristive systems under strong local electric fields at cryogenic temperatures[33,34]."

- We have benchmarked our device against with state-of-the-art photomemory devices at cryogenic temperatures across key metrics. Accordingly, the relevant statement in the main text has been revised to:

  "The transient photoresponse suggests that the device can retain the photoinduced conductance state for an extended period at cryogenic temperatures, thereby functioning as a cryogenic photomemory. The key performance metrics are benchmarked against representative cryogenic photomemory systems in Supplementary Table 3, providing a broader context for evaluating its retention characteristics and optical programming condition."

- To address the reviewer's concern regarding the significance and potential applications of the present work, we have further clarified the distinction between the present device and conventional photomemory systems by emphasizing the central finding of this work. The following sentence has been added to the revised manuscript:

  "More importantly, the memory state in the present device is characterized not only

by persistent photoconductivity, but also by a long-lived photo-activated digital resistive switching capability, distinguishing it from conventional cryogenic photomemory systems."

Conclusion:

- We have revised the Conclusion section to more clearly summarize the key findings, highlight the central role of photo-activated digital resistive switching, and discuss the broader implications for cryogenic optoelectronic systems. The revised text reads as follows:

  "In summary, we report a novel photo-activated digital resistive switching effect in α-In_2Se_3 that persists for extended periods at cryogenic temperatures. We attribute this phenomenon to photo-activated and field-driven migration of selenium vacancies, which creates conductive paths near the contact interfaces. The process can be dynamically modulated by optical pulses, reset voltage pulses, and an electrostatic gate, providing versatile control over the device's logic. These capabilities enable photo-addressable cryogenic digital memory with long retention, high endurance, and electrically tunable switching thresholds. In addition, the same physical mechanism gives rise to dramatically enhanced photoresponse, controllable PPC and synaptic functionalities. Together, these results highlight the potential of α-In_2Se_3 for multifunctional cryogenic optoelectronics, where memory, sensing, and signal-processing capabilities can be realized within a single cryogenic platform."

Methods:

- We have added additional information about the preparation of the STEM samples, STEM measurements, and DFT calculations.

  "**Device fabrication.** … α-In_2Se_3 samples for STEM measurements were picked up by PVA stamps, then dropped down onto a silicon nitride support grid with a 15 nm thick membrane on the grid holes at 160 ºC."

  "**STEM measurements:** High-angle annular dark-field scanning transmission electron microscopy (HAADF-STEM) images were acquired using an FEI Titan Cubed G2 60−300 aberration-corrected S/TEM operated at an accelerating voltage of 300 kV, with a probe convergent semi-angle of 22.5 mrad, a beam current of 0.06 nA, and a camera length of 73 mm."

  "**DFT calculations:** DFT calculations were performed using the Vienna Ab initio Simulation Package (VASP)[35]. The projector augmented wave pseudopotential and Perdew-Burke-Ernzerhof exchange correlation functional were used[36,37]. The valence electrons considered included In ($4d^{10}5s^{2}5p^{1}$) and Se ($4s^{2}4p^{4}$). A vacuum layer of more than 20 Å was introduced in the out-of-plane direction to minimize spurious interactions between periodic images. The migration energy barrier of the Se-3 vacancy ($V_{Se\text{-}3}$) was calculated using the climbing image nudged elastic band (CI-NEB) method[38]. A 5×5×1 supercell containing 124 atoms (incorporating one Se-3 vacancy) was employed. Structural relaxations were performed until the forces on each atom converged to less than 0.02 eV/Å.

The formation energies for the five types of vacancy defects were calculated using the following equation : $\Delta H(w) = E_{tot}(w) - E_{tot}(host) + (E_i + \mu_i)$. Here, $E_{tot}(w)$ is the total energy of the 124-atom supercell containing a specific vacancy defect, and $E_{tot}(host)$ is the total energy of the pristine 125-atoms supercell. The term $(E_i + \mu_i)$ accounts for the chemical potential changes caused by atomic substitution during defect formation. E_i represent the chemical potential of element i in its most stable elementary state, and μ_i indicates the relative chemical potential, capturing the influence of environmental conditions on the defect formation process. In our calculations, both In-rich and Se-rich limits were considered. The chemical potential ranges were strictly constrained to avoid the precipitation of pure elemental In or Se, as well as the formation of the common competing secondary phase, InSe. Finally, the points along the solid blue line in Supplementary Fig. 17(a) align with all of the requirements."

References:

- We have added Refs. 33-38 to the revised manuscript.

Figures:

- We have changed the colors of In atoms in Fig. 1a and Fig. 3c-g from green to dark green.
- We have updated the color schemes in Fig. 1b, Fig. 2, Fig. 3a,b, and Fig. 4a,d in accordance with the editorial request.
- We have added a simplified model of the device in the middle of Fig. 3c to clarify possible difference between different contacts.

Supplementary:

- We have added supplementary Figs. 6b, 12, 16, 17, 18 to the supplementary information. The figure captions are updated to reflect this change and addressing some of the reviewer's questions.
- We have added supplementary Tables 1, 3 to compare the key performances of this device as a memoristor and as a conventional photomemory.
- We have added Refs. 4-10, 24-34 to the supplementary information.
